# Disruption of heme homeostasis by nuclear receptor Nur77 induces pyroptosis through granzyme B-dependent GSDMC cleavage

**DOI:** 10.1038/s41392-025-02528-w

**Published:** 2025-12-17

**Authors:** Liu-Zheng Wu, Ya-Ying Huang, Hai-Jing Hu, Wen-Bin Hong, Han Yan, Yuan-Li Ai, Xiang-Yu Mi, De-Yi Feng, Jian-Yi Guo, Yang Ding, Zai-Jun Liu, Bo Zhou, Li Xiao, Tianwei Lin, Fu-Nan Li, Xue-Qin Chen, Hang-Zi Chen, Qiao Wu

**Affiliations:** 1 State Key Laboratory of Cellular Stress Biology, School of Life Sciences, Xiamen University, Xiamen, Fujian China; 2 The First Affiliated Hospital of Xiamen University, School of Medicine, Xiamen University, Xiamen, China; 3 Fujian Provincial Key Laboratory of Innovative Drug Target Research, School of Pharmaceutical Sciences, Xiamen University, Xiamen, China

**Keywords:** Cell biology, Chemical biology

## Abstract

Pyroptosis plays a crucial role in physiological and pathological processes. As melanoma cells are resistant to apoptosis but express gasdermin proteins, it is appealing to counter melanoma with the induction of gasdermin-executed pyroptosis. GSDMC, initially cloned from metastatic melanoma cells, has been demonstrated as a potential executioner of pyroptosis. However, no lead compounds that trigger GSDMC-mediated pyroptosis have been reported, which limits the in-depth investigation of GSDMC functions. Here, we discovered a chemical compound, dodecyl 1H-benzo[d]imidazole-5-carboxylate (DdBIC), that targeted the nuclear receptor Nur77 to induce pyroptosis through cleaving GSDMC by granzyme B in melanoma cells. Upon DdBIC binding, Nur77 was translocated to the mitochondria to activate the hemoprotein SDHA to overconsume succinyl-CoA, subsequently disrupting the homeostasis of heme in the SDH complex and resulting in electron leakage to induce mito-ROS production. This mito-ROS signal was sensed by the mitochondrial protease OMA1 via oxidation, which led to downstream OPA1 cleavage and subsequent released into the cytoplasm. Cytosolic OPA1 activated PERK to induce the integrated stress response (ISR), which further activated granzyme B to cleave GSDMC, culminating in the induction of pyroptosis. Together, this study elucidates a signal cascade from Nur77-impaired homeostasis of heme metabolism to PERK-mediated ISR activation, and reveals a novel paradigm, by which granzyme B, rather than caspases, cleaves GSDMC for pyroptotic induction and provides a new strategy for the therapeutic treatment of melanoma by lead compound DdBIC.

## Introduction

Pyroptosis is a type of programmed cell death that plays important roles in pathogen invasion, antitumor immunity, and metabolic and neurological diseases. Gasdermins (GSDMs) have been identified as executioners of pyroptosis. The molecular mechanisms of pyroptosis are complex and diverse. In the classical pathway, pyroptosis is triggered by inflammasomes, mainly through GSDMD execution.^1,2^ In the nonclassical pathway, pyroptosis can be induced by other GSDMs and is not associated with inflammasomes. Owing to the different characteristics of GSDMs, the responses of GSDMs to diverse external and intracellular stresses still need to be further investigated.

Many studies have identified the caspase-mediated cleavages of GSDMs as a common checkpoint for pyroptosis. During pyroptosis, caspase 3 cleaves and activates GSDME to switch noninflammatory apoptotic death into more rapid and inflammatory pyroptotic death.^3,4^ GSDMC, which is expressed in different cancer cell lines, can be cleaved by caspase 8 in TNF-mediated pyroptosis.^5^ We also reported that the endocytosis of the death receptor DR6 induced by the metabolite α-KG led to pyroptosis through the recruitment of both pro-caspase-8 and GSDMC to a DR6 receptosome with subsequent caspase-8 cleavage of GSDMC.^6^ However, caspases are not the only proteases for GSDMs; other proteases also play key roles in pyroptosis. GSDMB, which is widely expressed in gastrointestinal and immune cells, is cleaved by granzyme A to induce pyroptosis.^7^ Granzyme B is also released from killer lymphocytes into target cells to cleave GSDME and trigger pyroptosis.^8^ Therefore, different proteases can cleave not only different GSDMs but also the same GSDMs under different circumstances.

Different GSDMs have different antitumor properties. GSDME has a broad spectrum of inhibitory effects on a variety of tumors, but its expression is usually silenced in tumor cells due to promoter methylation.^2^ More recently, we revealed a novel mechanism whereby mannose antagonizes GSDME-mediated pyroptosis through GlcNAc-6P-mediated activation of AMPK.^9^ This discovery led to the application of mannose for ameliorating adverse conditions during chemotherapy, providing a supplement that is simple, safe, and efficacious in relieving toxic side effects.^9^ Although GSDMC, which is ubiquitously expressed in various cancers, is known to be an inhibitor of cancer development,^6,10^ the regulatory mechanism by which GSDMC functions in pyroptosis has not yet been fully elucidated.

Heme is an iron-containing cyclic tetrapyrrole that plays a central role in diverse and essential processes, including electron transport, circadian rhythm, redox reactions, and gene expression.^11^ Heme synthesis is regulated by several metabolites and is inhibited by either itaconate or succinyl-CoA deficiency.^12^ Heme can intercalate into membranes and catalyze the production of reactive oxygen species (ROS).^13^ Cell fate determination is closely coupled with heme homeostasis. Free heme is a pro-oxidant, and its accumulation accelerates the production of mito-ROS. However, heme is also an indispensable prosthetic group for assembling complexes in the electron transport chain (ETC), such as the SDH complex, which prevents electron leakage and reduces mito-ROS production.^14^ Although heme metabolism is related to mito-ROS production, it is still unclear how an imbalance in heme homeostasis independent of its pro-oxidative function leads to cell death.

The nuclear receptor Nur77 (also known as TR3, NGF1-B, or NAK1) plays important roles in the development and maturation of cells, the proliferation and differentiation of immune cells, the regulation of glucose and lipid metabolism, and cell death, such as apoptosis and autophagy.^15–18^ The physiological ligands of Nur77 have not yet been identified. We identified the first Nur77 agonist, Cytosporone-B (Csn-B).^19^ On the basis of the framework of Csn-B, additional chemical compounds that target Nur77 but have different functions were discovered by our group, including TMPA, which can decrease glucose levels by regulating AMPK,^20^ and PDNPA, which not only has anti-inflammatory properties by modulating p38 phosphorylation^21^ but also has an inhibitory effect on the progression of hepatocellular carcinoma via ectosomes.^22^ Clearly, Nur77 is a viable target for modulating physiological and pathological processes.

The resistance of melanoma cells to apoptosis poses an obstacle to therapy. GSDMC was initially cloned from metastatic melanoma cells.^23^ However, the lead compounds that trigger GSDMC-mediated pyroptosis have not been reported. Here, a chemical compound, dodecyl 1H-benzo[d]imidazole-5-carboxylate (DdBIC), designed by our group, was shown to effectively induce GSDMC-dependent pyroptosis in melanoma cells. Using this compound as a tool, we found Nur77 to be the direct target of DdBIC. By interacting with SDHA after DdBIC binding, Nur77 was conducive to abnormal heme metabolism, which promoted electron leakage from the SDH complex and the production of mito-ROS in the mitochondria. As a consequence, mitochondrial OPA1 was cleaved by the mito-ROS-activated protease OMA1 and then released into the cytosol. Cytosolic OPA1 subsequently activated the integrated stress response (ISR), which ultimately activated granzyme B to cleave GSDMC for pyroptotic induction. Overall, this study not only elucidates a novel mechanism by which Nur77 is associated with GSDMC-mediated pyroptosis but also elucidates the regulatory network of the SDH complex and homeostasis of heme metabolism that contributes to pyroptosis. These findings thus provide a novel strategy for developing pyroptosis-inducing therapeutics.

## Results

### DdBIC induces GSDMC-mediated pyroptosis by impairing homeostasis of heme metabolism

**Fig. 1:**
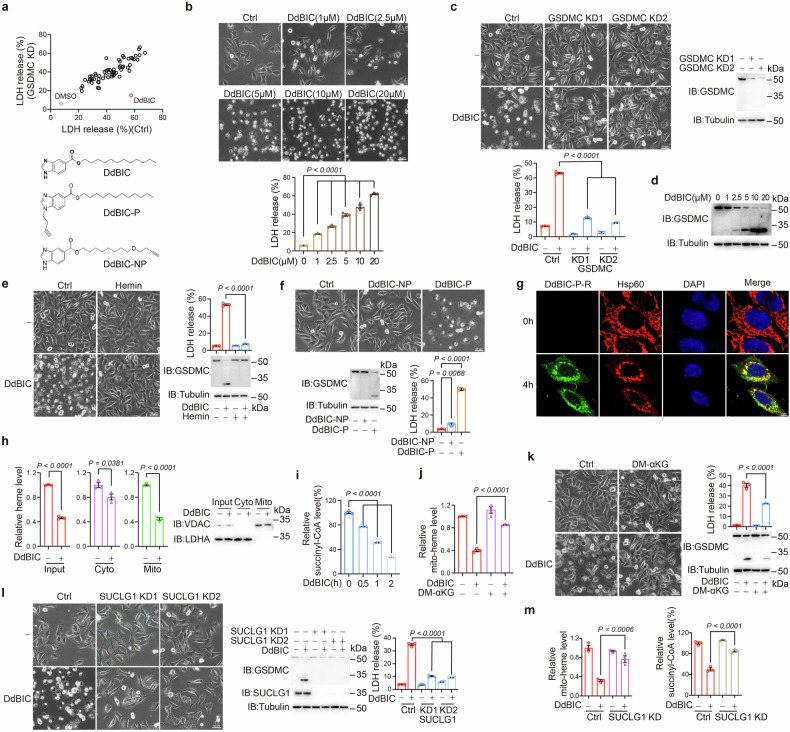
DdBIC induces GSDMC-mediated pyroptosis by regulating heme levels. Melanoma A375 cells were treated with DdBIC (20 μM) for 1 h to measure the level of succinyl-CoA, for 4 h to measure the level of heme, and for 8 h to assess pyroptotic features, unless otherwise specified. **a** Top, GSDMC-knockdown cells were treated with different compounds (n = 63) for 8 h, and LDH levels were detected. Bottom, chemical structures of DdBIC, the DdBIC probe (DdBIC-P) and its corresponding negative probe DdBIC-NP. **b** Cells were treated with DdBIC at the indicated doses to detect pyroptotic morphology and LDH levels. **c** In GSDMC-knockdown cells, pyroptotic morphology and LDH levels were evaluated. **d** DdBIC induced GSDMC cleavage in a dose-dependent manner. **e** Cells were cotreated with hemin (5 μM) and DdBIC to detect pyroptosis. **f** Cells were incubated with DdBIC-P or DdBIC-NP (100 μM) for 8 h, and pyrotopsis was performed as indicated. **g** DdBIC-P modified by a click reaction with rhodamine-N3 (DdBIC-P-R) was incubated with cells for 4 h. Mitochondria and nuclei were stained with an anti-Hsp60 antibody and DAPI. **h**, **i** Cells were treated with DdBIC, and the levels of cytosolic and mitochondrial heme (**h**) or succinyl-CoA (**i**) were shown. The cytosolic and mitochondrial fractions were removed to exclude contamination (**h**, right). **j**, **k** Cells were cotreated with DM-αKG and DdBIC to measure the levels of heme in mitochondria (**j**) and pyroptosis (**k**). **l**, **m** SUCLG1 was knocked down first in cells, and pyroptosis (**l**) and the levels of mitochondrial heme and succinyl-CoA (**m**) were detected. Statistics: two-way ANOVA with Tukey’s test to **c**, **e** and **j**–**m**; one-way ANOVA with Tukey’s test to **b**, **f** and **i**; unpaired two-tailed Student’s *t* test to **h**. *P* values are shown

To obtain GSDMC-mediated pyroptosis inducers, a classical pyroptotic morphological marker (cells with bubbles in the plasma membrane) was used to screen an in-house library of 2371 chemical compounds. The 63 compounds identified were further screened for LDH release as a marker in GSDMC-knockdown A375 cells. A compound, dodecyl 1H-benzo[d]imidazole-5-carboxylate (DdBIC), was demonstrated to be incapable of increasing LDH release in GSDMC-knockdown A375 cells (Fig. 1a). To further evaluate the DdBIC functions mediated through GSDMC, we perform several additional experiments. First, DdBIC effectively induced pyroptotic morphology and LDH release in a dose-dependent manner in four cancer cell lines (including melanoma A375 cells and M14 cells, breast cancer MDA-MB-231 cells and cervical cancer HeLa cells) (Fig. 1b and Supplementary Fig. 1a), as well as in other cancer cell lines (Supplementary Fig. 1b), indicating a broad spectrum of DdBIC in pyroptotic induction. Second, DdBIC-induced pyroptotic morphologies were not reversible in melanoma A375 cells treated with other types of cell death, including Z-VAD (apoptosis), NSA (necroptosis), Nec-1 (an RIPK1 inhibitor that indirectly inhibits necroptosis), Fer-1 and Lip-1 (ferroptosis), CQ (autophagy), and TTM (cuproptosis) (Supplementary Fig. 1c), indicating that DdBIC is specific for pyroptotic induction. Third, only the knockdown of GSDMC impaired DdBIC-induced cell death, not the knockdown of GSDMA, GSDMB, GSDMD, and GSDME, the other executioners of pyroptosis^24^ (Fig. 1c and Supplementary Fig. 1d). Fourth, DdBIC-induced pyroptosis was associated with GSDMC cleavage in various cancer cell lines (Fig. 1d and Supplementary Fig. 1e), and knockdown of GSDMC suppressed DdBIC-induced pyroptosis in three tumor cell lines (Supplementary Fig. 1f), further suggesting that DdBIC acts as a broad-spectrum inducer of GSDMC-mediated pyroptosis. Although many melanomas develop resistance to BRAF inhibitors,^25^ such as trametinib- and dabrafenib-resistant A375 melanoma cells (Supplementary Fig. 1g), DdBIC could induce pyroptosis in these resistant cells to an extent comparable to that in parental cells (Supplementary Fig. 1h), demonstrating that melanoma cells resistant to BRAF inhibitors remain sensitive to DdBIC-induced and GSDMC-dependent pyroptosis.

To investigate the target organelle, we synthesized a DdBIC probe (DdBIC-P) and a corresponding DdBIC negative probe (DdBIC-NP) (Fig. 1a). DdBIC-P could induce pyroptosis as effectively as DdBIC did (Fig. 1f). Furthermore, DdBIC-P reacted with rhodamine azide to generate a rhodamine conjugate (DdBIC-P-R), and under a confocal microscope, DdBIC-P-R localized in mitochondria but not in either the Golgi apparatus, lysosome, or endoplasmic reticulum (Supplementary Fig. 2a), accompanied by mitochondrial swelling (Fig. 1g). Consistently, DdBIC treatment induced a morphological transformation of mitochondria from elongated tubular networks to enlarged, hollow, circular structures, as demonstrated by immunostaining with an anti-Tom20 antibody that labels the mitochondrial outer membrane and the signal of HSP60, a mitochondrial matrix protein, became diffusely distributed throughout the enlarged mitochondria (Supplementary Fig. 2b). However, treatment with the mitochondrial fusion inhibitor MFI-8 or knockdown of MFN1 or MFN2, genes essential for mitochondrial fusion, did not attenuate DdBIC-induced mitochondrial swelling or pyroptosis (Supplementary Fig. 2c, d). These results indicate swelling rather than end-to-end fusion of mitochondria upon DdBIC stimulation. Analysis via transmission electron microscopy further demonstrated that DdBIC treatment induced marked mitochondrial swelling, characterized by dilation of the mitochondrial matrix and disruption of cristae architecture, and these alterations were substantially attenuated by cotreatment with hemin (Supplementary Fig. 2e). Importantly, DdBIC was more enriched in the mitochondrial fraction, as shown by mass spectrometry (Supplementary Fig. 2f). Collectively, these results suggest that DdBIC entry into mitochondria and alterations in mitochondrial function may be important events in pyroptotic induction.

We then investigated whether mitophagy is involved in DdBIC-induced mitochondrial swelling and pyroptosis. DdBIC treatment did not promote the colocalization of mitochondria with LC3, nor did it alter the levels of various mitochondrial proteins (Supplementary Fig. 2g). The pharmacological inhibitors of mitophagy, liensinine or Mdivi-1,^26^ did not impair DdBIC-induced mitochondrial swelling or pyroptosis (Supplementary Fig. 2h). Knocking down PINK1, a key mediator of mitophagy initiation,^27^ had no significant effect on DdBIC-induced cell swelling or pyroptotic cell death (Supplementary Fig. 2i). Together, these findings indicate that mitophagy is not functionally involved in DdBIC-induced pyroptosis. We also evaluated the effects of DdBIC on the mitochondrial membrane potential and ATP production, which demonstrated that DdBIC clearly inhibited both the mitochondrial membrane potential and ATP production in a dose-dependent manner (Supplementary Fig. 2j). Therefore, DdBIC-induced mitochondrial swelling is associated with the dissipation of the mitochondrial membrane potential and impaired ATP production.

Unexpectedly, DdBIC-induced pyroptosis was detected in culture media supplemented with 0.5%, but not 10%, fetal bovine serum (FBS) (Supplementary Fig. 2k), suggesting that some components in FBS might antagonize DdBIC-induced pyroptosis. Heme is an important component of FBS.^28^ The addition of hemin to the culture media containing 0.5% FBS diminished but the depletion of heme from the culture media with 10% FBS recovered DdBIC-induced pyroptosis (Fig. 1e and Supplementary Fig. 2k). Therefore, homeostasis of heme metabolism is involved in DdBIC-induced pyroptosis.

Given the critical role of heme metabolism homeostasis in maintaining mitochondrial function, we subsequently investigated the involvement of DdBIC in heme metabolism homeostasis. The initial and final steps of heme synthesis are carried out in mitochondria.^29^ DdBIC mainly downregulated the heme level in the mitochondria but not in the cytosol in a dose-dependent manner across the four cancer cell lines (Fig. 1h and Supplementary Fig. 2l). This phenomenon might be associated with the suppression of mitochondrial heme synthesis. Indeed, the expression of succinyl-CoA, the substrate for heme synthesis,^12^ was downregulated by DdBIC in a time-dependent manner (Fig. 1i). Dimethyl-α-ketoglutarate (DM-αKG, a cell-permeable analog of α-KG) can be metabolized by the α-ketoglutarate dehydrogenase complex to generate succinyl-CoA.^30^ DdBIC treatment led to a decrease in the mitochondrial heme level, while supplementation with DM-αKG reversed the DdBIC effect (Fig. 1j), accompanied by the attenuation of DdBIC-induced pyroptosis, as expected (Fig. 1k). SUCLG1 is indispensable for the conversion of succinyl-CoA to generate succinate.^30^ Knockdown of SUCLG1 abolished DdBIC-induced pyroptosis due to elevated levels of mitochondrial heme and succinyl-CoA (Fig. 1l, m). Although the inhibition of heme synthesis by succinylacetone (SA)^31^ alone was not sufficient to induce pyroptosis, it significantly reinforced DdBIC-induced pyroptosis (Supplementary Fig. 2m). Finally, the effects of hypoxia on the DdBIC-induced decreases in mito-heme and succinyl-CoA contents were excluded (Supplementary Fig. 2n). Together, these findings demonstrated that downregulating the level of succinyl-CoA in heme synthesis is critical for DdBIC-induced pyroptosis, revealing a novel link between heme metabolism homeostasis and GSDMC-dependent pyroptosis.

### DdBIC elevates mito-ROS levels by regulating the activity of the SDH complex

**Fig. 2:**
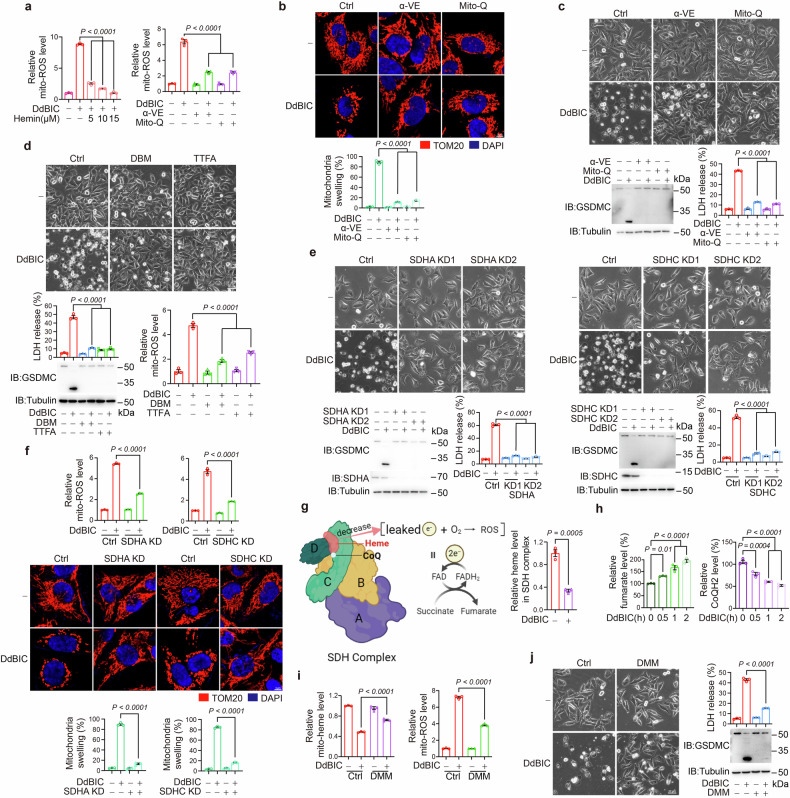
Mito-ROS are elevated by DdBIC through the regulation of the SDH complex in the ETC. Melanoma A375 cells were treated with DdBIC (20 μM) for 4 h to measure the levels of heme and mito-ROS and to observe the morphology of the mitochondria, for 8 h to assess pyroptotic features, unless otherwise specified. **a** Cells were cotreated with hemin, α-VE (25 μM) or Mito-Q (0.5 μM) and DdBIC as indicated to assay the levels of mito-ROS. **b**, **c** Cells were cotreated with α-VE or Mito-Q and DdBIC to observe mitochondrial morphology (**b**) and detect pyroptosis (**c**). Mitochondria and nuclei were shown after TOM20 and DAPI staining (**b**). **d** Cells were cotreated with DBM (1 mM) or TTFA (100 μM) and DdBIC to detect pyroptotic features and mito-ROS levels. **e**, **f** SDHA and SDHC were separately knocked down to detect pyroptosis (**e**) and the level of mito-ROS; mitochondrial morphology was shown, and mitochondrial swelling was quantified (**f**). **g** Left, model of the relationships among SDH, heme and mito-ROS. Right, cells were treated with DdBIC, and the heme level in the SDH complex was measured. **h** Cells were treated with DdBIC for the indicated times, and the levels of fumarate and CoQH_2_ were measured. **i**, **j** Cells were cotreated with DMM (7 mM) and DdBIC to assay the levels of mitochondrial heme and mito-ROS (**i**) and pyroptosis (**j**). Statistics: two-way ANOVA with Tukey’s test to **a**–**f**, **i** and **j**; one-way ANOVA with Tukey’s test to **h**; unpaired two-tailed Student’s *t* test to **g**. *P* values are shown

To investigate the involvement of heme metabolism in regulating pyroptosis, a fluorescent heme analog, ZnMP, was used to show heme localization in the mitochondria under DdBIC stimulation (Supplementary Fig. 3a). Heme plays an important role in the electron transport chain (ETC) in mitochondria to prevent electron leakage and inhibit the production of mitochondrial ROS (mito-ROS).^14^ DdBIC elevated mito-ROS levels in a dose-dependent manner in four cancer cell lines (Supplementary Fig. 3b), which was reversed by the addition of hemin to A375 cells (Fig. 2a, left). Mitoquinone mesylate (Mito-Q) and α-vitamin E (α-VE) are ROS inhibitors.^32^ When the cells were cotreated with these two reagents, the ability of DdBIC to increase the level of mito-ROS was clearly impaired (Fig. 2a, right), as were mitochondrial swelling (Fig. 2b) and pyroptosis (Fig. 2c). As heme deficiency is linked to the production of mito-ROS, cross-talk between heme homeostasis and the ETC may participate in regulating the level of mito-ROS. To determine which complex in the ETC is responsible for mito-ROS production, specific inhibitors were used. The addition of rotenone to Complex I, antimycin A to Complex III, NaN3 to Complex IV and oligomycin to Complex V resulted in no interference with pyroptosis or DdBIC-induced increases in mito-ROS levels (Supplementary Fig. 3c). Only the addition of a Complex II inhibitor, either DBM or TTFA, effectively inhibited pyroptosis and downregulated the level of DdBIC-induced mito-ROS (Fig. 2d). Similarly, knocking down components in other complexes, such as NDUFS4 for Complex I, RISP for Complex III, COX4I1 for Complex IV and ATP5PD for Complex V, still led to the appearance of DdBIC-associated pyroptotic morphologies (Supplementary Fig. 3d). Only the knockdown of SDHA and SDHC weakened the stability of Complex II (Supplementary Fig. 3e), prevented DdBIC-induced pyroptosis (Fig. 2e), downregulated mito-ROS levels, and attenuated mitochondrial swelling (Fig. 2f). Complex II (i.e., the SDH complex) contains a heme group^14^ (Fig. 2g, left), which is consistent with the fact that the addition of DdBIC was associated with a decreased heme level in the SDH complex (Fig. 2g, right). Moreover, replenishment of exogenous hemin prevented the decrease in heme levels in the SDH complex (Supplementary Fig. 3f), which further prevented the increase in mito-ROS (Fig. 2a, left). Clearly, heme deficiency caused an increase in mito-ROS, indicating that the SDH complex in the ETC was critical for pyroptotic induction.

The SDH complex catalyzes the conversion of succinate to fumarate to facilitate the production of electrons transferred to Coenzyme Q (CoQ) (Fig. 2g, left). The fact that the addition of DdBIC increased the fumarate level but decreased the CoQH_2_ level (Fig. 2h) indicated that the electron production from succinate with SDH activation was not further transferred to CoQ, which implied that electron leakage led to the production of mito-ROS. DMM (dimethyl malonate) is a competitive inhibitor of SDH and is an analog of succinic acid.^33^ When A375 cells were treated with DMM, there was neither a DdBIC-induced reduction in heme levels in the mitochondria nor an increase in mito-ROS levels (Fig. 2i) or pyroptosis (Fig. 2j). These data support the notion that activation of the SDH complex by DdBIC plays a crucial role in the induction of pyroptosis through disruption of heme homeostasis, thereby promoting electron leakage and subsequent mitochondrial ROS generation.

To validate the role of mito-ROS in GSDMC-dependent pyroptosis, we tested several stimuli known to promote mito-ROS accumulation, including Dox, β-lapachone, paclitaxel and vincristine sulfate, and found that none of these stimuli alone can trigger GSDMC-dependent pyroptosis (Supplementary Fig. 3g). Rotenone, which significantly upregulates mito-ROS, was not sufficient to induce GSDMC-dependent pyroptosis, although it increased DdBIC-induced pyroptosis (Supplementary Fig. 3h). This may be attributed to the relatively lower threshold value of mito-ROS in response to rotenone than in response to DdBIC (Supplementary Fig. 3i). Therefore, the level of mitochondrial ROS may serve as a critical determinant for the induction of pyroptosis.

### Protease OMA1 senses mito-ROS signals and induces OPA1 cleavage and release

**Fig. 3:**
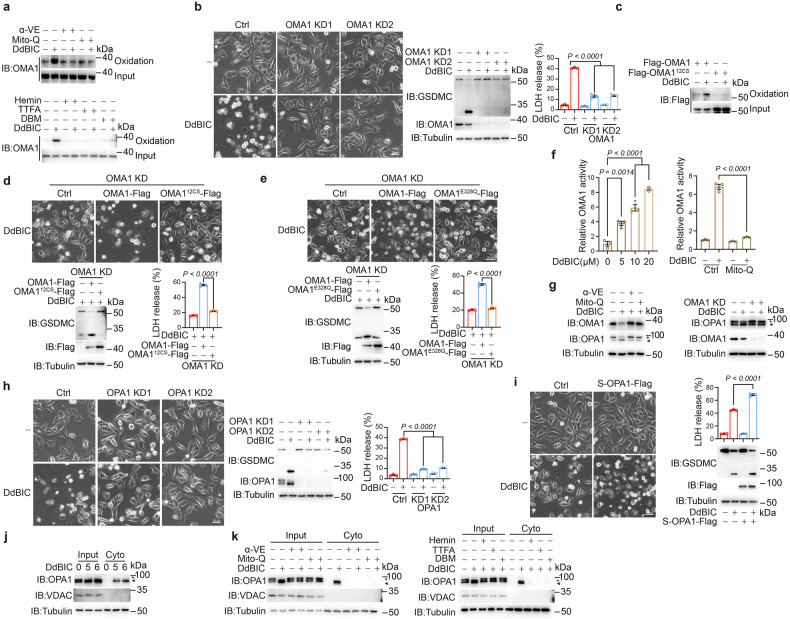
OMA1 senses DdBIC-induced mitochondrial ROS signals to cleave OPA1. Melanoma A375 cells were treated with DdBIC (20 μM) for 4 h to detect OMA1 oxidation and OPA1 cleavage, for 8 h to assess pyroptotic features, unless otherwise specified. **a** Cells were cotreated with α-VE, Mito-Q, hemin, DBM or TTFA and DdBIC as indicated to detect the oxidation of OMA1. **b** OMA1 was knocked down in cells to detect pyroptosis in response to DdBIC stimulation. **c**, **d** The 12 cysteines in OMA1 were all mutated to generate a mutant OMA1^12CS^. OMA1 oxidation was detected (**c**); after reintroducing either OMA1 or OMA1^12CS^ into OMA1-knockdown cells, pyroptosis was detected (**d**). **e** After reintroducing either OMA1 or OMA1^E328Q^ (the active site of OMA1) into OMA1-knockdown cells, pyroptosis was detected. **f** DdBIC elevates mito-ROS-driven OMA1 activity. OMA1 activity was measured via a fluorogenic substrate assay in A375 cells treated with DdBIC alone or cotreated with Mito-Q and DdBIC. **g** Cells were cotreated with α-VE or Mito-Q and DdBIC (left) or with OMA1 knockdown (right) to detect OMA1 activity and OPA1 cleavage. **h** OPA1 was knocked down in cells to detect pyroptosis. **i** S-OPA1 was overexpressed in cells to detect pyroptosis in the presence of DdBIC. **j**, **k** Cells were treated with DdBIC for the indicated times (**j**) with or without cotreatment with α-VE, Mito-Q, hemin, DBM or TTFA as indicated (**k**), and the cytosolic fractions were prepared to measure the levels of S-OPA1. Statistics: two-way ANOVA with Tukey’s test to **b**, **f**, **h** and **i**; one-way ANOVA with Tukey’s test to **d**–**f**. *P* values are shown

It is possible that mito-ROS function as a signal to trigger a cascade of molecular events in the mitochondria, leading to pyroptosis. After the detection of several related proteins through the resin-assisted capture of S-oxidated proteins, including ALAS1 (the rate-limiting enzyme of heme synthesis), SUCLG1/SUCLG2 (responsible for the catalytic conversion of succinyl-CoA to succinate), OGDH/DLST (involved in the catalytic conversion of α-KG to succinyl-CoA), and AFG3L2 and OMA1 (mitochondrial stress-related proteases) (Supplementary Fig. 4a), we found that only OMA1, which is located in the inner mitochondrial membrane, was oxidized with the addition of DdBIC, which could be attenuated by Mito-Q and α-VE (Fig. 3a, top and Supplementary Fig. 4a). Moreover, hemin, DBM, or TTFA interfered with DdBIC-induced OMA1 oxidation (Fig. 3a, bottom). Knockdown of either SDHA or SDHC suppressed DdBIC-induced OMA1 oxidation (Supplementary Fig. 4b). These results indicated that OMA1 sensed the DdBIC-induced mito-ROS signal derived from SDH complex activation through oxidation.

A knockdown of OMA1 attenuated DdBIC-induced pyroptosis (Fig. 3b) but did not alter the level of mito-ROS (Supplementary Fig. 4c, left), suggesting that OMA1 is downstream of the mito-ROS signal for pyroptotic induction. Mito-ROS likely attack vulnerable cysteine residues.^34^ There are 12 cysteine residues in OMA1. Mutation of all these cysteine residues to serine in OMA1 (OMA1^12CS^) completely abolished DdBIC-induced OMA1 oxidation (Fig. 3c) and pyroptosis (Fig. 3d). OMA1^E328Q^ is an enzymatically dead mutant.^35^ The oxidation level of OMA1^E328Q^ was comparable to that of OMA1 (Supplementary Fig. 4d), yet only the reintroduction of OMA1, not OMA1^E328Q^, restored DdBIC-induced pyroptosis in OMA1-knockdown cells (Fig. 3e), suggesting that OMA1 enzymatic activity is required for pyroptotic induction.

DdBIC induced OMA1 activation in a dose-dependent manner, and this effect was abolished by scavenging mitochondrial ROS with mito-Q (Fig. 3f), suggesting the requirement of mito-ROS for DdBIC-enhanced OMA1 activation. The decreased intensity of OMA1 (approximately 40 kDa) observed via Western blotting also indicates the activation of OMA1.^36^ DdBIC-enhanced activation of OMA1 resulted in cleavage of its downstream target protein OPA1, leading to increased production of the short isoform (S-OPA1, indicated by the arrow) and reduced formation of the long isoform (L-OPA1, indicated by the asterisk) (Fig. 3g, left). The knockdown of OMA1 was coupled with the failure of DdBIC to induce OPA1 cleavage (Fig. 3g, right). Treatment with Mito-Q or α-VE impaired DdBIC-induced OMA1 activity and decreased S-OPA1 levels (Fig. 3g, left). In OMA1-knockdown cells, reintroducing OMA1, but not OMA1^E328Q^ or OMA1^12CS^, resulted in an increase in S-OPA1 levels upon DdBIC stimulation (Supplementary Fig. 4e, f). Treatment with hemin or knockdown of either SDHA or SDHC attenuated DdBIC-enhanced OMA1 activity, thereby impairing OPA1 cleavage (Supplementary Fig. 4g). These results extended a signal axis from the heme-regulated ETC to mito-ROS-mediated OMA1 activation and OPA1 cleavage in the mitochondria.

The knockdown of OPA1 effectively rescued A375 cells from DdBIC-induced pyroptosis (Fig. 3h) but did not alter mito-ROS levels (Supplementary Fig. 4c, right). The overexpression of S-OPA1 effectively enhanced DdBIC-induced GSDMC cleavage, leading to intensive pyroptosis (Fig. 3i). The sequence of residues 194-195 in OPA1 is critical for OMA1 to cleave OPA1.^37^ When OPA1^Δ194-195^ was transfected into OPA1-knockdown A375 cells, pyroptosis was not detected (Supplementary Fig. 4h). These results demonstrated that OPA1 cleavage was essential for pyroptotic induction upon DdBIC stimulation. Importantly, S-OPA1, but not L-OPA1, was released from the mitochondria to the cytosol after DdBIC stimulation (Fig. 3j), which was abolished by α-VE, Mito-Q, hemin, TTFA, or DBM (Fig. 3k). These results demonstrated that OMA1-mediated OPA1 cleavage triggered by increased mito-ROS was crucial for DdBIC-induced pyroptosis and that the release of S-OPA1 into the cytosol might aggravate pyroptosis. Although rotenone treatment alone elevated mitochondrial ROS, it did not promote the release of S-OPA1 from the mitochondria into the cytoplasm (Supplementary Fig. 4i), likely due to the relatively limited increase in mito-ROS compared with that induced by DdBIC (Supplementary Fig. 3i). The threshold value of mito-ROS appears to determine the release of S-OPA1 from mitochondria, which is consistent with the notion that a higher level of mitochondrial ROS may lead to mitochondrial rupture.^38^

Mitochondrial swelling and subsequent rupture of the outer mitochondrial membrane are closely associated with the mitochondrial permeability transition (mPT).^39,40^ Indeed, DdBIC induced mPT in a dose-dependent manner (Supplementary Fig. 4j). This effect was associated with the opening of the mitochondrial permeability transition pore (mPTP), as pharmacological inhibition of the mPTP by cyclosporin A (CsA) or knockdown of ANT1 and CypD, key components of the mPTP, markedly attenuated DdBIC-induced mPT (Supplementary Fig. 4k), leading to suppressed DdBIC-induced mitochondrial swelling (Supplementary Fig. 4l), the release of S-OPA1 into the cytoplasm (Supplementary Fig. 4m), and pyroptotic induction (Supplementary Fig. 4n). In conclusion, mPTP opening is essential for DdBIC-induced mitochondrial swelling and the subsequent release of S-OPA1.

### Cytosolic OPA1 causes ISR by inducing PERK dimerization and phosphorylation

**Fig. 4:**
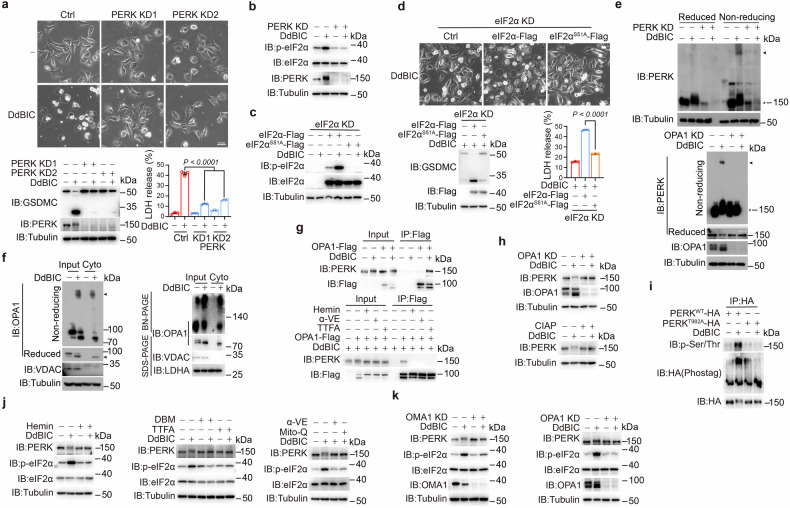
DdBIC activates the PERK-mediated ISR pathway to induce pyroptosis. Melanoma A375 cells were treated with DdBIC (20 μM) for 5 h to detect the dimerization of PERK and phosphorylation of PERK and eIF2α for 8 h to assess pyroptotic features, unless otherwise specified. **a** PERK was knocked down in cells, after which pyroptosis was detected. **b** PERK was knocked down to detect the phosphorylation of eIF2α. **c**, **d** eIF2α and eIF2α^S51A^ were reintroduced into eIF2α-knockdown cells to detect the phosphorylation of eIF2α (**c**) and pyroptosis (**d**). **e** PERK or OPA1 was knocked down first in cells, the dimerization of PERK was determined. **f** A cytosolic fraction was prepared to detect DdBIC-induced OPA1 dimers through nonreducing SDS-PAGE and BN-PAGE. **g** In OPA1-overexpressing cells, DdBIC-induced interactions between OPA1 and endogenous PERK were detected in the presence of hemin, α-VE or TTFA, as indicated. **h** In OPA1-knockdown cells, PERK phosphorylation (upshift band) was detected (top), which was abolished by incubation with CIAP (bottom). **i** Cells expressing PERK^WT^-HA or a phosphorylation-deficient PERK^T982A^-HA mutant were treated with DdBIC. PERK was immunoprecipitated (IP) via an anti-HA antibody, and phosphorylation levels were assessed via a Phos-tag assay and immunoblotting with an anti-panphospho-Ser/Thr antibody. **j** Cells were cotreated with hemin, DBM, TTFA, α-VE or Mito-Q and DdBIC as indicated to determine the phosphorylation levels of PERK and eIF2α. **k** In OMA1- or OPA1-knockdown cells, the phosphorylation levels of PERK and eIF2α were determined. Statistics: two-way ANOVA with Tukey’s test to **a**; one-way ANOVA with Tukey’s test to **d**. *P* values are shown

The outcome of DdBIC-associated OPA1 release into the cytosol was further investigated. RNA-seq analysis via gene set enrichment analysis (GSEA) revealed that DdBIC treatment positively regulated integrated stress response (ISR) signaling and PERK-regulated gene expression (Supplementary Fig. 5a, top), suggesting that DdBIC might activate the PERK-mediated ISR. Several experiments have been carried out to substantiate this possibility. First, knockdown of PERK or cotreatment with the ISR inhibitor ISRIB (an integrated stress response inhibitor) reduced DdBIC-induced pyroptosis (Fig. 4a and Supplementary Fig. 5b). In PERK-knockdown cells, only reintroduction of PERK^WT^, but not the kinase-dead mutant PERK^T982A^, restored DdBIC-induced pyroptotic morphology (Supplementary Fig. 5c). Phosphorylation of eIF2α is a characteristic of ISR activation.^35,41^ DdBIC stimulation indeed phosphorylated eIF2α, yet no phosphorylation of eIF2α occurred after PERK was knocked down (Fig. 4b). PERK phosphorylates eIF2α at residue Ser51.^41^ When this residue was mutated (eIF2α^S51A^), DdBIC could no longer phosphorylate eIF2α (Fig. 4c), resulting in the failure to induce pyroptosis (Fig. 4d). Therefore, PERK caused DdBIC-induced ISR activation and subsequent pyroptosis.

Second, DdBIC stimulation clearly promoted the homodimerization of endogenous PERK, which could be diminished by the knockdown of OPA1 (Fig. 4e) or cotreatment with Mito-Q, α-VE and hemin (Supplementary Fig. 5d). OPA1 belongs to the dynamin superfamily and has a GTPase domain for dimerization.^42^ We detected the dimerization of OPA1 in both total cell lysates and cytoplasmic fractions upon DdBIC stimulation, as detected by nonreducing SDS-PAGE and BN-PAGE (Fig. 4f). As only S-OPA1 was detected in the cytoplasm, S-OPA1 likely dimerized upon DdBIC treatment. PERK interacted with OPA1 only under DdBIC treatment, which could be inhibited by TTFA, α-VE and hemin (Fig. 4g). When the two critical sites (C853 and C856) for OPA1 homodimerization^42,43^ were both mutated to serine (OPA1^2CS^), the formation of the homodimeric OPA1 was greatly diminished in the cytoplasm (Supplementary Fig. 5e, left). Moreover, in OPA1-knockdown cells, reintroducing OPA1 but not OPA1^2CS^ induced PERK dimerization (Supplementary Fig. 5e, right), demonstrating that the dimerization of OPA1 was a prerequisite for PERK dimerization. Together, these findings suggest that the formation of the PERK homodimer was associated with S-OPA1 dimerization in the cytoplasm under DdBIC stimulation.

PERK dimerization leads to its autophosphorylation.^44^ We also found that DdBIC could induce an upward band shift of PERK via Western blotting, which was abolished by treatment with calf intestinal alkaline phosphatase (CIAP) in cell lysates (Fig. 4h, bottom) or a kinase-dead PERK mutant (PERK^K618R^) (Supplementary Fig. 5f, top), reflecting possible PERK phosphorylation. This DdBIC-induced phosphorylation of PERK was further confirmed via an anti-phospho-Ser/Thr antibody and a Phostag assay (Fig. 4i). The knockdown of OPA1 or the inhibition of S-OPA1 release through the suppression of mPTP opening abolished DdBIC-induced PERK phosphorylation (Fig. 4h, top, Supplementary Fig. 5g). When the self-phosphorylation site (PERK^T982A^) was mutated, DdBIC promoted the dimerization of PERK^T982A^ (Supplementary Fig. 5h) but failed to induce the phosphorylation of PERK^T982A^ (Supplementary Fig. 5f, bottom). Together, these findings suggest that homodimerization may be an indicator of PERK activation for the activation of the ISR. In addition, forced dimerization of S-OPA1 by the fusion of a hormone-binding domain (HBD*) and treatment with 4-hydroxytamoxifen (4-OHT) led to the activation of PERK and the phosphorylation of eIF2α, as well as GSDMC cleavage and pyroptosis (Supplementary Fig. 5i), further supporting the important role of cytosolic OPA1 in the activation of PERK activity.

Finally, we investigated the correlation between the ISR and a series of factors upstream of pyroptosis induction. The addition of hemin or elevation of the succinyl-CoA level for de novo synthesis of heme by knocking down SUCLG1 significantly attenuated DdBIC-induced PERK activity and ISR activation (Supplementary Fig. 5a, bottom, Fig. 4j, left and Supplementary Fig. 5j), suggesting that there was indeed a signaling pathway linking the mitochondrial heme level to ISR activation. Cotreatment of cells with either DBM or TTFA or knockdown of SDHA and SDHC interfered with the function of complex II in the ETC, or Mito-Q and α-VE, which inhibited mito-ROS, abolished DdBIC-induced phosphorylation of PERK and eIF2α (Fig. 4j and Supplementary Fig. 5k). PERK-mediated ISR activation was also associated with OMA1-induced OPA1 cleavage, as the knockdown of either OMA1 or OPA1 abolished DdBIC-induced PERK and eIF2α phosphorylation (Fig. 4k). In OMA1-knockdown cells, the reintroduction of OMA1, but not OMA1^E328Q^ or OMA1^12CS^, facilitated DdBIC-induced PERK and eIF2α phosphorylation (Supplementary Fig. 5l). Moreover, in OPA1-knockdown cells, S-OPA1 but not the uncleavable OPA1 mutant (OPA1^Δ194-195^) rescued DdBIC-induced PERK dimerization as well as PERK and eIF2α phosphorylation (Supplementary Fig. 5m). Activation of the OMA1-OPA1-ISR signaling axis by DdBIC was also demonstrated to occur in a dose-dependent manner in four tumor cell lines (Supplementary Fig. 5n). Although PERK activation is associated with CHOP-dependent apoptosis,^45^ the knockdown of CHOP did not affect DdBIC-induced LDH release or pyroptotic morphology (Supplementary Fig. 5o). Together, these findings suggest that DdBIC-induced mito-ROS elevation originated from the regulation of both SDH complex activity and heme synthesis, leading to pyroptotic induction along the OMA1-OPA1-PERK-eIF2α signaling axis.

### ISR-activated granzyme B is a novel protease that cleaves GSDMC

**Fig. 5:**
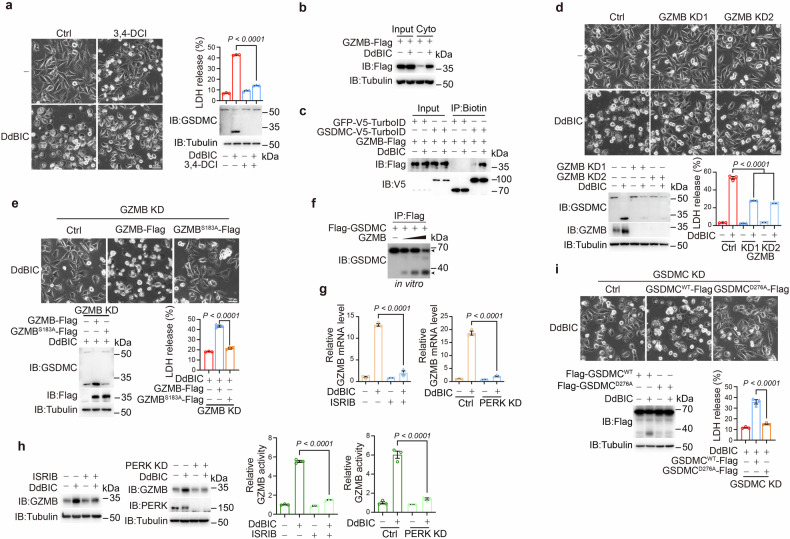
Granzyme B cleaves GSDMC in response to DdBIC stimulation. Melanoma A375 cells were treated with DdBIC (20 μM) for 6 h to determine the level and activity of granzyme B (GZMB), for 7 h to determine the interaction between GZMB and GSDMC, or for 8 h to assess pyroptotic features, unless otherwise specified. **a** Cells were cotreated with 3,4-DCI (10 μM) for 8 h to detect pyroptosis. **b** GZMB was transfected into cells, and the GZMB level in the cytosolic fraction was detected in response to DdBIC stimulation. **c** GZMB and GSDMC were overexpressed for the TurboID-based assessment of the interaction between GZMB and GSDMC. **d** GZMB was knocked down to assay pyroptosis. **e** GZMB and GZMB^S183A^ were transfected into GZMB-knockdown cells to detect pyroptosis. **f** The cleavage of GSDMC by recombinant GZMB was detected in vitro. **g**, **h** Cells were cotreated with ISRIB and DdBIC or with PERK knockdown to determine the mRNA (**g**) or protein (**h**) levels of GZMB and GZMB activity. **i** GSDMC and GSDMC^D276A^ were reintroduced into GSDMC-knockdown cells to detect pyroptosis. Statistics: two-way ANOVA with Tukey’s test to **a**, **d**, **g** and **h**; one-way ANOVA with Tukey’s test to **e** and **i**. *P* values are shown

Although caspase-8 and caspase-6 have been reported to be proteases for GSDMC cleavage,^5,6,46^ their knockdown did not affect DdBIC-induced GSDMC cleavage or pyroptosis (Supplementary Fig. 6a, b). Since the pan-caspase inhibitor Z-VAD did not inhibit DdBIC-induced GSDMC cleavage or pyroptotic cell death (Supplementary Fig. 6c), a protease other than caspases might be responsible for GSDMC cleavage. After screening a set of protease inhibitors, 3,4-dichloroisocoumarin (3,4-DCI) was the only one that influenced DdBIC-induced GSDMC cleavage and pyroptosis (Fig. 5a and Supplementary Fig. 6d). 3,4-DCI is an inhibitor of granzyme proteases.^7^ The granzyme (GZM) family has several members, including GZM-A, GZM-B, GZM-K, GZM-H, and GZM-M. Among the granzymes, only GZMB is reported to be expressed in some cancer cell lines.^47,48^ DdBIC treatment increased GZMB expression in a dose-dependent manner in the four cancer cell lines (Supplementary Fig. 6e). DdBIC promoted the accumulation of GZMB in the cytosol (Fig. 5b), which facilitated the movement of GZMB toward GSDMC (Fig. 5c), setting the conditions for GZMB to cleave GSDMC. Moreover, knockdown of GZMB effectively abolished DdBIC-induced cleavage of GSDMC and pyroptosis (Fig. 5d), whereas overexpression of a GZMB mutant without protease activity (GZMB^S183A^)^49^ in GZMB-knockdown cells not only attenuated DdBIC-induced cleavage of GSDMC but also abolished pyroptosis (Fig. 5e). Consistently, Flag-GSDMC extracted from the expressing cells was cleaved by incubation with recombinant GZMB in an in vitro assay (Fig. 5f). These results clearly revealed a novel function by which granzyme B cleaves GSDMC in tumor cells.

Since neither DdBIC-induced OPA1 cleavage nor DdBIC-associated PERK phosphorylation was impaired by 3,4-DCI treatment or by GZMB knockdown in A375 cells (Supplementary Fig. 6f), DdBIC induction of GZMB may be associated with ISR activation. Indeed, DdBIC effectively promoted GZMB mRNA expression, and both ISRIB cotreatment or PERK knockdown downregulated DdBIC-induced GZMB mRNA expression (Fig. 5g), with a subsequent decrease in the GZMB protein level and activity (Fig. 5h). The DdBIC enhancement of GZMB mRNA and protein expression, as well as its activity, was the result of PERK-mediated ISR activation.

To evaluate whether the activation of PERK could mimic upstream signals to induce pyroptosis, we treated cells with various agents known to activate PERK. We found that treatment with thapsigargin or tunicamycin, two ER stress inducers with the capacity to activate PERK,^35,50^ did not lead to detectable GZMB expression or pyroptosis (Supplementary Fig. 6g). In contrast, treatment with MK-28 or CCT-020312, two selective PERK activators,^51,52^ significantly increased GZMB expression and pyroptotic induction (Supplementary Fig. 6h). Immunoblot analysis of PERK activation (phosphorylation of PERK and eIF2α) revealed that treatment with MK-28, CCT-020312, or DdBIC resulted in the activation of PERK, whereas thapsigargin or tunicamycin treatment resulted in less phosphorylation (Supplementary Fig. 6i), suggesting that the activation of PERK must reach a certain threshold before GZMB-mediated and GSDMC-dependent pyroptosis occurs.

It has been reported that granzyme B typically localizes to lysosome-related organelles (LROs) in immune cells, which are positive for LAMP1 staining.^53^ In A375 melanoma cells, the colocalization of granzyme B with LAMP1 has been observed.^47^ Similarly, under control conditions, we found that granzyme B localized to LAMP1-positive foci in A375 cells, whereas DdBIC treatment not only induced granzyme B expression (Supplementary Fig. 6e) but also caused its diffuse distribution throughout the cytoplasm, resulting in partial colocalization with GSDMC in the cytoplasm (Supplementary Fig. 6j). Lysosomal membrane permeabilization (LMP) has been implicated in the release of granzyme B into the cytoplasm.^53^ Indeed, DdBIC treatment significantly induced the dissipation of acridine orange (AO) red fluorescence and the formation of galectin-3 puncta (Supplementary Fig. 6k), two well-established markers of LMP.^18,54^ Therefore, DdBIC-induced LMP likely facilitates the release of granzyme B into the cytoplasm, where it interacts with and cleaves GSDMC to induce pyroptosis.

The critical sequence in GSDMC cleaved by GZMB was analyzed. Docking experiments indicated that the long hinge region in GSDMC could be fitted into the substrate binding cleft in GZMB (Supplementary Fig. 6l). An analysis of the amino acid sequence of GSDMC suggested that the deletion of a sequence containing residues 260-280 (GSDMC^Δ260-280^) could abolish its cleavage (Supplementary Fig. 6m, left). It has been reported that granzymes are cleaved at the site containing an Asp residue.^8,48^ On the basis of the molecular weights of the cleaved GSDMC fragments, the cleavage site should be one of the five Asp residues in the region containing residues 200-300. After these five Asp residues were separately mutated, GSDMC^D276A^ was the one that resisted GZMB cleavage either in an in vitro assay or in GSDMC-knockdown cells transfected with the same mutation (Supplementary Fig. 6m, right and n). Failure of DdBIC-induced cleavage of GSDMC^D276A^ in transfected cells also abolished pyroptosis (Fig. 5i). To further evaluate the pore-forming activity of GSDMC^1-276^, both the full-length GSDMC and its N-terminal fragment containing residues 1-276 were fused to a hormone-binding domain (HBD*)-HA tag, which forced dimerization upon treatment with 4-hydroxytamoxifen (4-OHT).^6^ Treatment with 4-OHT triggered pyroptosis and LDH release in A375 cells expressing GSDMC^1-276^-HBD-HA but not in A375 cells expressing GSDMC^WT^-HBD-HA (Supplementary Fig. 6o). Similar pyroptotic induction was also demonstrated in HEK293T cells expressing GSDMC^1-276^-Flag (Supplementary Fig. 6p). D276 is located in the hinge region adjacent to the N-terminal domain, whereas Asp365 resides within the C-terminal domain of GSDMC. Upon cleavage of GSDMC at D276, the resulting 1-276 fragment lacks the entire C-terminal domain, whereas cleavage at Asp365 results in a portion of GSDMC-CT remaining attached to the N-terminal domain (Supplementary Fig. 6q, top). This difference may affect the pore-forming activity of GSDMC-NT, as evidenced by the observation that, compared with GSDMC^1-365^, CSDMC^1-276^ seemed to be more potent in the induction of pyroptosis (Supplementary Fig. 6q, bottom). Overall, GSDMC^1-276^ alone was sufficient to induce pyroptosis even without DdBIC stimulation, and D276 was the critical residue for GZMB cleavage.

### DdBIC is an agonist that directly binds to the orphan nuclear receptor Nur77

**Fig. 6:**
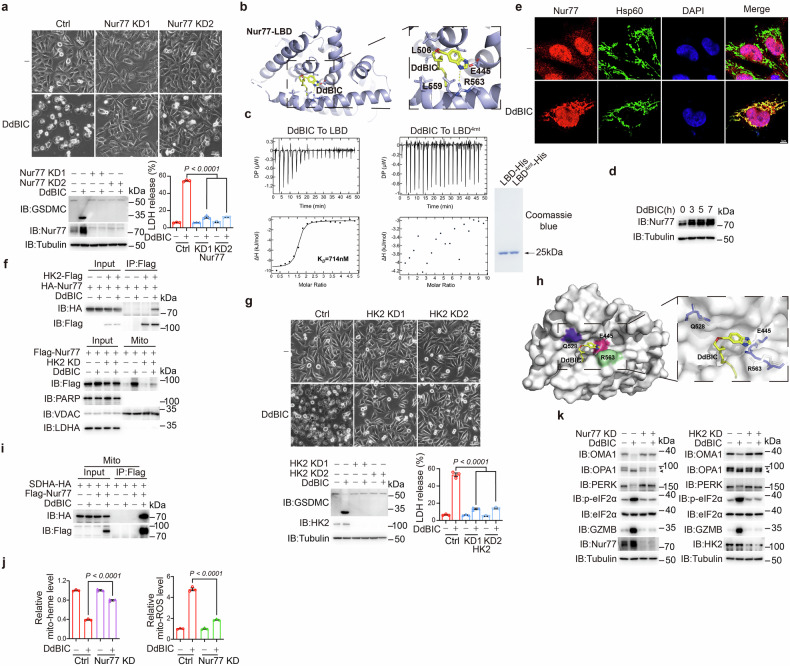
Nur77 is the target of DdBIC. Melanoma A375 cells were treated with DdBIC (20 μM) for 0.5 h to display Nur77 mitochondrial localization and its interaction with other proteins, for 4 h to measure the levels of heme and mito-ROS, for 8 h to assess pyroptotic features, unless otherwise specified. **a** Nur77 was knocked down to detect pyroptosis. **b** DdBIC bound to a pocket formed by helices 5, 8, and 10 of the LBD. The LBD is shown as a light blue cartoon, amino acids near DdBIC are shown as sticks, and hydrogen bonds are shown as yellow dashed lines. **c** Measuring the binding affinity of DdBIC with either the LBD or LBD^4mt^ via ITC. The titration data were analyzed via the program MicroCal PEAQ-ITC Analysis Software and fitted with a one-site binding model. The levels of LBD or LBD^4mt^ were indicated (right). **d** Cells were treated with DdBIC for the indicated times, and the expression levels of Nur77 were detected. **e** Cells were treated with DdBIC to observe Nur77 localization. Nur77 and mitochondria were indicated with their corresponding antibodies, and nuclei were shown by DAPI staining. **f** Cells were transfected with HK2 and Nur77, and their interaction was determined (top). Nur77 was transfected into HK2-knockdown cells, and the mitochondrial localization of Nur77 was determined (bottom). **g** HK2 was knocked down to detect pyroptosis. **h** Binding surface of the LBD for HK2. The binding of DdBIC to the LBD induces conformational changes in residues Q528, R563 and E445 (white sticks) from those in the apo structure (purple sticks). **i** Nur77 and SDHA were overexpressed, and the mitochondrial fraction was prepared to determine the interaction between Nur77 and SDHA. **j** The levels of mitochondrial heme and mito-ROS were determined in Nur77-knockdown cells. **k** Nur77 and HK2 were separately knocked down in cells, and OMA1 activity, OPA1 cleavage, phosphorylation of PERK and eIF2α, and GZMB expression levels were analyzed. Statistics: two-way ANOVA with Tukey’s test to **a**, **g** and **j**. *P* values are shown

A DdBIC probe (DdBIC-P), but not a nonfunctional DdBIC analog (DdBIC-negative probe, DdBIC-NP), could induce pyroptosis as DdBIC did (Fig. 1f). When DdBIC-P was reacted with biotin azide to generate a DdBIC-P/biotin conjugate (DdBIC-P-B), a distinct band was detected via SDS-PAGE electrophoresis (Supplementary Fig. 7a, left). Mass spectrometry revealed four candidates (Supplementary Fig. 7a, right). Knocking down each of these candidates revealed that the nuclear receptor Nur77 (Fig. 6a), but not any of the other three proteins, participated in DdBIC-induced pyroptosis (Supplementary Fig. 7b). Knockdown of Nur77 also impaired DdBIC-induced cell death in the other three cancer cell lines (Supplementary Fig. 7c). Furthermore, Nur77 was the endogenous target to which DdBIC-P bound. In contrast, no detectable DdBIC-NP could bind Nur77 (Supplementary Fig. 7d). Nur77 is an orphan nuclear receptor, and its first agonist was identified by our group. To elucidate the binding mode, the complex structure between DdBIC and Nur77’s ligand binding domain (LBD) was elucidated by X-ray crystallography to a resolution of 2.57 Å (Supplementary Fig. 7e, top and Supplementary Table 1). The asymmetric unit of the crystal contained two LBD molecules in different conformations, similar to our previously reported structures,^18^ and only one of the LBD molecules (Mol I) was bound to a DdBIC molecule. The “tail” of DdBIC bound the LBD in a peg-in-hole fashion, and the “head” of DdBIC was in a conformation that rotated approximately 90° around its flexible “neck” away from the conformation of a previously identified compound, THPN, which performs a distinct function to induce autophagic cell death^18^ (Supplementary Fig. 7e, bottom and Supplementary Fig. 7f). Residues E445, L506 and L559, and R563 seemed important for DdBIC binding (Fig. 6b), and a mutation of these four residues in the LBD (LBD^E445A/L506A/L559A/R563A^ or LBD^4mt^) abolished DdBIC binding, as shown by isothermal titration calorimetry (ITC) measurements (Fig. 6c). These results demonstrated that the LBD of Nur77 was the direct target of DdBIC and four residues (E445, L506, L559, and R563) were critical for DdBIC binding.

DdBIC increased Nur77 expression in a dose-dependent manner (Supplementary Fig. 7g) and a time-dependent manner (Fig. 6d). DdBIC-induced Nur77 was localized in the mitochondria (Fig. 6e), although Nur77 has no sequence for mitochondrial targeting. Nix, which reportedly facilitates Nur77 targeting to mitochondria,^18,55^ was not influenced by DdBIC treatment, as DdBIC-bound Nur77 did not interact with Nix (Supplementary Fig. 7h). In addition, Nix knockdown did not affect Nur77 mitochondrial localization (Supplementary Fig. 7i). On the basis of the hypothesis that DdBIC-induced mitochondrial targeting of Nur77 may depend on its interaction with chaperone proteins localized at the mitochondrial outer membrane, an AlphaFold 3-based screening was conducted to analyze the Nur77-DdBIC cocrystal structure in conjunction with mitochondrial outer membrane proteins exhibiting at least 50% structural coverage available in UniProt. The top candidate proteins were subsequently subjected to individual knockdown experiments to evaluate their roles in DdBIC-induced mitochondrial translocation of Nur77. Among these candidates, HK2 depletion significantly impaired Nur77 mitochondrial localization following DdBIC stimulation (Supplementary Fig. 7j). Furthermore, HK2 could chaperone Nur77 to target mitochondria in the presence of DdBIC (Fig. 6f). Knockdown of HK2 resulted in no Nur77 translocation to the mitochondria and failure of pyroptotic induction even in the presence of DdBIC (Fig. 6f, g). The 16 N-terminal residues constitute the HK2 mitochondria-targeting sequence.^56^ Deletion of this sequence resulted in the cytosolic diffusion of HK2^Δ1-16^ (Supplementary Fig. 7k), accompanied by the loss of Nur77-dependent mitochondria targeting in response to DdBIC (Supplementary Fig. 7l). In Nur77-knockdown cells, reintroducing Nur77^4mt^, as well as treatment with DdBIC, resulted in neither its interaction with HK2 nor its mitochondrial localization (Supplementary Fig. 7m, n). Thus, DdBIC binding to Nur77 is the basis for the interaction between Nur77 and HK2, and HK2 promotes Nur77 translocation to the mitochondria. As expected, mPTP opening is not required for the mitochondrial localization of Nur77, as treatment with CsA or ANT1 knockdown did not affect the protein levels of Nur77 in the mitochondrial fraction (Supplementary Fig. 7o), further suggesting that Nur77 acts upstream to regulate the mPTP.

Docking experiments revealed that HK2 bound to the LBD at a site close to the DdBIC binding pocket (Supplementary Fig. 7p). It is possible that DdBIC binding to the Nur77 LBD produces an amenable surface to facilitate the interaction between Nur77 and HK2. In the crystal structure, the conformations of residues Q528, E445 and R563 in the putative interacting surface were perturbed with the binding of DdBIC (Fig. 6h). The transfection of a Nur77^E445A/Q528A/R563A^ mutant (i.e., Nur77^3mt^) in Nur77 knockdown cells abolished the interaction with HK2 (Supplementary Fig. 7m). As a result, Nur77^3mt^ could no longer translocate to the mitochondria even in the presence of DdBIC (Supplementary Fig. 7n). These results support the notion that the binding of DdBIC in a specific Nur77 conformation is essential for the formation of an amenable surface for the interaction of Nur77 with HK2.

The possibility of Nur77 translocation to the outer mitochondrial membrane was excluded, as protease K can digest the outer mitochondrial membrane protein Tom20 but not Nur77 in mitochondria (Supplementary Fig. 7q). Nur77 strongly interacted with SDHA, which was located at the inner mitochondrial membrane, in the presence of DdBIC (Fig. 6i). This further triggered downstream events, including a reduction in heme, elevation of mito-ROS levels (Fig. 6j), activation of OMA1, cleavage of OPA1, and ISR activation, as indicated by PERK and eIF2α phosphorylation, as well as increased GZMB protein levels in Nur77- and HK2-dependent manners (Fig. 6k). Mutations at either the DdBIC binding sites or the interacting sites with HK2 (Nur77^4mt^ and Nur77^3mt^) impaired DdBIC-induced mitochondrial swelling (Supplementary Fig. 7r), mito-heme reduction and mito-ROS level elevation (Supplementary Fig. 7s), OMA1 activation, OPA1 cleavage, and PERK/eIF2α phosphorylation (Supplementary Fig. 7t), as well as consequent pyroptosis (Supplementary Fig. 7u). When all key residues involved in DdBIC binding (E445, L506, L559, and R563) and HK2 binding (E445, Q528, and R563) to Nur77 were mutated (E445A, L506A, Q528A, L559A, and R563A; designated Nur77^5mt^), DdBIC was no longer able to activate the OMA1-OPA1-PERK-eIF2α signaling axis (Supplementary Fig. 7t). Similar results were obtained in HK2^Δ1-16^-expressing cells (Supplementary Fig. 7v, w), which was also expected to contribute to pyroptotic inhibition (Supplementary Fig. 7x). Together, these results demonstrated that the orphan nuclear receptor Nur77 participated in pyroptotic induction through the SDH complex-regulated heme/mito-ROS/ISR pathway, with DdBIC functioning as a novel agonist and HK2 playing a unique role in directing Nur77 into the mitochondria.

### Nur77 and GSDMC mediate pyroptosis through regulating heme levels in mouse models

**Fig. 7:**
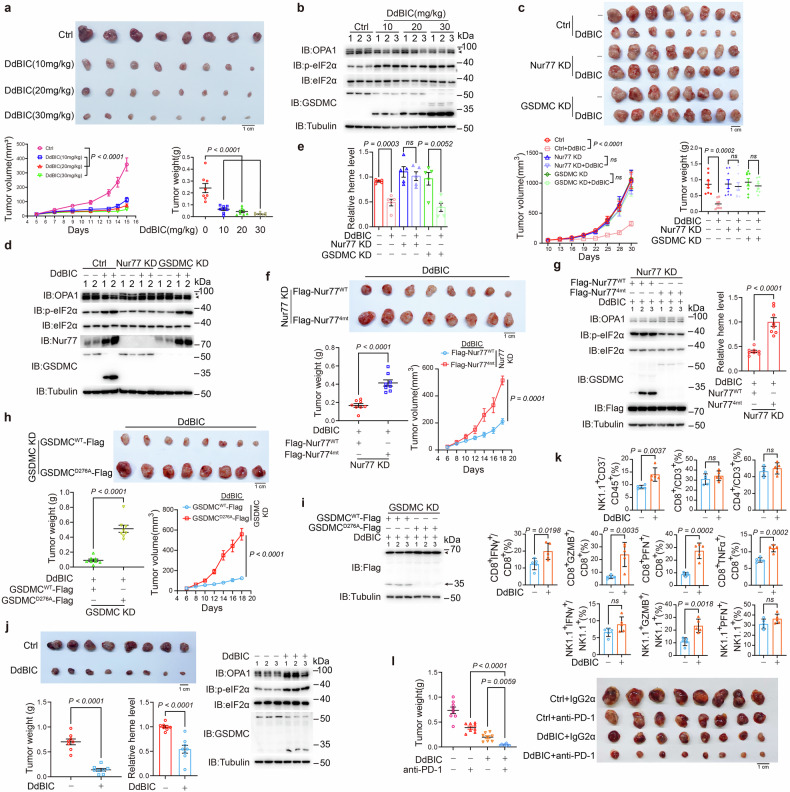
DdBIC inhibits tumor growth by inducing Nur77-mediated and GSDMC-induced pyroptosis in mouse models. For the xenograft model, melanoma A375 cells were injected subcutaneously into the posterior flanks of nude mice (n = 8). When the tumors reached a diameter of approximately 3–4 mm, the mice were intraperitoneally administered DdBIC (20 mg/kg) every other day for 2 weeks and then sacrificed. **a** Comparison of the sizes, volumes and weights of xenograft tumors treated with different concentrations of DdBIC. **b** Detection of the cleavage of OPA1 and GSDMC and ISR activation (represented by eIF2α phosphorylation) in xenograft tumor samples from the same tumors as in (**a**). **c** Nur77 or GSDMC was separately knocked down in A375 cells. The mice were treated with DdBIC. Images of xenograft tumors and the tumor volumes and weights were shown. **d**, **e** Phosphorylation of eIF2α and heme levels were detected in the same tumors as in (**c**). **f** Nur77 was knocked down first in A375 cells, and Nur77^WT^ and Nur77^4mt^ were reintroduced into the cells. The mice were treated with DdBIC. Images of xenograft tumors and the tumor volumes and weights were shown. **g** The cleavage of OPA1 and GSDMC, the phosphorylation of eIF2α and the heme level were measured in the same tumors as in (**f**). **h**, **i** GSDMC was knocked down first in A375 cells, and GSDMC^WT^ and GSDMC^D276A^ were reintroduced into the cells. The mice were treated with DdBIC. Images of xenograft tumors and the tumor volumes and weights were shown (**h**). The cleavage of GSDMC in the same tumors as in (**h**) was also detected (**i**). **j** C57BL/6J mice bearing murine melanoma B16 cell-derived allografts were treated intraperitoneally with DdBIC for 2 weeks. Tumors and tumor weights are indicated (n = 8). The heme level, cleavage of OPA1 and GSDMC, and ISR activation were indicated. **k** B16 cell-derived allograft tumors were collected after DdBIC administration for 24 h, and the proportion and activation status of immune cells within the TME (n = 5) were analyzed. **l** C57BL/6J mice bearing subcutaneous B16 melanoma cells were treated with i.p. injections of DdBIC and anti-PD-1 antibody every other day. Representative images and weights of tumors at the endpoint were shown (n = 8 mice per group). Statistics: two-way ANOVA with Tukey’s test to **c**, **e** and **l**; one-way ANOVA with Tukey’s test to **a**; unpaired two-tailed Student’s *t* test to **f**, **g**, **h**, **j** and **k**. *P* values are shown

The roles of Nur77/GSDMC-mediated pyroptosis in tumor growth were further investigated in mouse models. Nude mice implanted with A375 cell-derived xenografts were used to determine the antitumor potential of DdBIC in vivo. Intraperitoneal injection of DdBIC significantly reduced xenograft tumor growth in a dose-dependent manner (Fig. 7a), with GSDMC and OPA1 cleavage and eIF2α phosphorylation (Fig. 7b). DdBIC inhibited tumor growth by inducing pyroptosis with no obvious side effects, as the weights of the body, heart, liver, spleen, lung, and kidney were maintained within the normal ranges (Supplementary Fig. 8a, b). Additionally, serum biomarkers indicative of cardiac (CK-MB), hepatic (ALT and AST), and renal (BUN) injuries were assessed during treatment with DdBIC, and the results revealed no significant alterations in these markers (Supplementary Fig. 8c), indicating that DdBIC did not induce detectable toxic effects. Furthermore, another acute toxicity experiment demonstrated that even intraperitoneal administration of DdBIC at a dose of 2000 mg/kg did not result in mortality in mice (Supplementary Fig. 8d). Collectively, these findings support a favorable safety profile for DdBIC.

This DdBIC antitumor effect was dependent on Nur77- and GSDMC-mediated pyroptosis, as the knockdown of either Nur77 or GSDMC in A375 cells mitigated the suppression of tumor growth (Fig. 7c). Notably, the knockdown of Nur77, but not GSDMC, increased the heme level, which was decreased by DdBIC (Fig. 7e). Consistent with these findings, the knockdown of Nur77 but not GSDMC blocked S-OPA1 accumulation while decreasing the level of eIF2α phosphorylation (Fig. 7d). These findings suggest that Nur77 serves as the direct target through which DdBIC regulates heme levels and related downstream factors, and GSDMC is the final executioner of pyroptosis induction. In Nur77 knockdown A375 cells, reintroducing Nur77^4mt^, which cannot bind DdBIC, did not inhibit the growth of xenograft tumors (Fig. 7f). In these xenograft tumors, Nur77^4mt^ attenuated the ability of DdBIC to decrease the heme level, induce eIF2α phosphorylation, and cleave OPA1 and GSDMC (Fig. 7g). In another mouse model, reintroduction of GSDMC^D276A^, which could not induce pyroptosis, also led to neither inhibition of xenograft tumors (Fig. 7h) nor cleavage of GSDMC even in the presence of DdBIC (Fig. 7i). Together, these data consistently demonstrate that Nur77 and GSDMC mediate DdBIC-induced pyroptosis in vivo.

Induction of pyroptosis is a conduit to antitumor immunity.^8^ To test DdBIC-stimulated immunity in the tumor microenvironment, a C57BL/6J mouse model with murine melanoma B16 tumors was generated because DdBIC could effectively induce pyroptosis in mouse B16 cells (Supplementary Fig. 8e). The intraperitoneal injection of DdBIC significantly suppressed tumor growth (Fig. 7j, top), with related events, including GSDMC and OPA1 cleavage, eIF2α phosphorylation, and a decrease in heme levels, detected in the same tumor samples (Fig. 7j, bottom). The immune cells were isolated from the tumor tissues to analyze immune cell infiltration and activation. Although the proportions of CD4^+^ T and CD8^+^ T cells were approximately the same, the treatment increased the abundance of NK cells in the TME. The activation status of these cell populations was also observed, as the proportions of PFN (perforin)^+^/CD8^+^, IFNγ^+^/CD8^+^, GZMB^+^/CD8^+^, TNF-α^+^/CD8^+^ T cells and GZMB^+^ NK cells increased (Fig. 7k and Supplementary Fig. 8f). This DdBIC-induced pyroptosis clearly contributed to the stimulation of antitumor immune responses. To evaluate the potential synergistic effect of DdBIC with an anti-PD-1 antibody, C57BL/6 mice bearing B16-derived allografts were treated with DdBIC either alone or in combination with an anti-PD-1 antibody. The results demonstrated that DdBIC effectively inhibited tumor growth, and the addition of an anti-PD-1 antibody significantly enhanced this effect, resulting in a synergistic antitumor response (Fig. 7l). These findings further support the translational potential of DdBIC as a promising therapeutic agent.

To further validate the role of Nur77 in the translational potential of DdBIC, we examined Nur77 expression levels in clinical melanoma tissue samples. The results revealed that Nur77 expression was significantly higher in melanoma tissues than in nonmelanoma nevus tissues (Supplementary Fig. 8g). Furthermore, analysis of TCGA data indicated that Nur77 expression is markedly elevated in metastatic melanoma tissues compared with primary melanoma tissues (Supplementary Fig. 8h). These findings not only demonstrate the increased expression of Nur77 during melanoma progression but also indicate that DdBIC has translational potential for the treatment of melanoma, particularly metastatic melanoma, through Nur77 regulation.

## Discussion

**Fig. 8:**
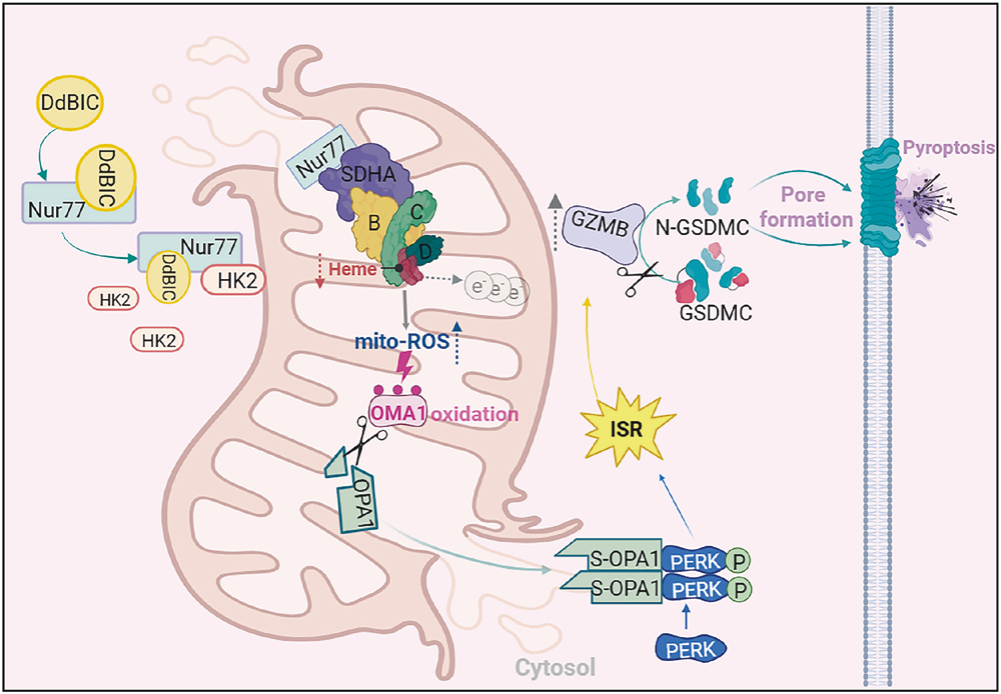
Working model for the DdBIC function. DdBIC directly targets the nuclear receptor Nur77. With the assistance of HK2, Nur77 is translocated into the mitochondria to negatively regulate the heme level by increasing the activity of SDHA, which causes leakage of electrons, leading to increased mito-ROS. The mitochondrial protease OMA1 senses this ROS signal via oxidation and then cleaves its downstream OPA1, which converts OPA1 into S-OPA1 that subsequently releases into the cytoplasm. Cytosolic S-OPA1 activates PERK to cause an integrated stress response (ISR) and then activates granzyme B activity, which further cleaves GSDMC and induces pyroptosis. Figure 8, created using BioRender.com

Pyroptosis-induced tumor cell death is a new avenue for treating tumors, especially apoptosis-resistant tumors, such as melanoma. Since the lead compounds that trigger GSDMC-mediated pyroptosis have not been reported, we used the chemical compound DdBIC as a tool to explore its regulatory network and functional mechanism in pyroptotic induction through GSDMC execution. In the mitochondria, DdBIC disrupted the homeostasis of heme through the activation of the hemoprotein SDH complex, leading to electron leakage to produce mito-ROS. Sensing this mito-ROS signal by OMA1 cleaves its downstream OPA1 and leads to OPA1 release into the cytoplasm, subsequently causing ISR, which induces pyroptosis through activating granzyme B-dependent GSDMC cleavage. With the assistance of HK2 as a molecular chaperone, Nur77 was translocated into the mitochondria to downregulate heme levels via the activation of the SDH complex (Fig. 8). Together, the results of this study reveal an unreported signaling pathway involved in GSDMC-dependent pyroptosis, from the disruption of heme homeostasis to ISR-mediated granzyme B activation.

Many cancer cells increase the levels of heme and activate oxygen-utilizing hemoproteins by increasing the expression levels of proteins involved in heme synthesis.^57^ A higher level of intracellular heme buttresses tumorigenic functions, whereas a reduced level of heme contributes to the induction of either apoptosis or ferroptosis.^58,59^ In addition to previous studies on heme-associated cell death via various mechanisms, this study contributes a unique mechanism of DdBIC-induced pyroptosis in conjunction with the modulation of heme levels. The activation of the SDH complex by DdBIC led to excessive consumption of succinate, resulting in reduced levels of succinyl-CoA and consequently impairing de novo heme synthesis. This impairment limits the availability of heme groups required for the proper assembly and function of the SDH complex. Concurrently, the oxidation of succinate generates substantial electron flux; however, due to insufficient heme supply, electron leakage occurs, leading to a significant increase in mito-ROS. Given that DdBIC-associated negative regulation of heme levels through the SDH complex led to pyroptosis induction, a new strategy involving regulating heme levels in tumors to widen the therapeutic window could be exploited.

Unexpectedly, DdBIC directly targeted the nuclear receptor Nur77 to promote its interaction with SDHA and modulate the heme level. Nur77 functions either as a transcription factor in the nucleus or as a regulator in the cytosol. Nur77 has no targeting sequence to mitochondria, and its targeting to mitochondria is assisted by other chaperone proteins, such as Bcl-2 for apoptosis and Nix for autophagy,^18,60^ and HK2 for pyroptosis in the current study, demonstrating that different adapters direct Nur77 to different mitochondrial domains for different functions. Interestingly, DdBIC binds in the same pocket as another compound, THPN, in the Nur77 ligand binding domain (LBD), and both compounds direct Nur77 to the inner mitochondrial membrane. However, the orientations of the “heads” of these two compounds have an approximately 90-degree rotational difference. This difference in the “head” orientation of the compounds may be a major determinant of the interaction of Nur77 with either HK2 for the induction of pyroptosis or Nix for autophagy. Despite its unique structural characteristics, DdBIC is an important investigative tool that facilitates the understanding of a novel signaling pathway involved in pyroptosis induction. It will be valuable to further explore whether this DdBIC-induced pyroptotic pathway is also activated under other physiological or pathological conditions in future research.

The mechanism of ISR activation is usually mediated by regulating eIF2α phosphorylation under diverse stress conditions, such as ER stress and heme deficiency. However, how mitochondrial signals trigger the activation of ISR is unclear. Typically, PERK is activated by ER stress, which dissociates the BiP-PERK complex to facilitate the dimerization and autophosphorylation of PERK.^44,56^ Here, we revealed a previously unreported pathway in which PERK dimerized upon interaction with cytosolic S-OPA1, leading to PERK autophosphorylation and activation. The presence of S-OPA1 in the cytoplasm is achieved through the coordinated action of two parallel mitochondrial perturbation pathways: one is to induce mPTP opening, resulting in mitochondrial swelling and eventual rupture; the other is to promote OPA1 cleavage and subsequent S-OPA1 generation via mito-ROS-dependent activation of OMA1. When OPA1 is released into the cytoplasm from damaged mitochondria, it forms a dimer by itself and subsequently interacts with PERK to facilitate the formation of PERK dimers, which in turn activates PERK activity through autophosphorylation. Therefore, ISR activated by cytosolic OPA1-PERK signalosomes may be a primary hallmark of mitochondrial dysfunction. Although robust activation of PERK is sufficient to induce pyroptosis, a lower level of PERK activation by ER stress inducers does not result in pyroptosis, suggesting that the threshold value of PERK activation may determine cellular fate, specifically cell survival versus cell death.

Serine protease granzymes are expressed mainly in NK cells and toxic T lymphocytes and kill tumor cells along with perforin once they are secreted from immune cells.^48^ Wang et al. reported that granzyme B derived from T cells activates caspase-6 in triple-negative breast cancer cells, which further cleaves GSDMC at the Asp365 residue.^46^ However, the importance of tumor-derived granzyme B is largely unknown because of its relatively low expression in tumor cells. We observed that DdBIC directly induced granzyme B expression and activity in melanoma cells, which is distinct from the commonly recognized T-cell-derived granzyme B, highlighting the cell-autonomous role of endogenous granzyme B in GSDMC cleavage. However, the knockdown of granzyme B only partially reduced DdBIC-induced LDH release, suggesting that other proteases, including but not limited to granzymes, may compensate for the loss of granzyme B. Together, these findings not only emphasize the function of granzyme B in cancer cells but also provide a new paradigm for triggering pyroptosis via granzyme B cleavage of GSDMC in response to various stimuli.

We found that DdBIC treatment-induced pyroptosis in the tumor model was closely associated with the activation of CD8^+^ T cells and NK cells, which is consistent with the established concept that pyroptosis functions as an immunogenic cell death mechanism capable of activating antitumor immunity.^8^ However, it remains unclear whether DdBIC can induce durable immune memory, a question that warrants further investigation through tumor rechallenge studies in future work. Nevertheless, our study highlights the therapeutic potential of DdBIC in melanoma treatment.

## Materials and methods

### Screen of compounds

On the basis of the scaffold of cytosporone B (Csn-B), the first Nur77 agonist identified by our group in 2008,^19^ we constructed a small-molecule compound library. In this study, A375 cells were incubated with compounds (total of 2371 compounds) at a concentration of 20 μM for 24 h, after which the pyroptotic morphology was observed under a light microscope. The resulting 63 compounds with typical pyroptotic morphologies were chosen for the next screening round, where an LDH release assay was employed to evaluate their efficacies in pyroptosis in both control and GSDMC-knockdown A375 cells. The results demonstrated that one compound, DdBIC, lost its capacity to induce LDH release in GSDMC-knockdown cells, which was nearly comparable to that of the control. Finally, the other pyroptosis-executing proteins, GSDM A/B/D/E, were individually knocked down to further exclude their roles in pyroptotic induction by DdBIC.

### RNA isolation, RT‒PCR and primers

Total RNA was extracted by using Vezol reagents (Vazyme, Cat# R411-01), and cDNA was synthesized with a reverse transcription kit (ABclonal). The cDNA was used as a template for amplification, and the level of actin was used as a normalization control. Real-time PCR was performed via NovoStart® SYBR qPCR SuperMix (Novoprotein) according to the manufacturer’s instructions. The primer sequences for real-time PCR were shown in supplymentary file.

### Immunoprecipitation and Western blotting analysis

Immunoprecipitation was performed as previously described.^34^ In brief, the cells were lysed on ice with ELB lysis buffer (150 mM NaCl, 100 mM NaF, 50 mM Tris-HCl (pH 7.6), 0.5% NP40, and 1 mM PMSF) containing protease inhibitors and a phosphatase inhibitor cocktail. After centrifugation at 12,000 × *g* for 30 min, the supernatant was incubated with the appropriate antibody and protein G-Sepharose beads for 3 h at 4 °C. The beads were collected and washed three times with ELB lysis buffer and then subjected to western blotting. For western blotting, the cells were lysed with lysis buffer (20 mM Tris (pH 7.5), 150 mM NaCl, 1 mM EDTA, 1 mM EGTA, 2.5 mM sodium pyrophosphate, 1% Triton X-100, and 1 mM PMSF) and boiled in SDS loading buffer for 10 min. The protein samples were subjected to SDS-PAGE and transferred to polyvinyl difluoride (PVDF) membranes, which were incubated with primary antibodies and then with secondary antibodies. The final immunoreactive products were detected by enhanced chemiluminescence (NCM). For BN-PAGE detection, the cytosolic fraction was lysed in buffer (10 mM HEPES-KOH (pH 7.4), 10% glycerol, 50 mM NaCl, 2.5 mM MgCl_2_, and 1% digitonin). The lysate was mixed with 10× NativePAGE sample buffer (5% Coomassie Brilliant Blue G-250, 0.5 M 6-aminocaproic acid, 100 mM Bis-Tris/HCl (pH 7.0), and 10% glycerol) without heat denaturation. The samples were resolved on a NativePAGE 4–6% Bis-Tris gel (Invitrogen) using anode buffer (50 mM Bis-Tris/HCl (pH 7.0)) and cathode buffer (50 mM Tricine, 15 mM Bis-Tris/HCl (pH 7.0), and 0.02% Coomassie Blue G-250). Electrophoresis was initially performed at 90 V for 3 h. The cathode buffer was then replaced with a cathode buffer lacking Coomassie Blue G-250, and electrophoresis continued at 150 V overnight. The gels were subsequently incubated in 1% SDS at 50 °C for 30–60 min. The proteins were subsequently transferred to a 0.45 µm PVDF membrane at 100 V for 2 h. All subsequent steps for immunodetection were performed following standard SDS-PAGE western blotting procedures.

OPA1 and PERK dimerization was assessed under denaturing and nonreducing conditions. Specifically, the cells were lysed in 2× SDS loading buffer (0.1 M Tris, 20% glycerol, 4% SDS, and 0.02% bromophenol blue) and split into two equal aliquots. The reduced group sample was supplemented with β-mercaptoethanol and subjected to SDS-PAGE. The nonreduced samples were separated via 4–12% gradient separation gel electrophoresis (Sangon Biotech).

### Microscopy

The cells were inoculated in 12-well plates at a density of 40–60% to observe the morphology of the pyroptotic cells. Phase contrast images were captured via a Nikon microscope.

For confocal microscopy, cells grown on glass coverslips were fixed with 4% paraformaldehyde. After fixation, cells were permeabilized and blocked in a solution of 3% BSA with 0.2% Triton X-100, followed by an overnight incubation at 4 °C with the indicated primary antibodies. Subsequently, cells were washed and incubated for 1 h at room temperature with Texas Red- or FITC- conjugated secondary antibodies (Life Technologies). Nuclei were counterstained with DAPI for 2 min. Images were acquired using a Zeiss LSM 780 confocal microscope.

The mitochondrial morphology was observed via confocal microscopy. Mitochondrial swelling was characterized by Tom20 immunostaining with circular, donut-like structures and HSP60 immunostaining with a punctate appearance. These swollen phenotypes (indicated by Tom20 immunostaining with circular, donut-like structures) were quantified by scoring 100 cells across five random fields. The quantification was performed three times per experiment, with the average value representing a single replicate (n = 3; mean ± s.e.m.).

### Measurement of cell viability

Pyroptosis was indicated by the release of LDH into the culture supernatant via CytoTox 96 non-radioactive cytotoxicity assay kits (Promega) according to the manufacturer’s protocol.

The intracellular ATP content was measured via the Cell Titer-Glo Assay (Promega). After 24 h of treatment, the cells were trypsinized, centrifuged, and resuspended. A 50 μL aliquot of the cell suspension was combined with assay reagent and incubated for 15 min at room temperature, after which the luminescence was recorded.

### Isolation of the cytosolic fraction and mitochondria

The isolation of the cytosolic fraction was performed as described previously.^34^ In brief, the cells were first scraped off the dishes, collected in ice-cold PBS, and washed twice with ice-cold PBS. The cells were then resuspended in 200 μL of digitonin lysis buffer (75 mM NaCl, 1 mM NaH_2_PO_4_, 8 mM Na_2_HPO_4_, 250 mM sucrose and 190 μg/mL digitonin). After 5 min on ice, the cells were centrifuged at 12,000 × *g* for 5 min at 4 °C, and this process was repeated three times. The supernatant was collected and analyzed by western blotting.

For the isolation of mitochondria, the cells were collected with trypsin and centrifuged at 825 × *g* for 5 min at 4 °C. The cell pellets were resuspended in mitochondrial isolation buffer (250 mM mannitol, 0.5 mM EGTA and 5 mM HEPES-KOH (pH 7.4)) and then homogenized with a douncer. The homogenates were centrifuged three times at 700 × *g* for 10 min at 4 °C to remove unbroken cells, cell debris and nuclei. The supernatant was centrifuged at 7000 × *g* for 30 min at 4 °C to enrich the mitochondria, and the mitochondrial pellets were resuspended in isolation buffer and centrifuged at 12,000 × *g* for 10 min at 4 °C three times. The pellets were lysed with 2× loading buffer and further analyzed by SDS-PAGE.

For the proteinase K protection assay, isolated mitochondria were resuspended in mitochondrial isolation buffer and then split into two equal aliquots. One group of samples was incubated with 10 μg/mL proteinase K for 30 min on ice. The reaction was stopped by the addition of 7 mM phenylmethanesulfonyl fluoride (PMSF), and the reactants were further analyzed via SDS-PAGE.

### DdBIC level analysis by LC-MS

The concentrations of DdBIC in the cytoplasm and mitochondria were determined via LC-MS analysis. A375 cells were harvested after DdBIC treatment for 30 min, and equal amounts of proteins from fresh mitochondria and the cytoplasm were isolated (see above “Isolation of the cytosolic fraction and mitochondria” procedure). A375 cells were washed twice with ice-cold PBS and frozen in liquid nitrogen, after which DdBIC in each sample was extracted with 80% methanol solution, vortexed for 1 min and centrifuged at 14,000 × *g* for 10 min at 4 °C. The supernatants were evaporated to dryness with a vacuum centrifuge (Labconco Corporation). The samples were resuspended in 200 μL of 50% acetonitrile, centrifuged at 14,000 × *g* for 10 min at 4 °C, and further analyzed with a Thermo Scientific™ Q Exactive™.

### Protein purification

Proteins were expressed in BL21 (DE3) *E. coli* cells harboring the construct with 0.2 mM isopropyl-β-D-thiogalactopyranoside at 18 °C for 16 h. Bacterial cells were collected and resuscitated with His lysis buffer (50 mM NaH_2_PO_4_, 300 mM NaCl, and 10 mM imidazole, pH 8.0) for His-tagged proteins (Nur77-LBD^wt^ and Nur77-LBD^4mt^). The bacterial cells were subsequently lysed via sonication and centrifuged, after which the supernatant containing the recombinant proteins was incubated with HIS-Select® nickel affinity gel (Sigma, Cat# p6611) at 4 °C for 2 h. After washing with washing buffer (50 mM NaH_2_PO_4_, 300 mM NaCl, and 10 mM imidazole, pH 8.0), the proteins were eluted with elution buffer (50 mM NaH_2_PO_4_, 300 mM NaCl, and 250 mM imidazole, pH 8.0).

### Measurement of OMA1 activity

The OMA1 activity was assayed as previously described.^61^ Briefly, a mixture (100 µL) was prepared and placed in a 96-well plate. The following reagents were pipetted sequentially: OMA1 assay buffer (50 mM Tris/HCl (pH 7.5) and 40 mM KCl), protein sample, and either vehicle or 200 µM TPEN. Reactions were initiated by adding the OPA1 fluorogenic substrate (LifeTein LLC) to a final concentration of 5 µM. The plates were immediately transferred to a prewarmed (37 °C) plate reader, and the relative fluorescence units (RFUs) were measured every minute for 30 min (λ_ex_ 320 nm/λ_em_ 405 nm).

### Assessment of mPTP opening

mPTP opening was quantified via the calcein-AM/CoCl₂ quenching method. Briefly, the cells were incubated with 1 μM calcein-AM and 5 mM CoCl₂ for 15 min at 37 °C in the dark, leading to quenching of the cytosolic and nuclear calcein. Mitochondrial calcein fluorescence, which reflects the mPTP status, was then measured at 517 nm (excitation of 494 nm) via a spectrometer.

### Detection of lysosomal membrane permeabilization (LMP)

LMP was detected via two methods. The first method relies on the release and de-quenching of acridine orange (AO). After DdBIC treatment, the cells were incubated with AO (5 μg/mL) for 20 min at 37 °C in the dark and washed three times with FBS-free DMEM, after which the resulting red fluorescence intensity was quantified. The second method tracks the recruitment of galectin-3 to damaged lysosomes. Cells expressing GFP-Galectin-3 were treated with DdBIC, and the formation of Galectin-3 puncta (indicating LMP) was observed under a confocal microscope.

### Analysis of the mitochondrial membrane potential

The mitochondrial membrane potential was monitored via the cationic dye JC-1, which exhibits a potential-dependent shift in fluorescence from green (monomer) to red (aggregate). After DdBIC treatment, the cells were incubated with JC-1 in serum-free DMEM for 20 min at 37 °C in the dark. The cells were then detached via trypsinization and immediately analyzed via flow cytometry.

### Measurement of ATP

The intracellular ATP concentration was quantified via a bioluminescence-based assay (ENLITEN ATP Assay System Bioluminescence Detection Kit, Promega). In brief, cells (2 × 10⁴) were plated in 6-well plates and allowed to adhere for 24 h. After treatment with DdBIC, the cells were harvested in 300 μL of 2% trichloroacetic acid, followed by centrifugation at 20,000 × *g* for 3 min at 4 °C to collect the supernatant. The resulting supernatants were neutralized with an equal volume of 0.33 M KOH and subsequently diluted 20-fold in 0.1 M Tris-acetate buffer. A 20 μL aliquot of the diluted supernatant was then assayed for ATP content via a luminometer (Promega).

### Cleavage of GSDMC in the in vitro assay

To verify the in vitro cleavage of GSDMC by granzyme B, recombinant GSDMC (1 μg) was incubated with active recombinant granzyme B in 10 μL of reaction buffer (50 mM Tris-HCl pH 8.0 and 150 mM NaCl) for 3 h at 37 °C. The reaction samples were subjected to western blotting.

### Granzyme B activity assay

The cells were lysed in 150 μL of Triton X-100 lysis buffer (1% Triton (v/v) in PBS) and centrifuged at 12,000 × *g* for 15 min, after which the supernatant was incubated with quenched fluorescence peptide substrates (Ac-IEPD-AMC TFA). Granzyme B activity was assayed via a fluorescence plate reader.

### Identification of DdBIC-binding proteins

A375 cells from fifty 10-cm dishes were lysed with HEPES lysis buffer (50 mM HEPES, pH 8.0, 150 mM NaCl, and 1% (v/v) NP40) and centrifuged at 12,000 × *g* for 30 min at 4 °C. The resulting supernatants were incubated with either DdBIC-P or DdBIC-NP for 2 h at 4 °C, and then, a click chemistry reaction was performed with the solution (0.1 mM biotin-N_3_, 1 mM CuSO_4_, 1 mM TCEP and 0.1 mM TBTA) for 2 h. To remove protein aggregates, the reactants were centrifuged at 12,000 × *g* for 15 min at 4 °C and then incubated with NeutrAvidin beads (Thermo) for 2 h under gentle rotation. After centrifugation, the beads were washed with HEPES lysis buffer three times and lysed with 2× loading buffer. The samples were finally subjected to SDS-PAGE and stained with Coomassie blue before MS analysis.

### TurboID assay

In brief, DdBIC-treated cells were incubated with 100 μM biotin for 10 min at 37 °C in serum-free culture medium. The culture medium was then replaced with medium containing 10% FBS and incubated with the cells for 3 h. Biotin-labeled proteins were purified via streptavidin magnetic beads (Thermo Fisher Scientific, 88816), and the samples were subjected to western blotting analysis.

### Analysis of protein oxidation

Protein oxidation was detected via the resin-assisted capture of S-oxidized proteins. The cells were lysed on ice for 15 min in biotin-labeling lysis buffer (BLLB: 50 mM Tris-HCl pH 7.0, 5 mM EDTA, 120 mM NaCl, and 0.5% NP40) containing protease inhibitors and 100 mM maleimide (for blocking unmodified cysteine; Sigma, 129585). After centrifugation at 13,000 × *g* for 15 min, the supernatant was transferred to a new tube, and the protein concentration in each group was adjusted to the same level. Then, the cell lysates were incubated with a final concentration of 1% SDS at room temperature for 2–3 h while rotating. Next, the cell lysates were incubated with 5 volumes of precooled acetone at −20 °C for at least 20 min. After centrifugation at 20,000 × *g* for 10 min at 4 °C, the supernatants were discarded, and the precipitated protein was air-dried. A volume of 200 μL of BLLB containing 1% SDS and 10 mM DTT (for reducing the oxidized cysteine) was added to resuspend the proteins. After incubation at room temperature for 15 min while rotating, a total of 5 volumes of precooled methanol were added to precipitate the proteins. The proteins were suspended in 500 μL of BLLB and then incubated with 20 μL of thiopropyl Sepharose 6B (for capturing free thiol-containing protein, GE, 17042001) at room temperature for 2–3 h. Finally, the beads were washed five times with washing buffer (100 mM HEPES, 1 mM EDTA, and 1% SDS, pH 7.5) containing 8 M urea, and 1× SDS loading buffer containing 50 mM DTT was added for reacting for 20 min. Finally, the proteins were subjected to western blotting.^55^

### Detection of mitochondrial ROS

The cells were incubated with MitoSOX (Invitrogen) at a final concentration of 2 μM in FBS-free DMEM for 15 min at 37 °C in the dark and then washed three times with FBS-free DMEM. Then, the cells were collected with trypsin, centrifuged at 825 × *g* for 5 min at 4 °C, and resuspended in ice-cold PBS. The mitochondrial ROS levels were analyzed via NovoCyte Quanteon (Agilent) flow cytometry.

### Measurement of succinyl-CoA

The cells were collected with cell scrapers and washed with PBS. The cell pellets were resuspended in 200 μL of PBS, and the suspension was rapidly frozen with liquid nitrogen and thawed in water more than ten times to lyse the cells. To clear cell debris, the samples were centrifuged at 700 × *g* for 30 min at 4 °C, and the resulting supernatant was used for measurement of succinyl-CoA levels with a succinyl-CoA ELISA Kit (MEIMIAN, MM-51024H2) according to the manufacturer’s protocol.

### Measurement of CoQH2

Isolated fresh mitochondria (see the “mitochondrial isolation” section above for a detailed procedure) were used for measurement of CoQH2 levels with a Mitochondrial Complex II Activity Assay Kit (Elabscience, E-BC-K150-M).

### Measurement of fumarate

The intracellular fumarate level was assayed via a fumarate assay kit (Sigma, MAK060) according to the manufacturer’s instructions. In brief, the cells were collected with cell scrapers and rapidly homogenized in 100 μL of fumarate assay buffer. The samples were centrifuged at 13,000 × *g* for 15 min to remove insoluble material. Fifty microliter samples and 100 μL of reaction mixture were added to duplicate wells of a 96-well plate for 30 min at room temperature. Finally, the absorbance was measured at 450 nm.

### Measurement of heme levels

Heme levels were measured as previously described.^62^ The fluorescence porphyrin method was used to quantify heme in the same amount of protein or number of cells. Briefly, 2 M oxalic acid was added to each sample, which was then divided into 2 equal parts. One part was heated to 95 °C for 30 min to release the iron from the heme, and the other part was kept at 25 °C. To measure heme levels in tumor samples, the tumor tissue was digested into single cells, and red blood cells were lysed. Heme levels in the SDH complex were analyzed by immunoprecipitation of SDHA. For mitochondrial heme levels, mitochondria were isolated via subcellular fractionation, followed by heme quantification via a previously described method. The porphyrin fluorescence was measured with a microplate reader (excitation: 400 nm; emission spectrum: 608 nm) after centrifugation to remove debris. The value of the unheated sample was subtracted from the value of the corresponding heated sample.

### Mouse models

All the mice were maintained on a 12 h light/12 h dark cycle with free access to food and water under specific pathogen-free (SPF) conditions. All of the animal experiments described below were approved by the Animal Ethics Committee of Xiamen University (acceptance No. XMULAC20220174). Male nude mice (BALB/c, 18–22 g, 6–7 weeks old) and C57BL/6J mice (6–7 weeks old) were obtained from the Slac Laboratory Animal Center, China.

For the A375 cell xenograft tumor model, Ctrl, Nur77 or GSDMC knockdown and WT, Nur77^4mt^ or GSDMC^D276A^ A375 cells (2 × 10^6^) were suspended in 100 μL of PBS and injected subcutaneously into either of the anterior flanks of the mice.

The B16 cell allograft tumor model was established by subcutaneously injecting 1 × 10^5^ B16 cells in 100 μL of PBS into the anterior flanks of C57BL/6J mice.

When the tumors developed to the appropriate size, the mice were divided into four groups for treatment with vehicle (corn oil), DdBIC (20 mg/kg), IgG2α or anti-PD-1 (5 mg/kg) antibody every other day for 2 weeks (intraperitoneal injection), after which the mice were sacrificed. Body weights and tumor weights were recorded.

### Immunohistochemical staining and scoring

A melanoma tissue microarray (Cat. #MME1004j, Xi’an Taibsbio), comprising 82 melanoma and 18 nonmelanoma nevus samples, was utilized. The slides were incubated overnight at 4 °C with primary antibody diluted in Tris-buffered saline containing BSA. Antigen visualization was achieved via the use of a peroxidase-labeled polymer and a substrate-chromogen solution.

Immunohistochemical staining was semiquantitatively analyzed via the immunoreactive score (IRS) system,^63^ which yields values ranging from 0 to 12. The IRS is calculated as the product of a proportion score (estimating the percentage of positive cells: 0, none; 1, <10%; 2, 10–50%; 3, 51–80%; and 4, >80%) and an intensity score (representing average staining intensity: 0: no staining; 1: yellow, 2: claybank; and 3: tawny). All samples were evaluated in a blinded manner by three independent observers, and the mean IRS was used for final analysis.

### Assessment of the in vivo adverse effects of DdBIC

To assess the adverse effects of DdBIC in vivo, serum was isolated from retro-orbital blood samples. The blood was incubated at 37 °C for 30 min and centrifuged at 2000 × *g* for 30 min to collect the serum, which was aliquoted and stored at −80 °C. Organ injury was evaluated by measuring specific serum biomarkers: liver injury was assessed via ALT (Glutamic-pyruvic Transaminase Activity Assay Kit, Acmec AC10334) and AST (Micro Glutamic-oxalacetic Transaminase Assay Kit, Acmec AC10336) activities; nephrotoxicity was evaluated by quantifying urea levels (Urea Assay Kit, Nanjing Jiancheng Bioengineering Institute, C013-2-1); and cardiotoxicity was determined via a Mouse CKMB ELISA Kit (Sangon Biotech, D721065-0096). All the assays were performed following the manufacturers’ protocols.

### Analysis of tumor-infiltrating immune cells

Following 24 h of DdBIC administration (20 mg/kg), B16-derived allograft tumors were excised and mechanically dissected into fragments. Tissue digestion was performed for 30 min using 5 mg/mL collagenase IV (Sigma-Aldrich) and 0.5 mg/mL DNase I (Sangon Biotech) in RPMI-1640 medium supplemented with 10% heat-inactivated fetal bovine serum. The digested suspension was subsequently homogenized and filtered through 70-μm cell strainers. Immune cells were isolated using discontinuous Percoll gradient centrifugation (40% and 70% layers) according to established methodologies.^8^

Purified immune cells were washed with FACS buffer (2% heat-inactivated FBS in PBS) and incubated with block buffer comprising normal mouse serum (Jackson) and anti-CD16/32 antibody (BioLegend). The cells were then allocated into two staining panels:

1. Cell-surface antibodies or fluorescent dyes panel: Cells were stained with Zombie UV™ Fixable Viability Kit (BioLegend, 423108) and the following fluorochrome-conjugated antibodies: APC-cy7-anti-CD45, BV711-anti-CD4, PE-anti-CD8, APC-anti-NK1.1 (Thermo Fisher Scientific) and FITC-anti-CD3 (BioLegend).
2. Cell-surface marker and intracellular cytokine staining: Cells were stimulated with PMA (1 μM), ionomycin (2 μM), and GolgiPlug™ (1:1000 dilution; BD Biosciences) for 3 h. Surface staining was performed using APC-cy7-anti-CD45, BV421-anti-CD4, PerCP-anti-CD8 and PE-cy7-anti-NK1.1 (BioLegend). Following fixation and permeabilization, intracellular staining was conducted with FITC-anti-GzmB, APC-anti-perforin, BV711-anti-IFNγ and BV605-anti-TNF (BioLegend). All samples were acquired on BD LRS Fortessa × 20 flow cytometer, and data analysis was performed using FlowJo software (version 10.8.1).

### Measurement of isothermal titration calorimetry

Isothermal titration calorimetry (ITC) measurements were performed at 25 °C using a MicroCal PEAQ-ITC system. The ligand DdBIC (2 mM) was titrated into the sample containing Nur77-LBD (50 μM), with both prepared in PBS. The binding isotherm was analyzed with the MicroCal PEAQ-ITC Analysis Software and fitted to a one-site binding model.

### Structure determination by X-ray crystallography

DdBIC was added to a final concentration of 1 mM (0.5% DMSO) from a 200 mM stock solution to a protein solution of 6 mg/mL. The mixture was incubated on ice for 2 h, and crystals were obtained at 4 °C in 100 mM sodium citrate, pH 4.0, 20% glycerol and 5–10% PEG4000 by hanging-drop vapor diffusion for 3–7 days. For cryoprotection, crystals were briefly soaked in a harvesting solution supplemented with 20% ethylene glycol before being flash-cooled in liquid nitrogen.

X-ray diffraction data were collected at 100 K at beamline BL18U1 of the Shanghai Synchrotron Radiation Facility (SSRF). The data were integrated and scaled using the HKL3000 package. The structure of the Nur77-LBD/DdBIC complex was determined by molecular replacement with Phaser, using the apo Nur77-LBD structure (PDB: 3V3E) as a search model. Iterative cycles of refinement and model building were performed using Refmac5 and Coot, respectively. Data collection and refinement statistics are summarized in Supplementary Table 1.

### Statistical analysis

Data are presented as the mean ± SEM from three independent experiments, unless otherwise specified. No data points or animals were excluded from the analyses. Statistical analyses were performed using GraphPad Prism 9. Comparisons between two groups were made using an unpaired two-tailed Student’s *t* test. For comparisons involving more than two groups, one-way ANOVA with Tukey’s multiple comparison test (for a single control) or two-way ANOVA with Tukey’s test (for multiple variables) was applied. The significance thresholds were considered significant (*P* < 0.05), highly significant (*P* < 0.01) or extremely significant (*P* < 0.001) and not significant (NS). All the western blot experiments were repeated at least twice.

## Supplementary information

Original uncropped images of western blot

Supplementary data.pdf

## Data Availability

The crystal structure of the Nur77-LBD in complex with DdBIC has been deposited in the Protein Data Bank (PDB) with accession code 9M2E. The mass spectrometry data are available via ProteomeXchange with identifier PXD054435. RNA sequencing data have been deposited in the GEO database under accession number GSE273365. All other data generated in this study are included in the published article and its supplementary data files.
