# Supplementary material for: Disruption of heme homeostasis by nuclear receptor Nur77 induces pyroptosis through granzyme B-dependent GSDMC cleavage
Source: Signal Transduct Target Ther. 2025 Dec 17;10:413. doi: 10.1038/s41392-025-02528-w (PMC12711971; doi:10.1038/s41392-025-02528-w)

Fig.1

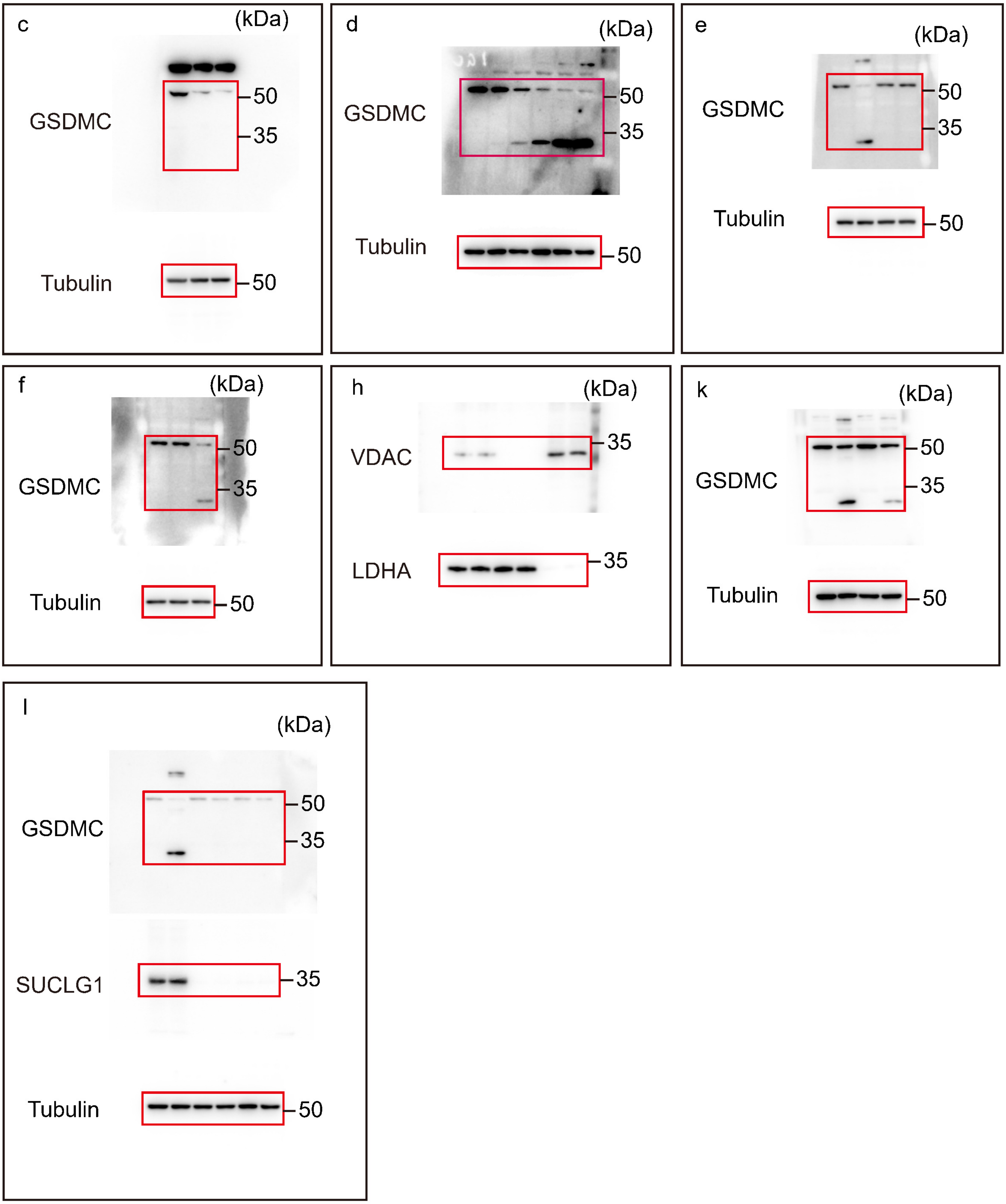
c
(kDa)
GSDMC
50
35
Tubulin
50
d
(kDa)
GSDMC
50
35
Tubulin
50
e
(kDa)
GSDMC
50
35
Tubulin
50
f
(kDa)
GSDMC
50
35
Tubulin
50
h
(kDa)
VDAC
35
LDHA
35
k
(kDa)
GSDMC
50
35
Tubulin
50
l
(kDa)
GSDMC
50
35
SUCLG1
35
Tubulin
50

Fig.2

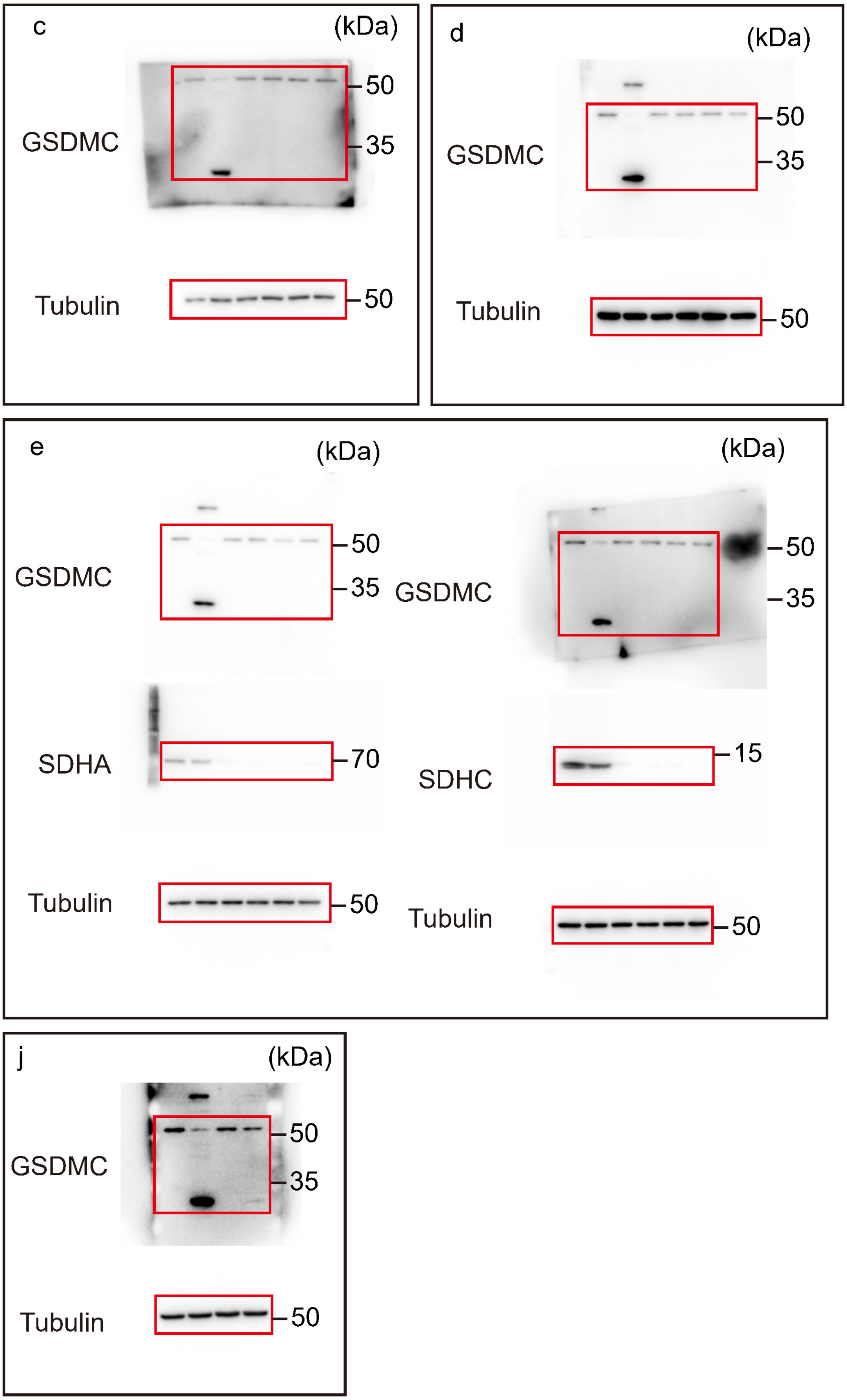
c
(kDa)
GSDMC
50
35
Tubulin
50
d
(kDa)
GSDMC
50
35
Tubulin
50
e
(kDa)
GSDMC
50
35
(kDa)
GSDMC
50
35
SDHA
70
SDHC
15
Tubulin
50
Tubulin
50
j
(kDa)
GSDMC
50
35
Tubulin
50

Fig.3-1

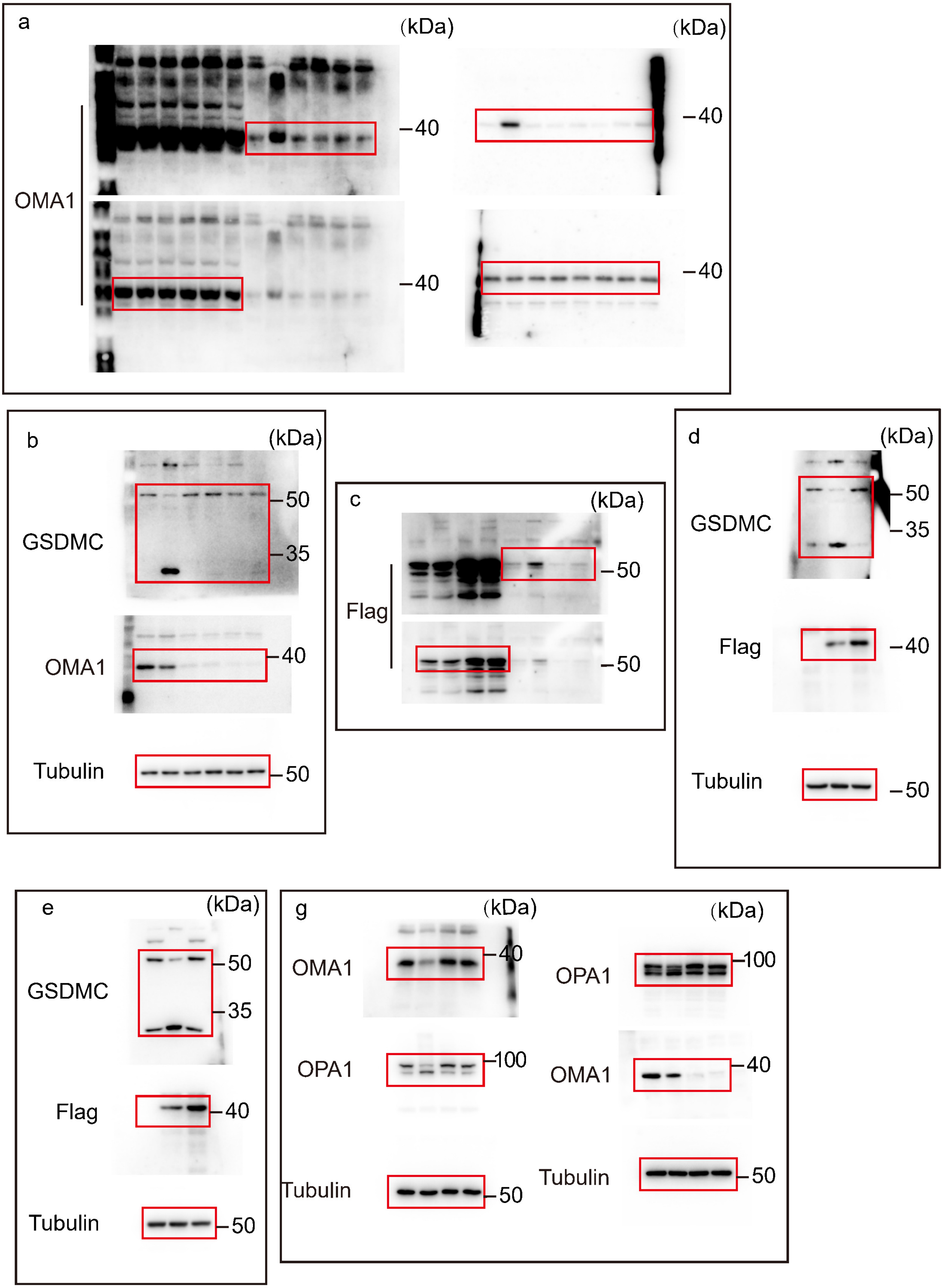
a
(kDa)
(kDa)
OMA1
40
40
40
40
b
(kDa)
GSDMC
50
35
OMA1
40
Tubulin
50
c
(kDa)
Flag
50
50
d
(kDa)
GSDMC
50
35
Flag
40
Tubulin
50
e
(kDa)
GSDMC
50
35
Flag
40
Tubulin
50
g
(kDa)
(kDa)
OMA1
40
OPA1
100
OPA1
100
OMA1
40
Tubulin
50
Tubulin
50

Fig.3-2

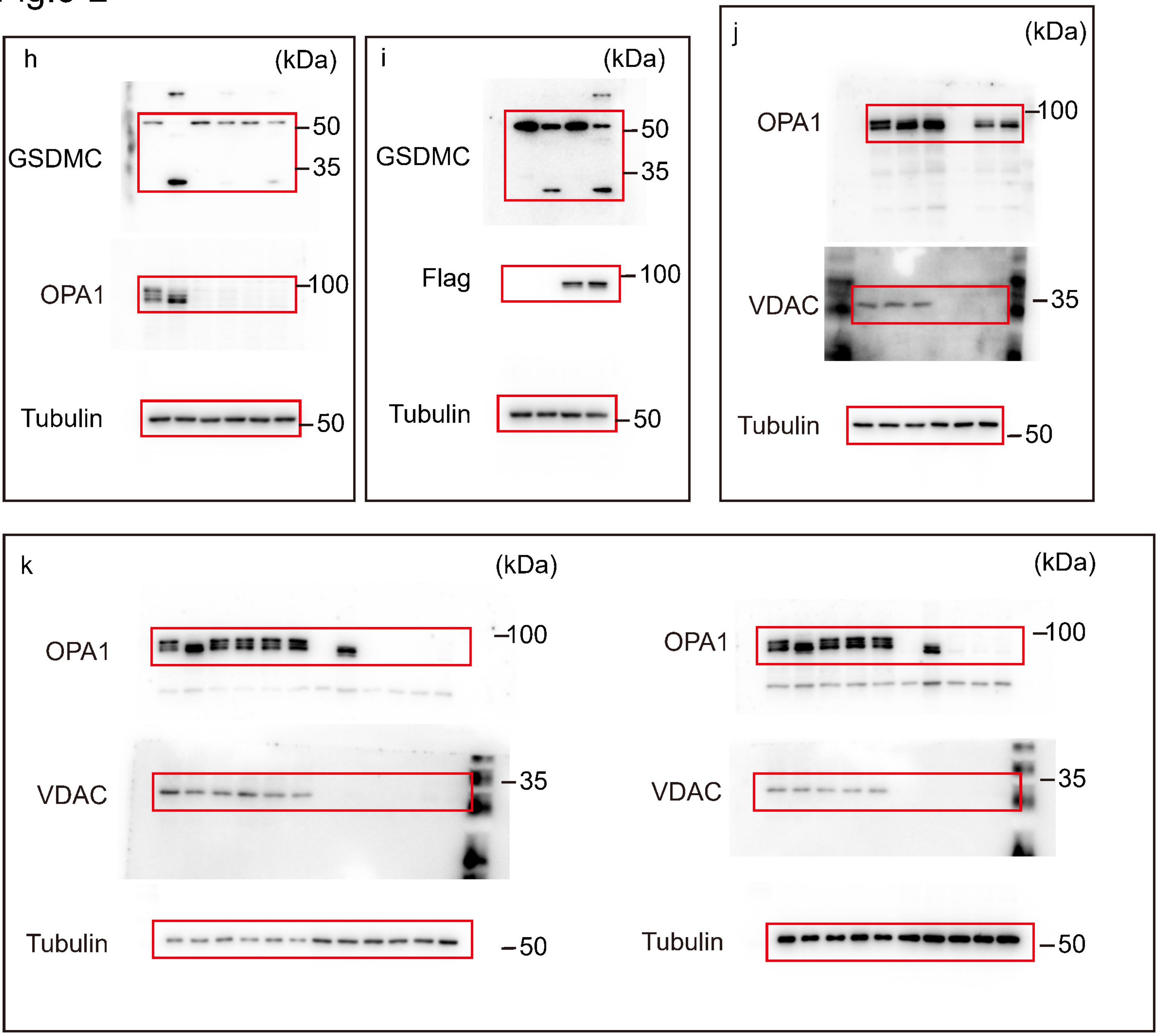
h
(kDa)
GSDMC
50
35
OPA1
100
Tubulin
50
i
(kDa)
GSDMC
50
35
Flag
100
Tubulin
50
j
(kDa)
OPA1
100
VDAC
35
Tubulin
50
k
(kDa)
OPA1
100
VDAC
35
Tubulin
50
(kDa)
OPA1
100
VDAC
35
Tubulin
50

Fig.4-1

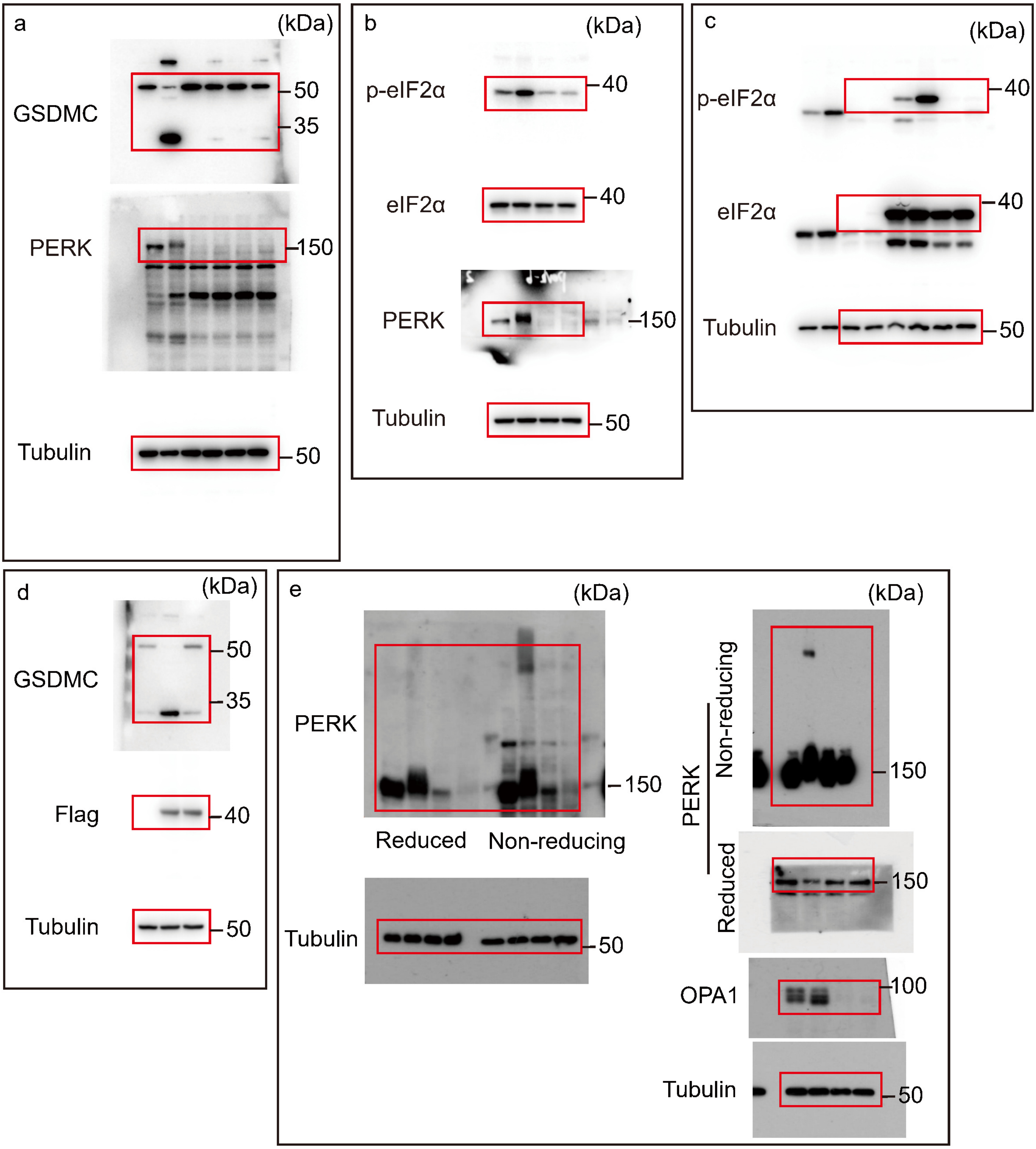

a
(kDa)
GSDMC
50
35
PERK
150
Tubulin
50
b
(kDa)
p-eIF2α
40
eIF2α
40
PERK
150
Tubulin
50
c
(kDa)
p-eIF2α
40
eIF2α
40
Tubulin
50
d
(kDa)
GSDMC
50
35
Flag
40
Tubulin
50
e
(kDa)
PERK
150
Reduced
Non-reducing
Tubulin
50
(kDa)
PERK
Non-reducing
150
Reduced
150
OPA1
100
Tubulin
50

Fig.4-2

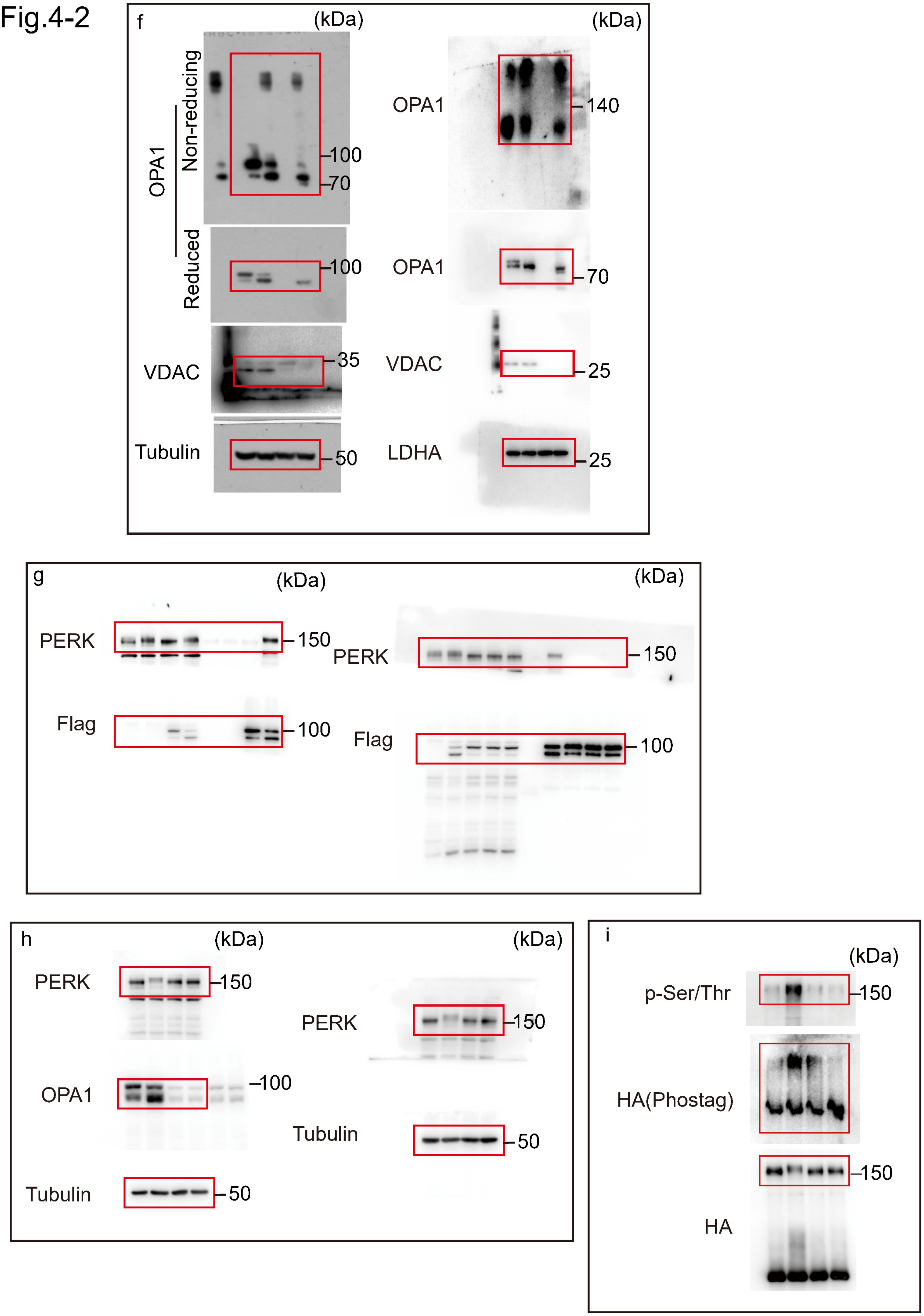

f
(kDa)
(kDa)
OPA1
Non-reducing
Reduced
100
70
100
VDAC
35
Tubulin
50
OPA1
140
OPA1
70
VDAC
25
LDHA
25
g
(kDa)
(kDa)
PERK
150
Flag
100
PERK
150
Flag
100
h
(kDa)
(kDa)
PERK
150
OPA1
100
Tubulin
50
PERK
150
Tubulin
50
i
(kDa)
p-Ser/Thr
150
HA(Phostag)
150
HA

Fig.4-3

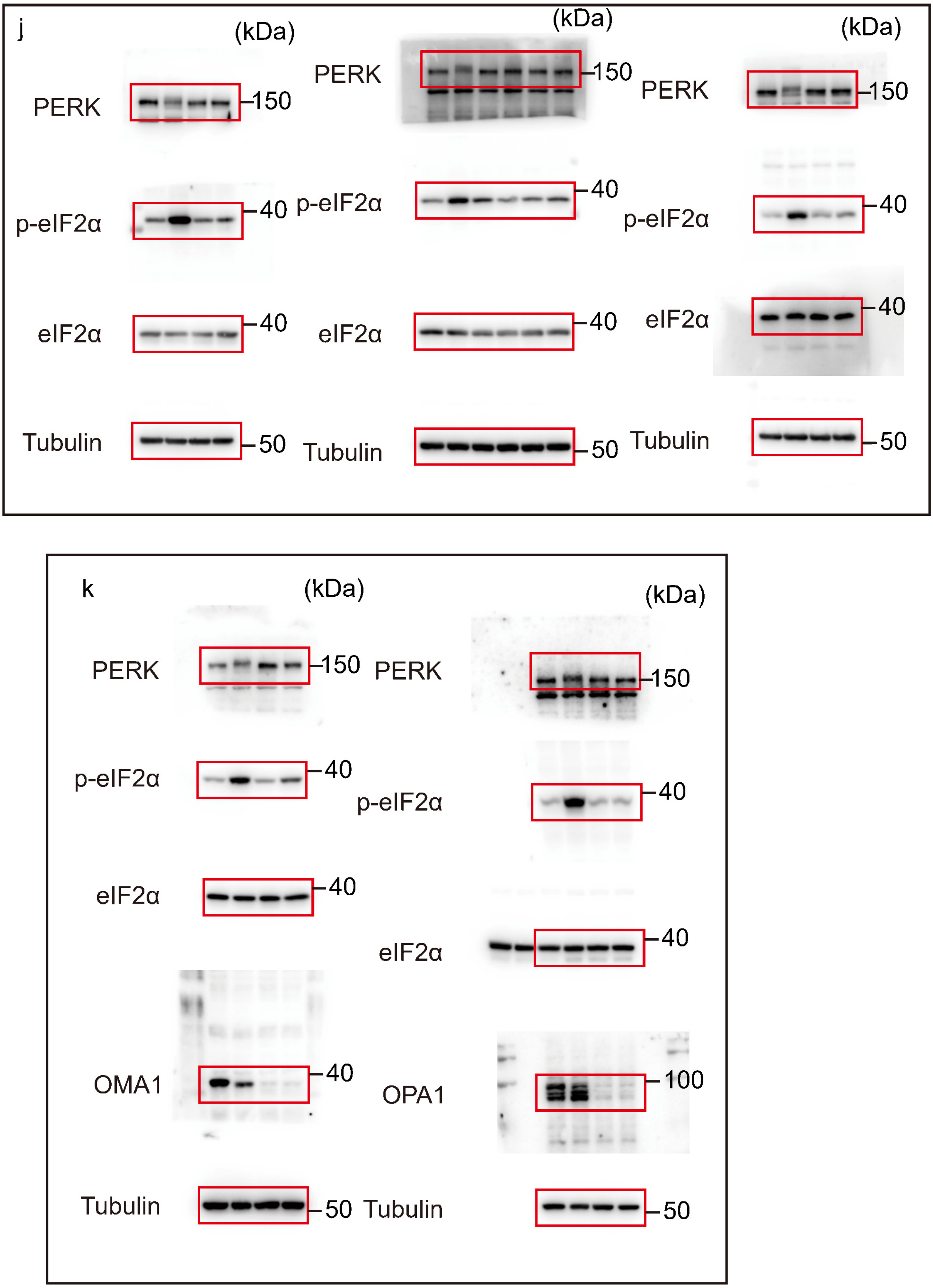
j
(kDa)
PERK
150
p-eIF2α
40
eIF2α
40
Tubulin
50
(kDa)
PERK
150
p-eIF2α
40
eIF2α
40
Tubulin
50
(kDa)
PERK
150
p-eIF2α
40
eIF2α
40
Tubulin
50
k
(kDa)
PERK
150
p-eIF2α
40
eIF2α
40
OMA1
40
Tubulin
50
(kDa)
PERK
150
p-eIF2α
40
eIF2α
40
OPA1
100
Tubulin
50

Fig.5

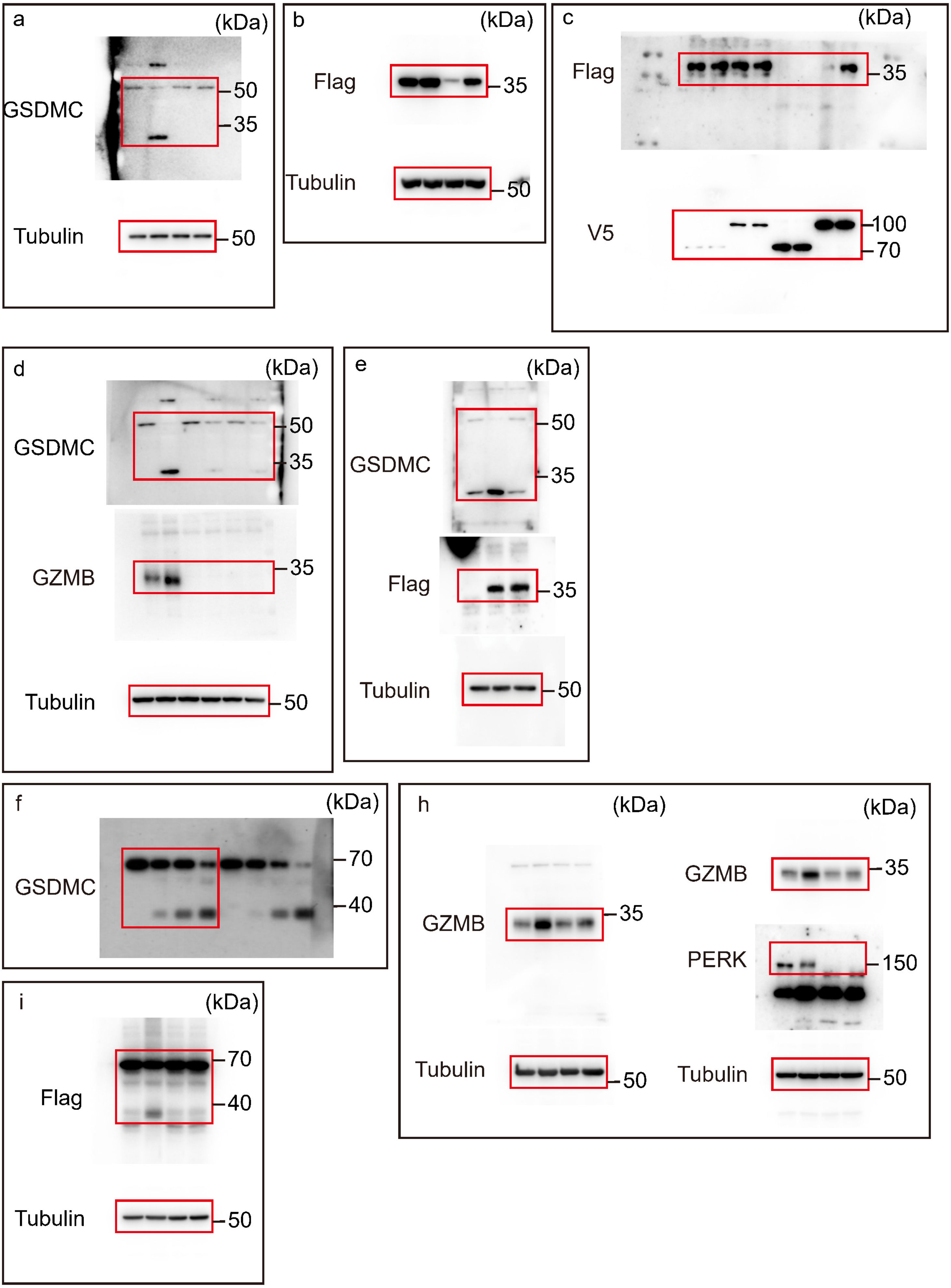

a
(kDa)
GSDMC
50
35
Tubulin
50
b
(kDa)
Flag
35
Tubulin
50
c
(kDa)
Flag
35
V5
100
70
d
(kDa)
GSDMC
50
35
GZMB
35
Tubulin
50
e
(kDa)
GSDMC
50
35
Flag
35
Tubulin
50
f
(kDa)
GSDMC
70
40
h
(kDa)
GZMB
35
Tubulin
50
(kDa)
GZMB
35
PERK
150
Tubulin
50
i
(kDa)
Flag
70
40
Tubulin
50

Fig.6-1

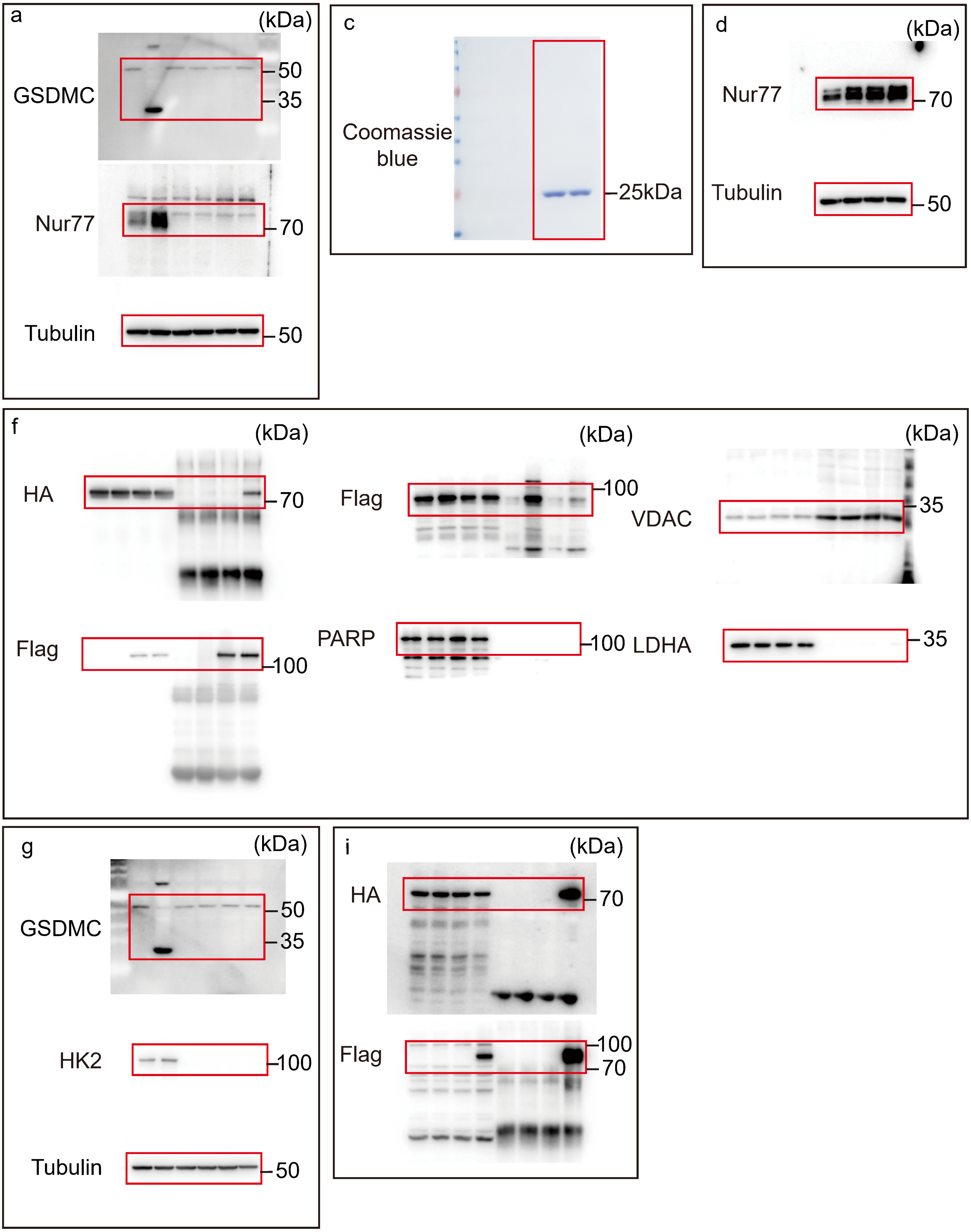
a
(kDa)
GSDMC
50
35
Nur77
70
Tubulin
50
c
Coomassie blue
25kDa
d
(kDa)
Nur77
70
Tubulin
50
f
(kDa)
HA
70
Flag
100
(kDa)
Flag
100
PARP
100
(kDa)
VDAC
35
LDHA
35
g
(kDa)
GSDMC
50
35
HK2
100
Tubulin
50
i
(kDa)
HA
70
Flag
100
70

Fig.6-2

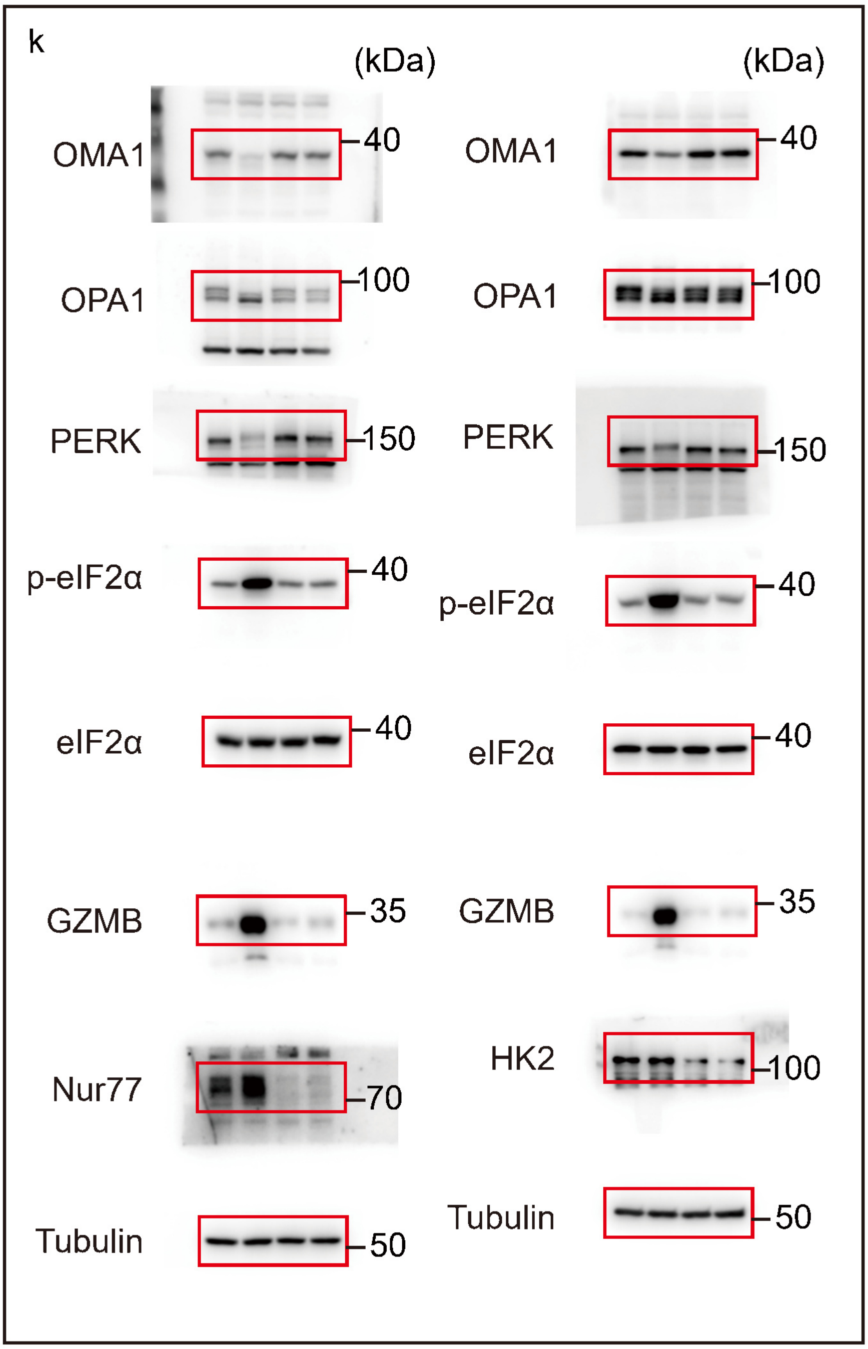

k
(kDa)
(kDa)
OMA1
40
OMA1
40
OPA1
100
OPA1
100
PERK
150
PERK
150
p-eIF2α
40
p-eIF2α
40
eIF2α
40
eIF2α
40
GZMB
35
GZMB
35
Nur77
70
HK2
100
Tubulin
50
Tubulin
50

Fig.7-1

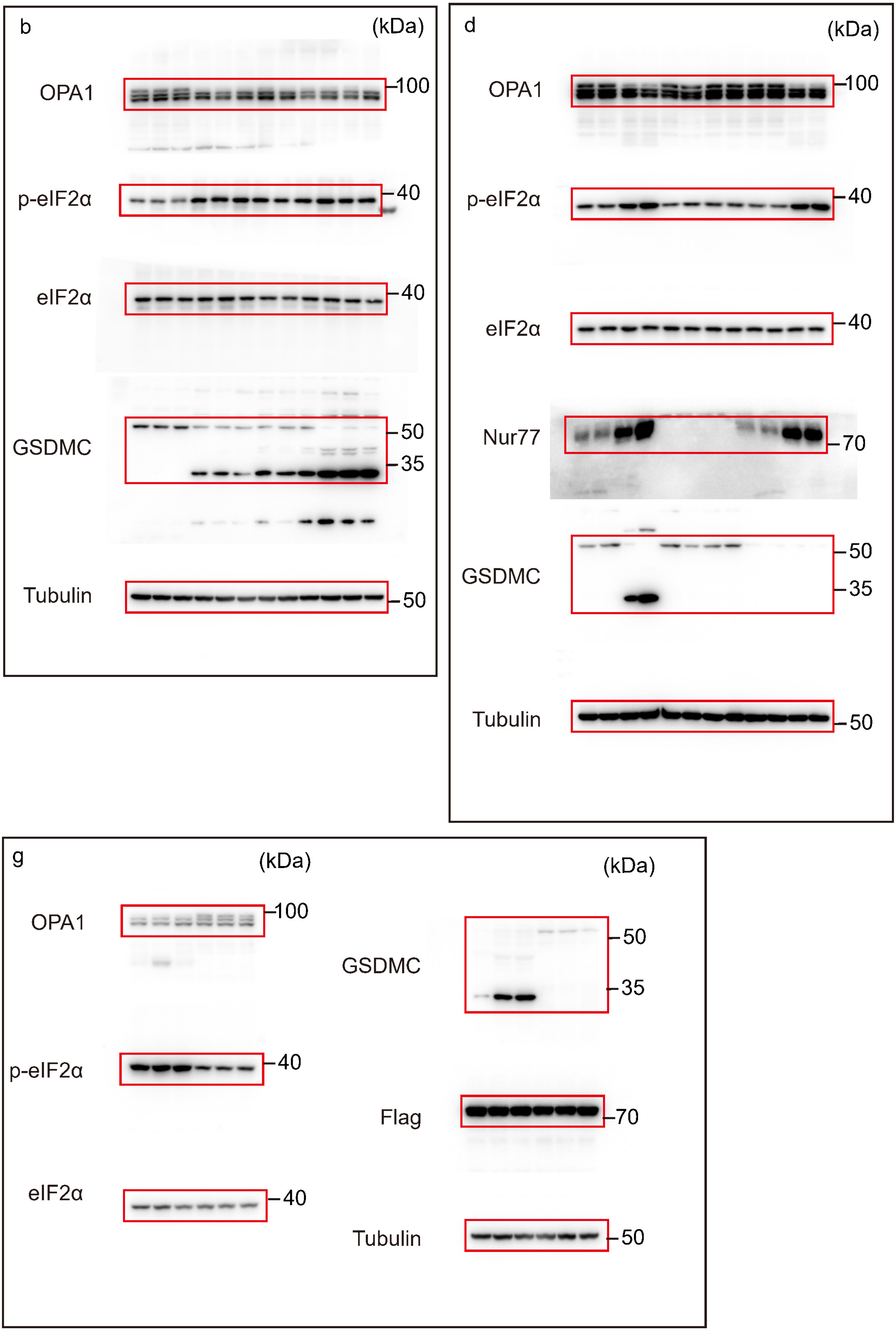
b
(kDa)
OPA1
100
p-eIF2α
40
eIF2α
40
GSDMC
50
35
Tubulin
50
d
(kDa)
OPA1
100
p-eIF2α
40
eIF2α
40
Nur77
70
GSDMC
50
35
Tubulin
50
g
(kDa)
OPA1
100
p-eIF2α
40
eIF2α
40
(kDa)
GSDMC
50
35
Flag
70
Tubulin
50

Fig.7-2

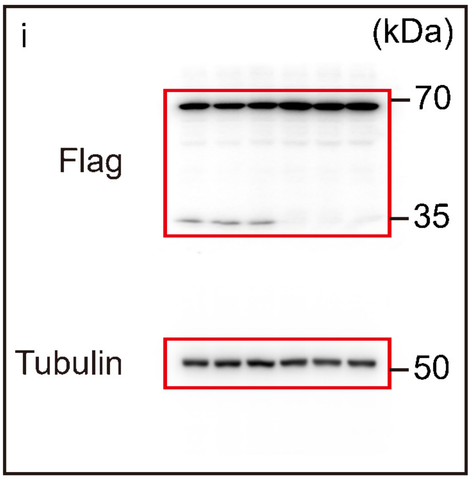

i
(kDa)
Flag
70
35
Tubulin
50

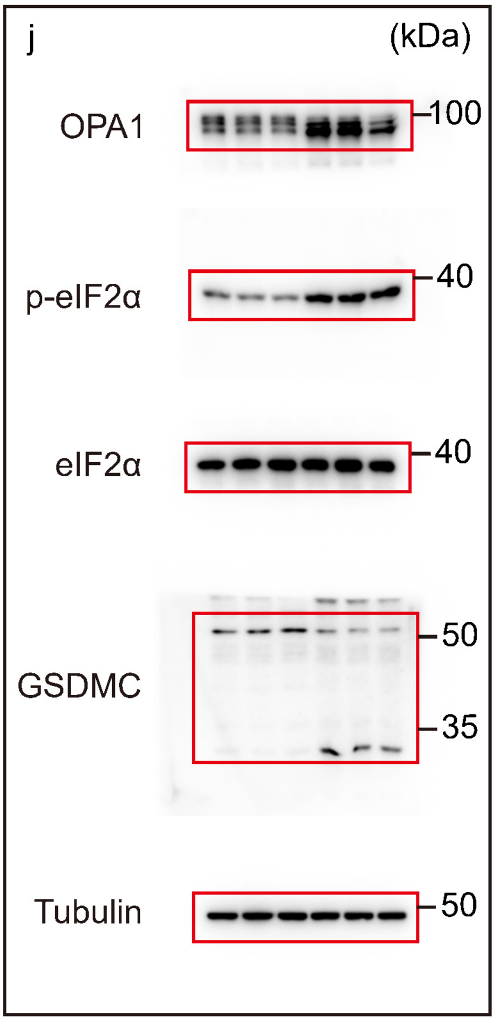

j
(kDa)
OPA1
100
p-eIF2α
40
eIF2α
40
GSDMC
50
35
Tubulin
50

Fig.S1

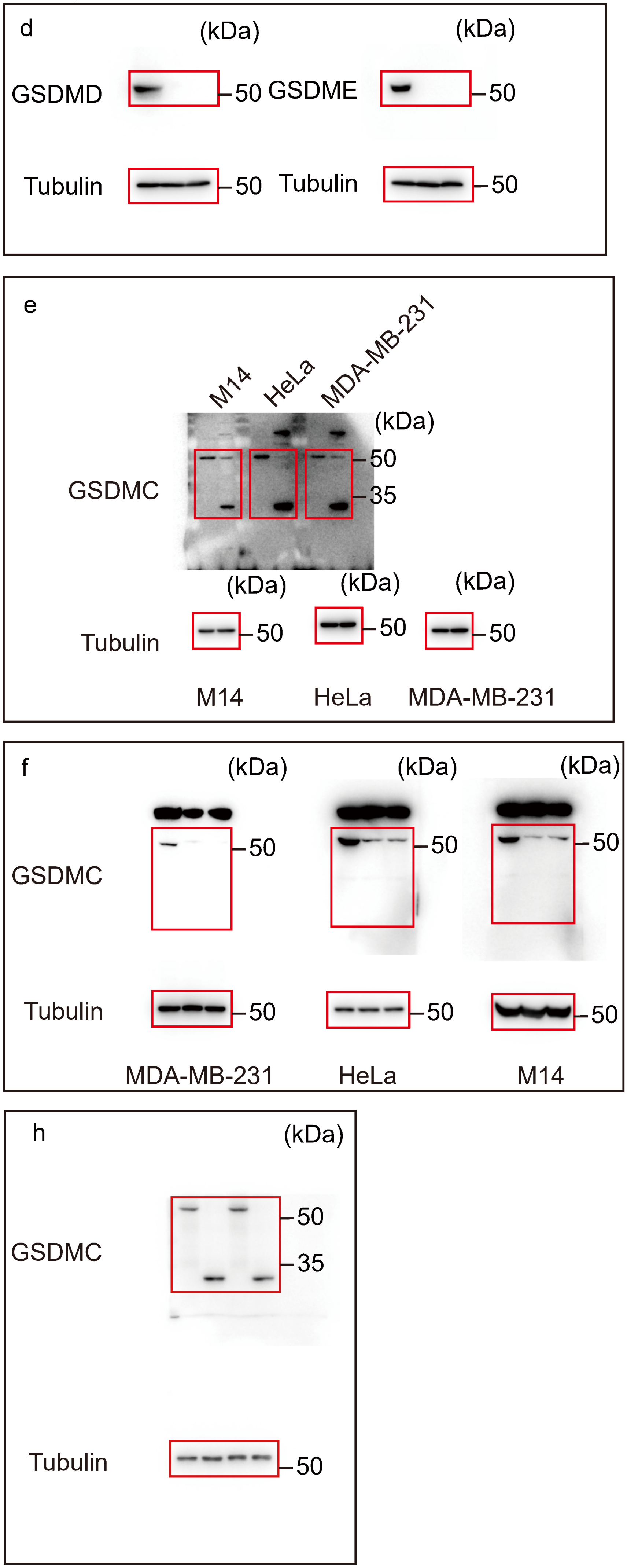
d
(kDa)
GSDMD
50
GSDME
(kDa)
50
Tubulin
50
Tubulin
50
e
M14
HeLa
MDA-MB-231
(kDa)
GSDMC
50
35
(kDa)
(kDa)
(kDa)
Tubulin
50
50
50
M14
HeLa
MDA-MB-231
f
(kDa)
(kDa)
(kDa)
GSDMC
50
50
50
Tubulin
50
50
50
MDA-MB-231
HeLa
M14
h
(kDa)
GSDMC
50
35
Tubulin
50

Fig.S2

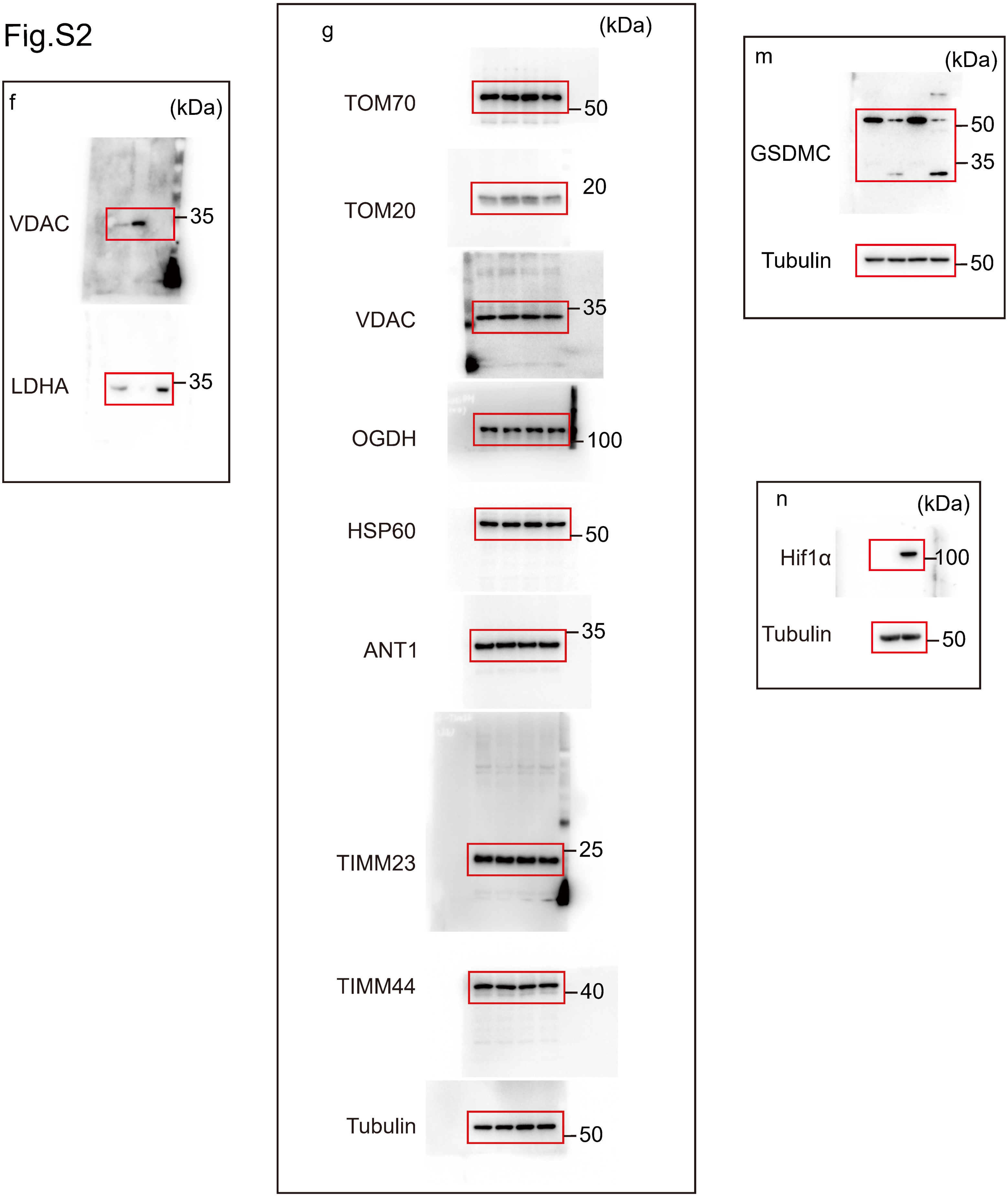

f
(kDa)
VDAC
35
LDHA
35
g
(kDa)
TOM70
50
TOM20
20
VDAC
35
OGDH
100
HSP60
50
ANT1
35
TIMM23
25
TIMM44
40
Tubulin
50
m
(kDa)
GSDMC
50
35
Tubulin
50
n
(kDa)
Hif1α
100
Tubulin
50

Fig.S3

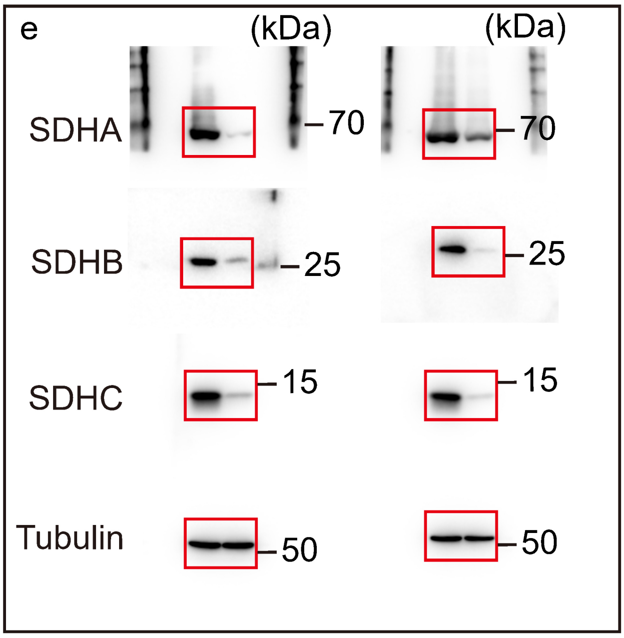

e
(kDa)
(kDa)
SDHA
70
70
SDHB
25
25
SDHC
15
15
Tubulin
50
50

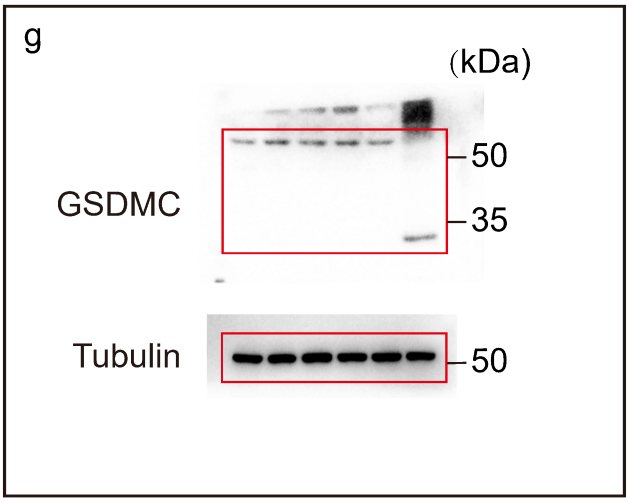

g
(kDa)
GSDMC
50
35
Tubulin
50

Fig.S4-1

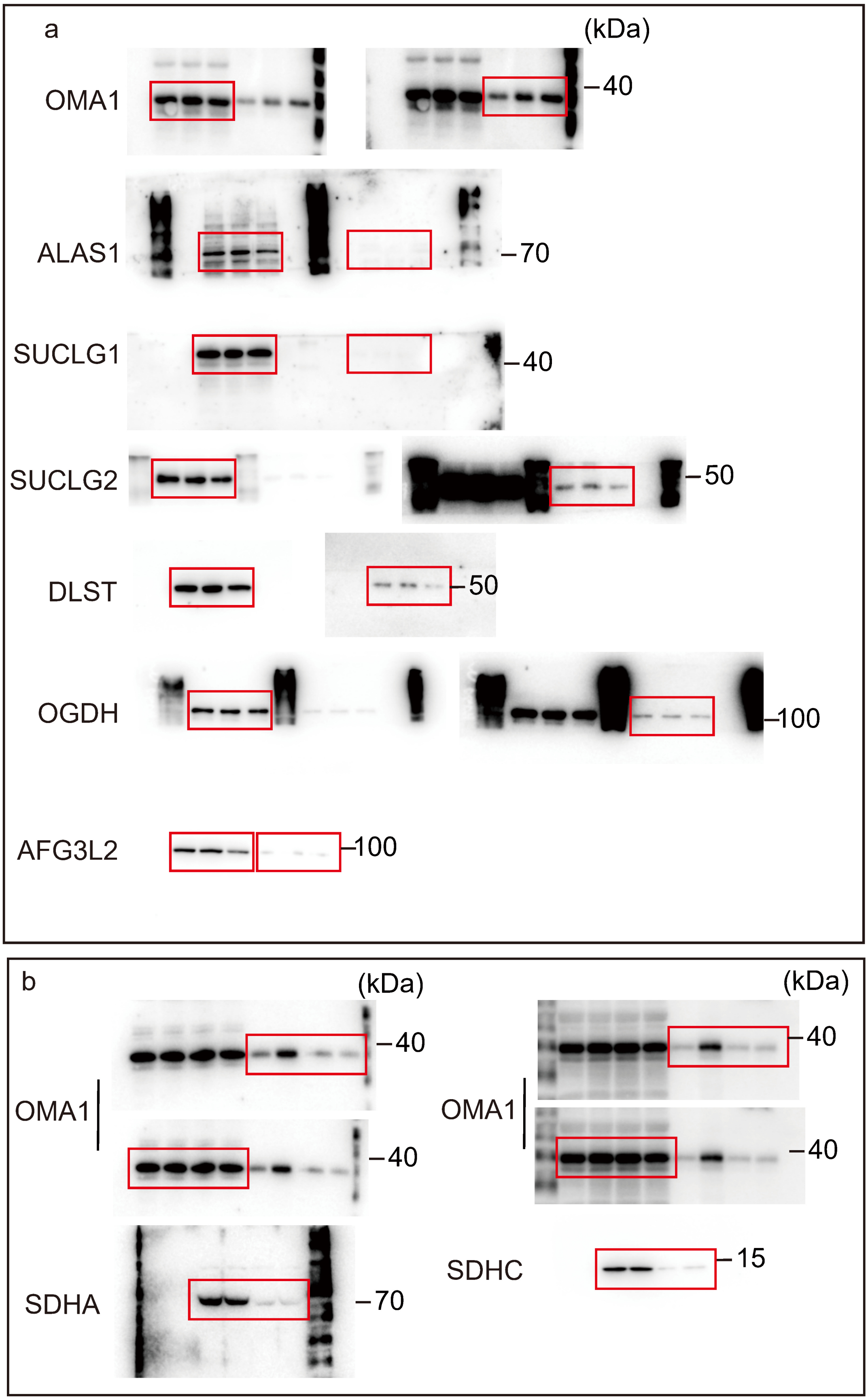
a
(kDa)
OMA1
40
ALAS1
70
SUCLG1
40
SUCLG2
50
DLST
50
OGDH
100
AFG3L2
100
b
(kDa)
OMA1
40
40
SDHA
70
(kDa)
OMA1
40
40
SDHC
15

Fig.S4-2

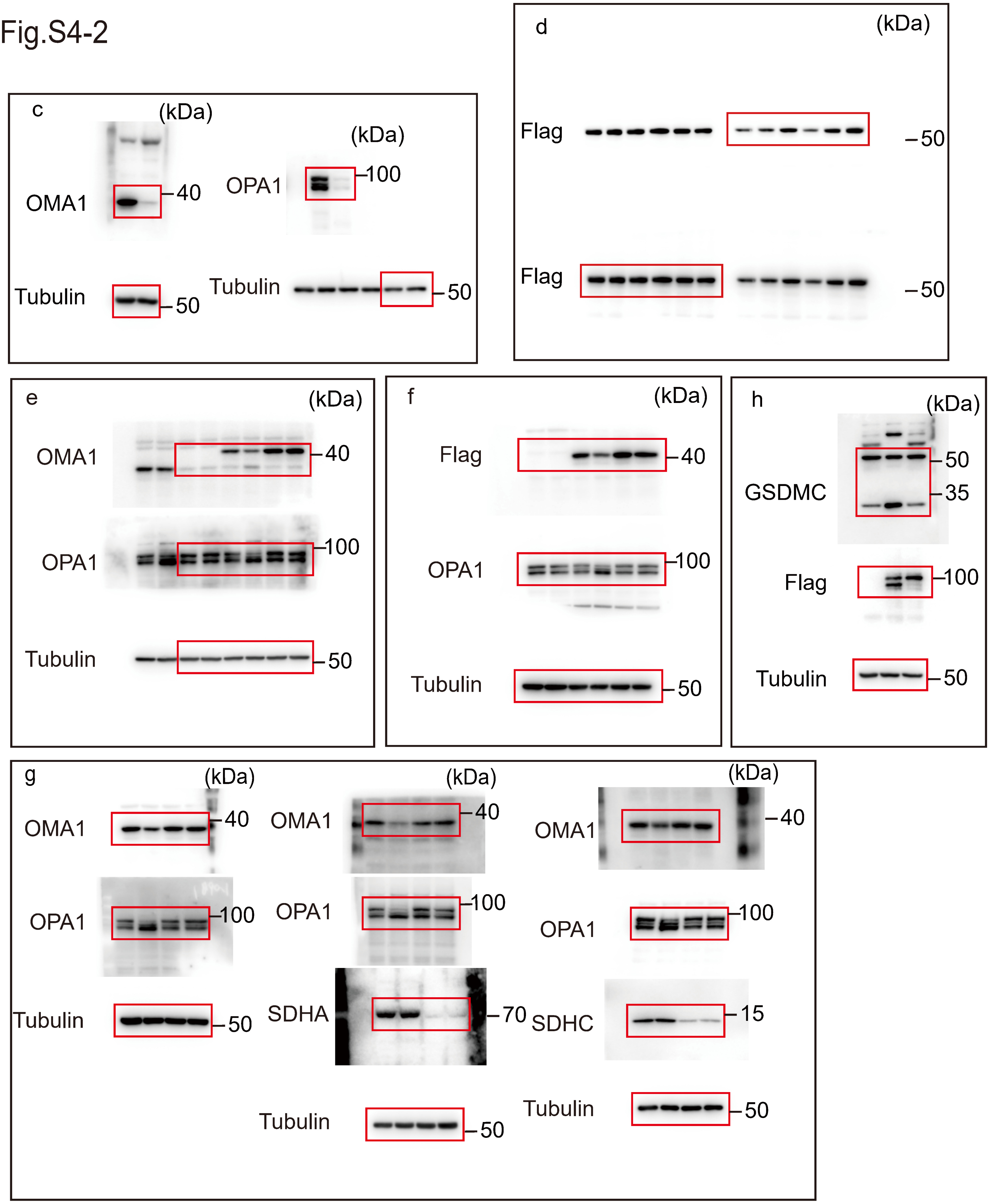
c
(kDa)
OMA1
40
OPA1
100
Tubulin
50
Tubulin
50
d
(kDa)
Flag
50
Flag
50
e
(kDa)
OMA1
40
OPA1
100
Tubulin
50
f
(kDa)
Flag
40
OPA1
100
Tubulin
50
h
(kDa)
GSDMC
50
35
Flag
100
Tubulin
50
g
(kDa)
OMA1
40
OPA1
100
Tubulin
50
OMA1
40
OPA1
100
SDHA
70
Tubulin
50
OMA1
40
OPA1
100
SDHC
15
Tubulin
50

Fig.S4-3

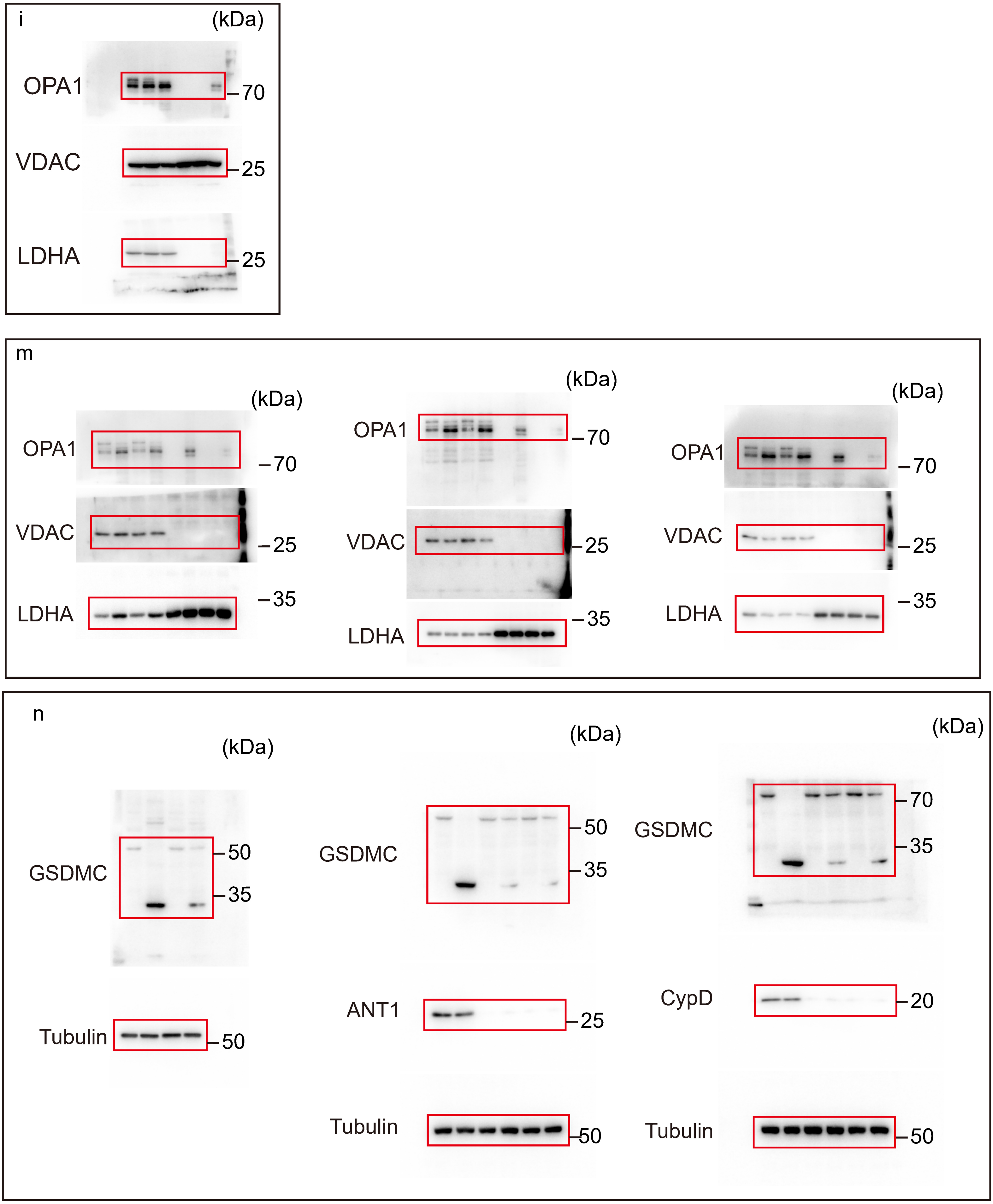
i
(kDa)
OPA1
70
VDAC
25
LDHA
25
m
(kDa)
OPA1
70
VDAC
25
LDHA
35
(kDa)
OPA1
70
VDAC
25
LDHA
35
(kDa)
OPA1
70
VDAC
25
LDHA
35
n
(kDa)
GSDMC
50
35
Tubulin
50
(kDa)
GSDMC
50
35
ANT1
25
Tubulin
50
(kDa)
GSDMC
70
35
CypD
20
Tubulin
50

Fig.S5-1

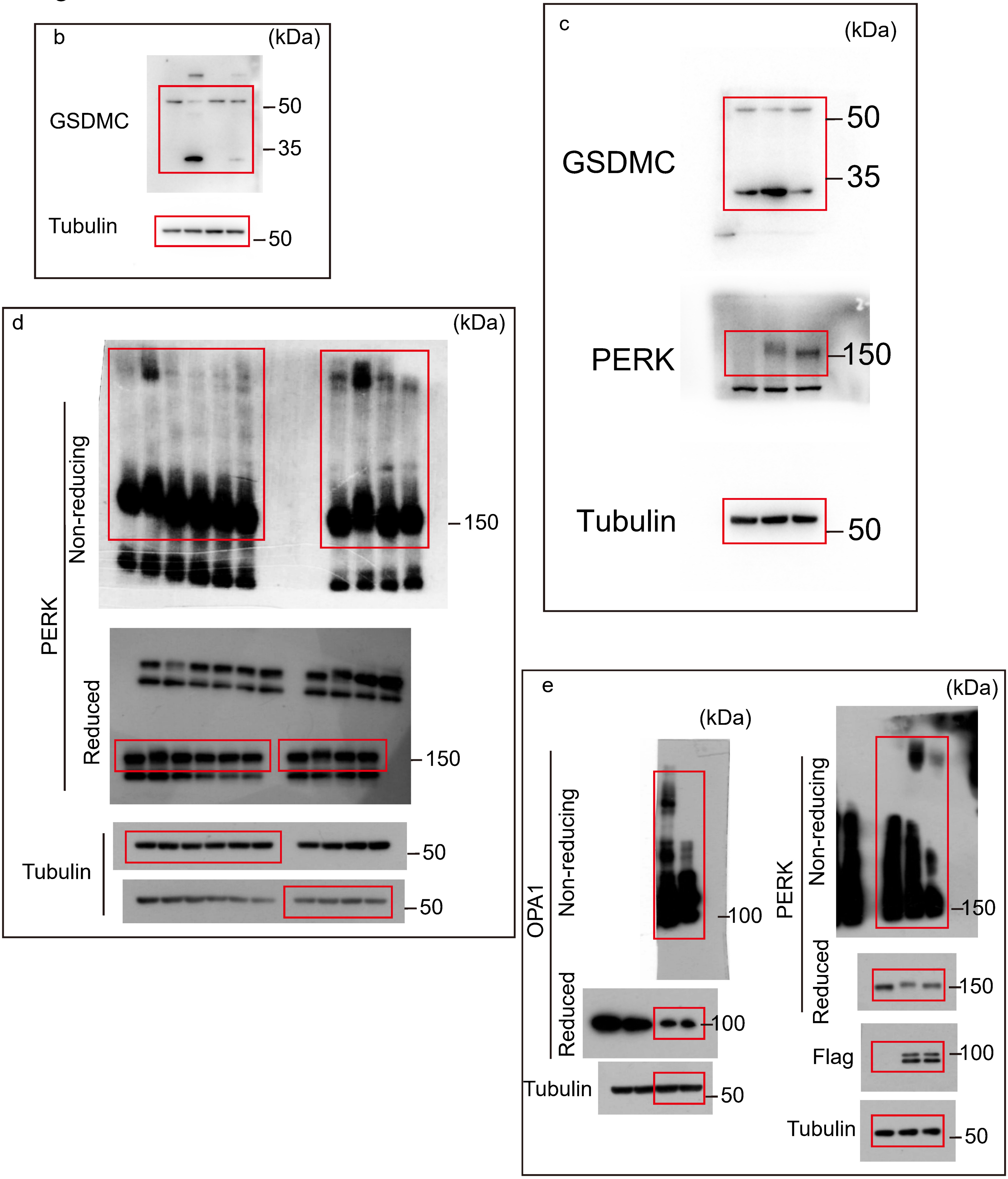
b
(kDa)
GSDMC
50
35
Tubulin
50
c
(kDa)
GSDMC
50
35
PERK
150
Tubulin
50
d
(kDa)
PERK
Non-reducing
150
Reduced
150
Tubulin
50
50
e
(kDa)
(kDa)
OPA1
Non-reducing
100
Reduced
100
Tubulin
50
PERK
Non-reducing
150
Reduced
150
Flag
100
Tubulin
50

Fig.S5-2

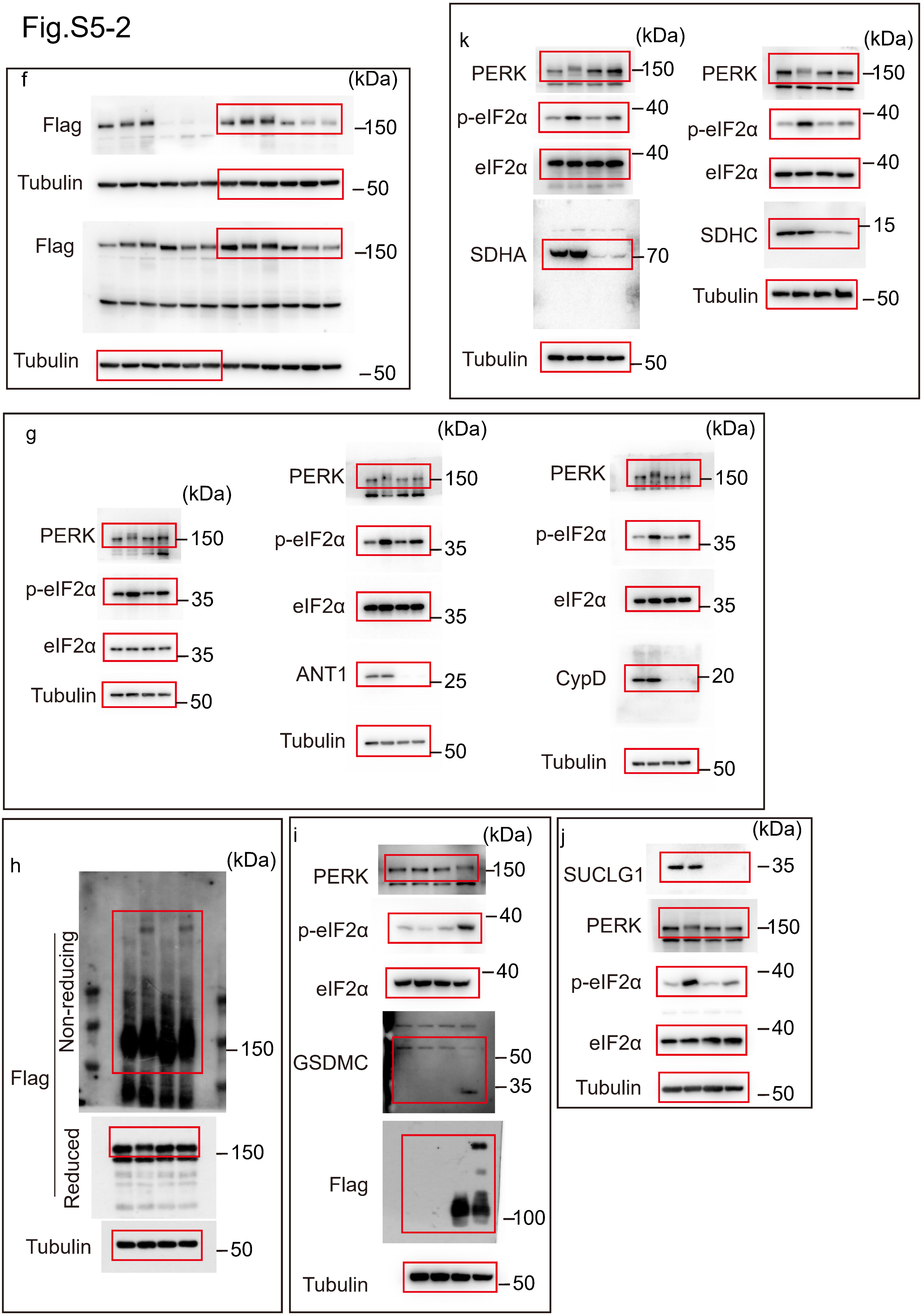

f
(kDa)
Flag
150
Tubulin
50
Flag
150
Tubulin
50
k
(kDa)
PERK
150
p-eIF2α
40
eIF2α
40
SDHA
70
Tubulin
50
(kDa)
PERK
150
p-eIF2α
40
eIF2α
40
SDHC
15
Tubulin
50
g
(kDa)
PERK
150
p-eIF2α
35
eIF2α
35
Tubulin
50
(kDa)
PERK
150
p-eIF2α
35
eIF2α
35
ANT1
25
Tubulin
50
(kDa)
PERK
150
p-eIF2α
35
eIF2α
35
CypD
20
Tubulin
50
h
(kDa)
Non-reducing
Flag
150
Reduced
150
Tubulin
50
i
(kDa)
PERK
150
p-eIF2α
40
eIF2α
40
GSDMC
50
35
Flag
100
Tubulin
50
j
(kDa)
SUCLG1
35
PERK
150
p-eIF2α
40
eIF2α
40
Tubulin
50

Fig.S5-3

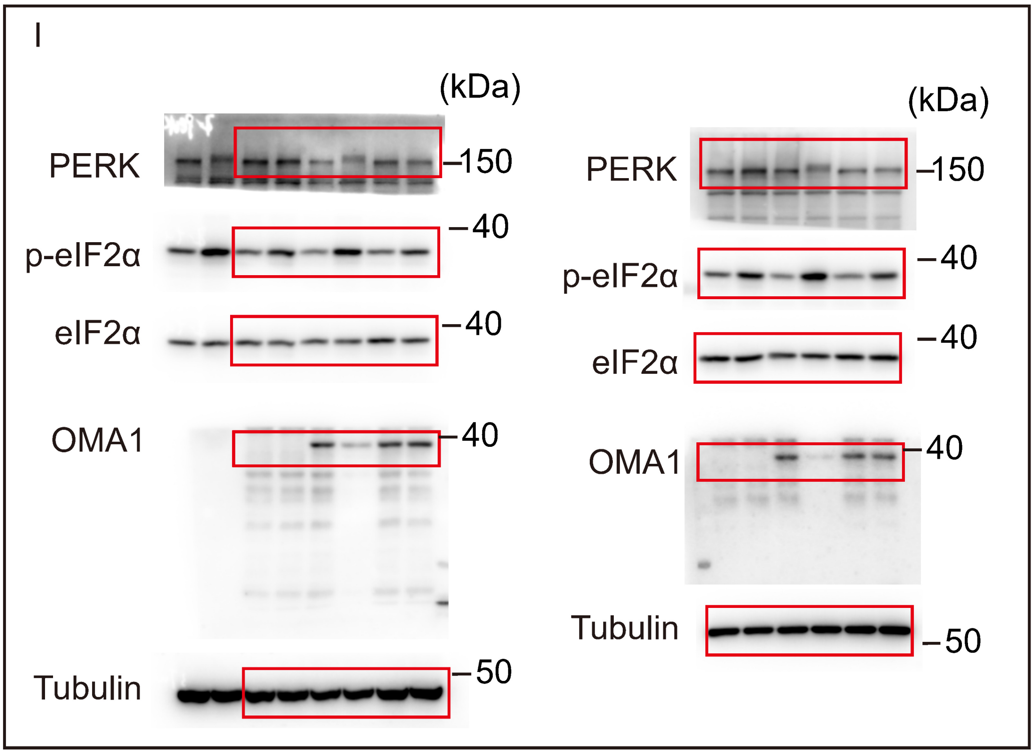
l
(kDa)
PERK
150
p-eIF2α
40
eIF2α
40
OMA1
40
Tubulin
50
(kDa)
PERK
150
p-eIF2α
40
eIF2α
40
OMA1
40
Tubulin
50

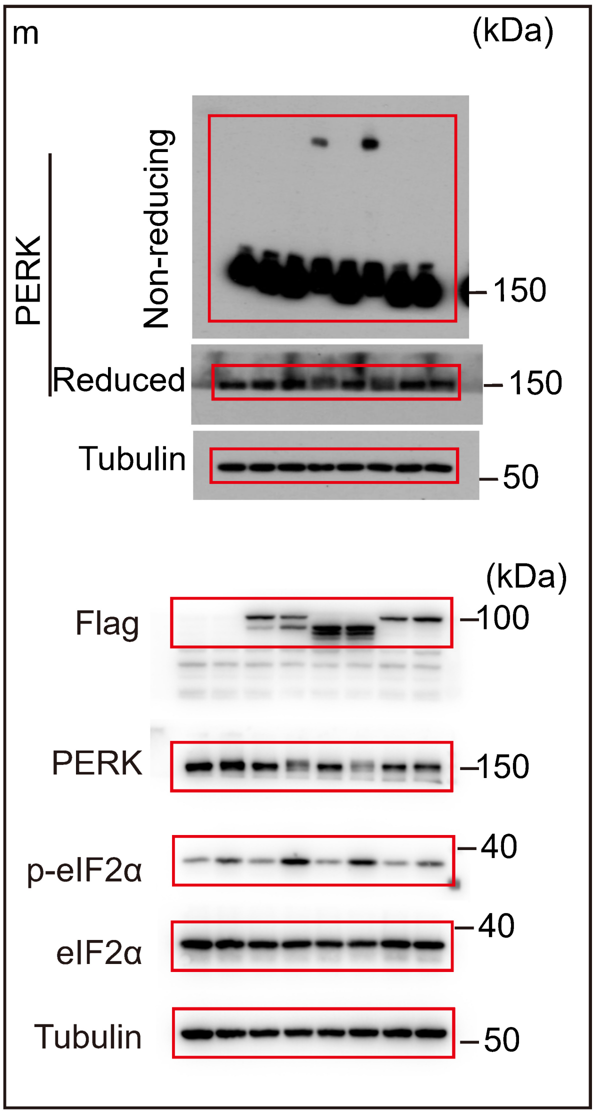
m
(kDa)
PERK
Non-reducing
150
Reduced
150
Tubulin
50
(kDa)
Flag
100
PERK
150
p-eIF2α
40
eIF2α
40
Tubulin
50

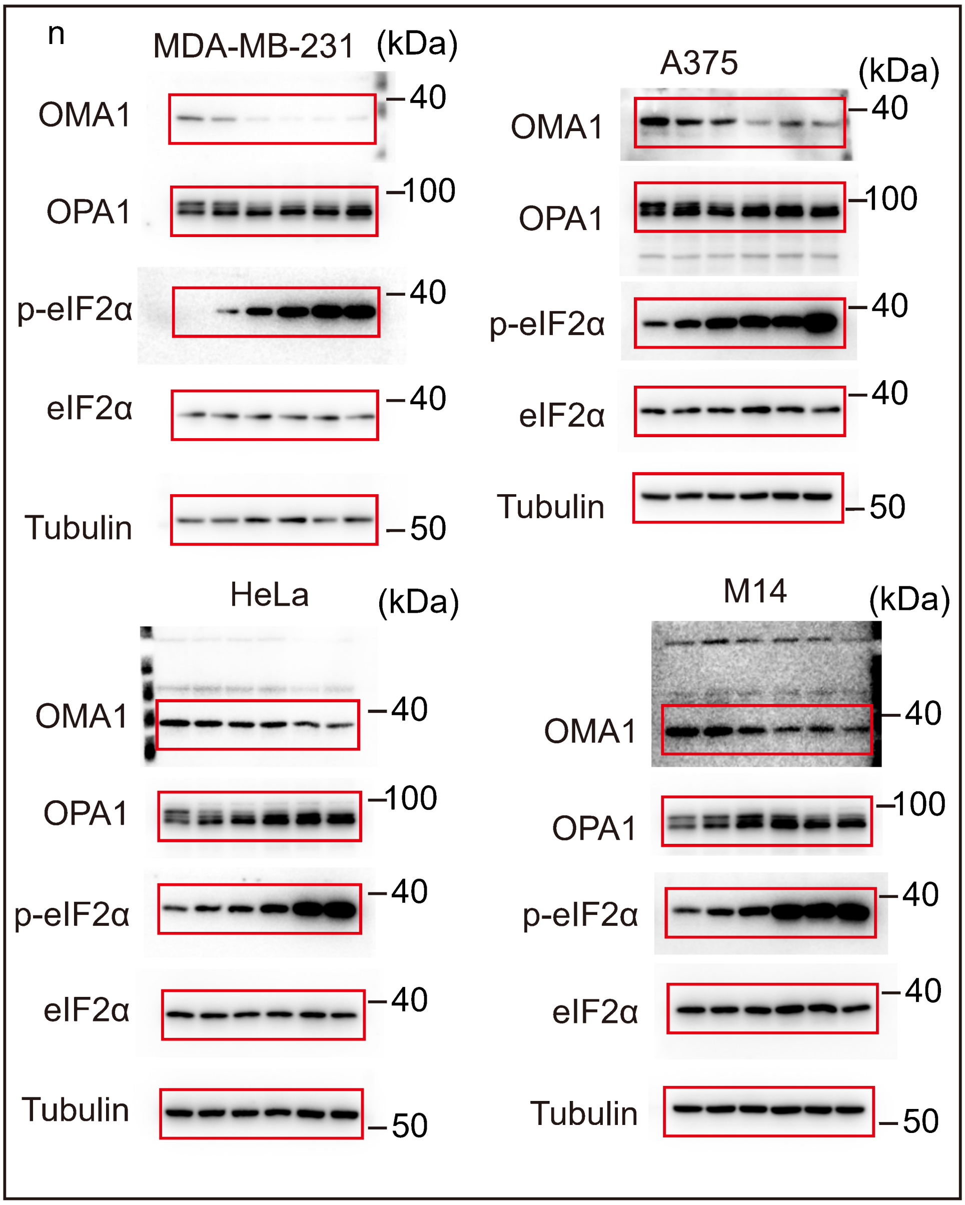
n
MDA-MB-231
(kDa)
OMA1
40
OPA1
100
p-eIF2α
40
eIF2α
40
Tubulin
50
A375
(kDa)
OMA1
40
OPA1
100
p-eIF2α
40
eIF2α
40
Tubulin
50
HeLa
(kDa)
OMA1
40
OPA1
100
p-eIF2α
40
eIF2α
40
Tubulin
50
M14
(kDa)
OMA1
40
OPA1
100
p-eIF2α
40
eIF2α
40
Tubulin
50

Fig.S6-1

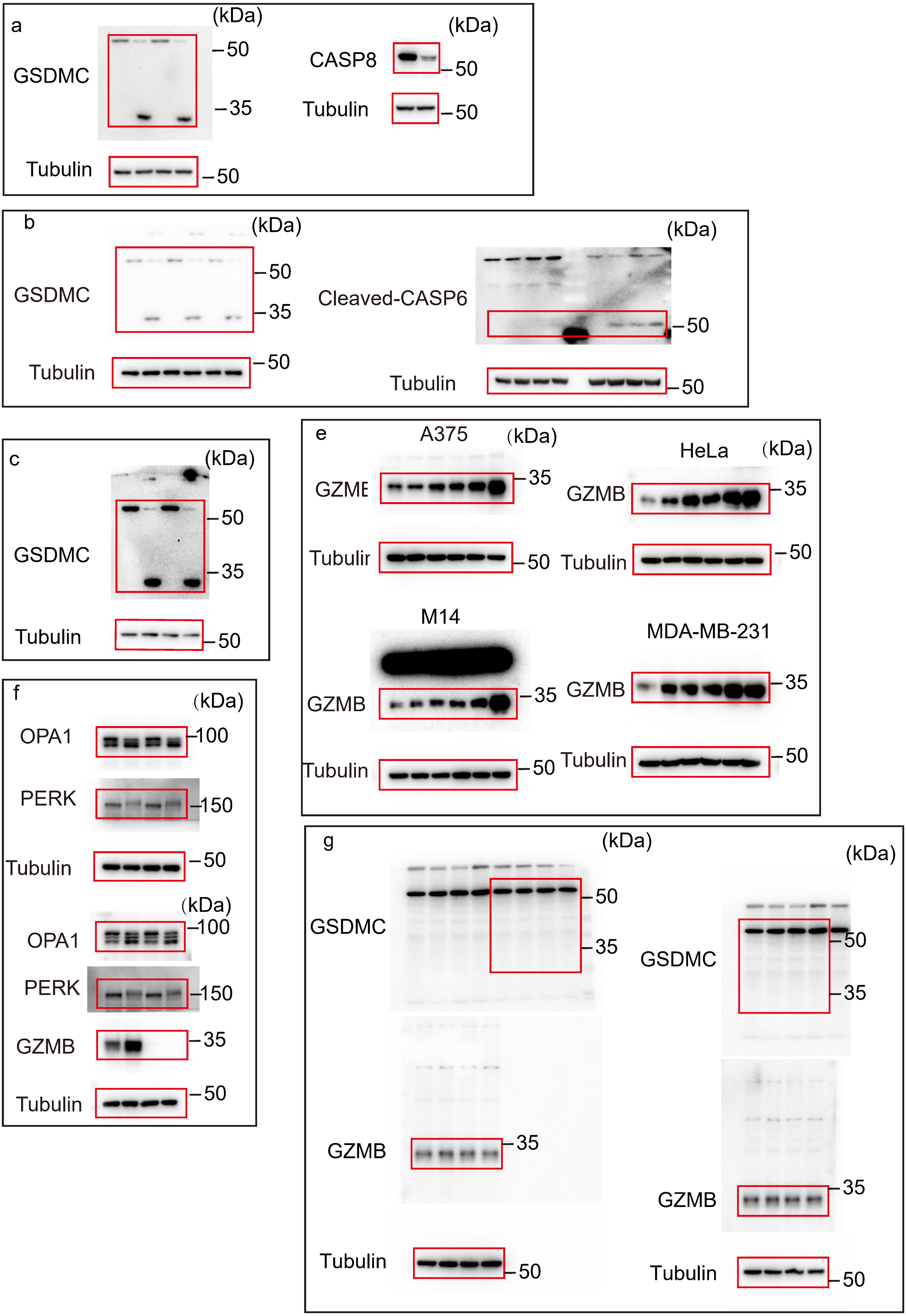
a
GSDMC
Tubulin
CASP8
Tubulin
(kDa)
50
35
b
GSDMC
Tubulin
Cleaved-CASP6
Tubulin
c
GSDMC
Tubulin
e
A375
GZMB
Tubulin
HeLa
M14
MDA-MB-231
f
OPA1
PERK
Tubulin
GZMB
100
150
g
GSDMC
GZMB
Tubulin

Fig.S6-2

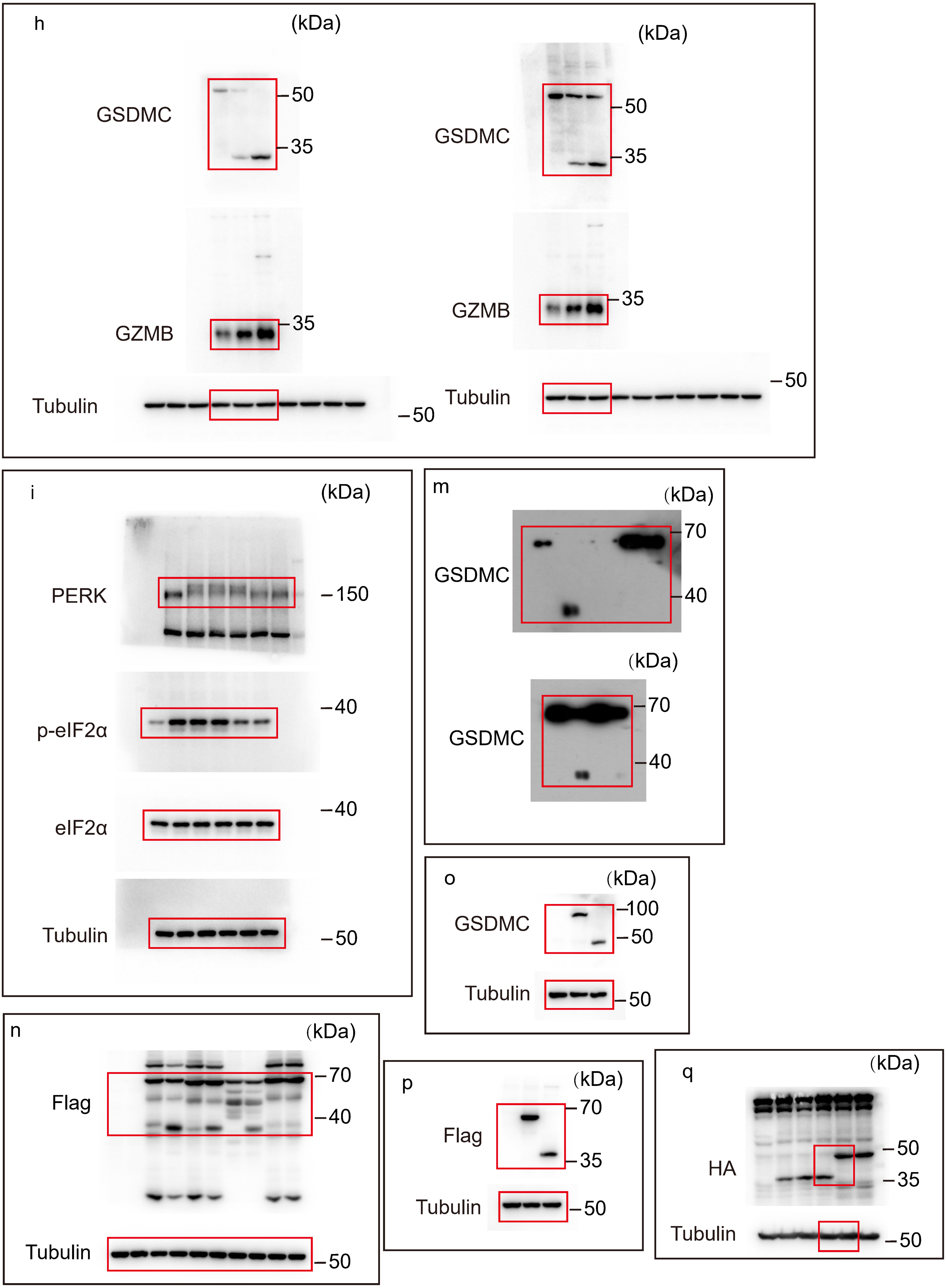

h
(kDa)
GSDMC
50
35
GZMB
35
Tubulin
50
(kDa)
GSDMC
50
35
GZMB
35
Tubulin
50
i
(kDa)
PERK
150
p-eIF2α
40
eIF2α
40
Tubulin
50
m
(kDa)
GSDMC
70
40
(kDa)
GSDMC
70
40
o
(kDa)
GSDMC
100
50
Tubulin
50
n
(kDa)
Flag
70
40
Tubulin
50
p
(kDa)
Flag
70
35
Tubulin
50
q
(kDa)
HA
50
35
Tubulin
50

Fig.S7-1

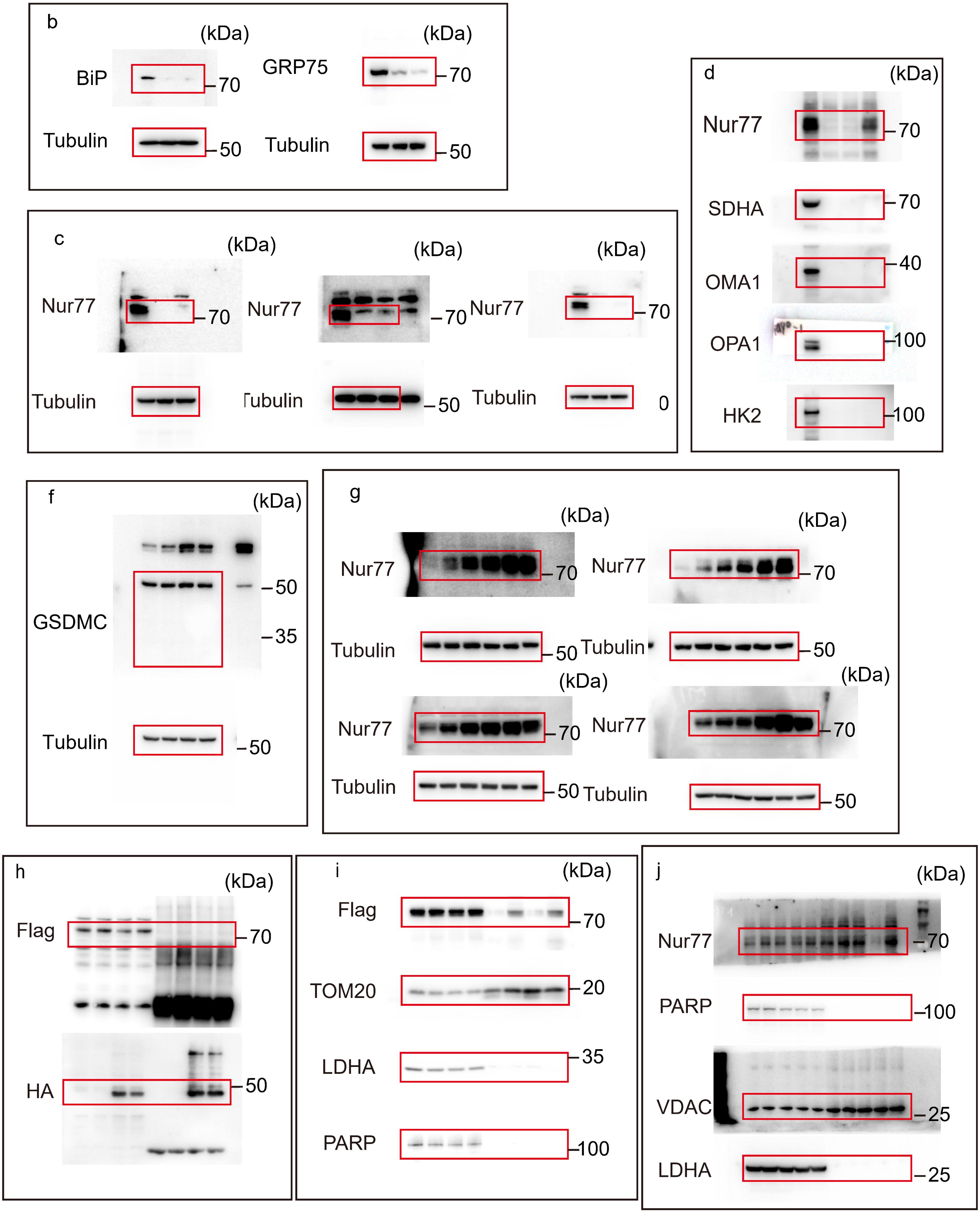

b
(kDa)
BiP
70
Tubulin
50
GRP75
70
Tubulin
50
c
(kDa)
Nur77
70
Tubulin
Nur77
70
Tubulin
50
Nur77
70
Tubulin
d
(kDa)
Nur77
70
SDHA
70
OMA1
40
OPA1
100
HK2
100
f
(kDa)
GSDMC
50
35
Tubulin
50
g
(kDa)
Nur77
70
Tubulin
50
Nur77
70
Tubulin
50
Nur77
70
Tubulin
50
Nur77
70
Tubulin
50
h
(kDa)
Flag
70
HA
50
i
(kDa)
Flag
70
TOM20
20
LDHA
35
PARP
100
j
(kDa)
Nur77
70
PARP
100
VDAC
25
LDHA
25

Fig.S7-2

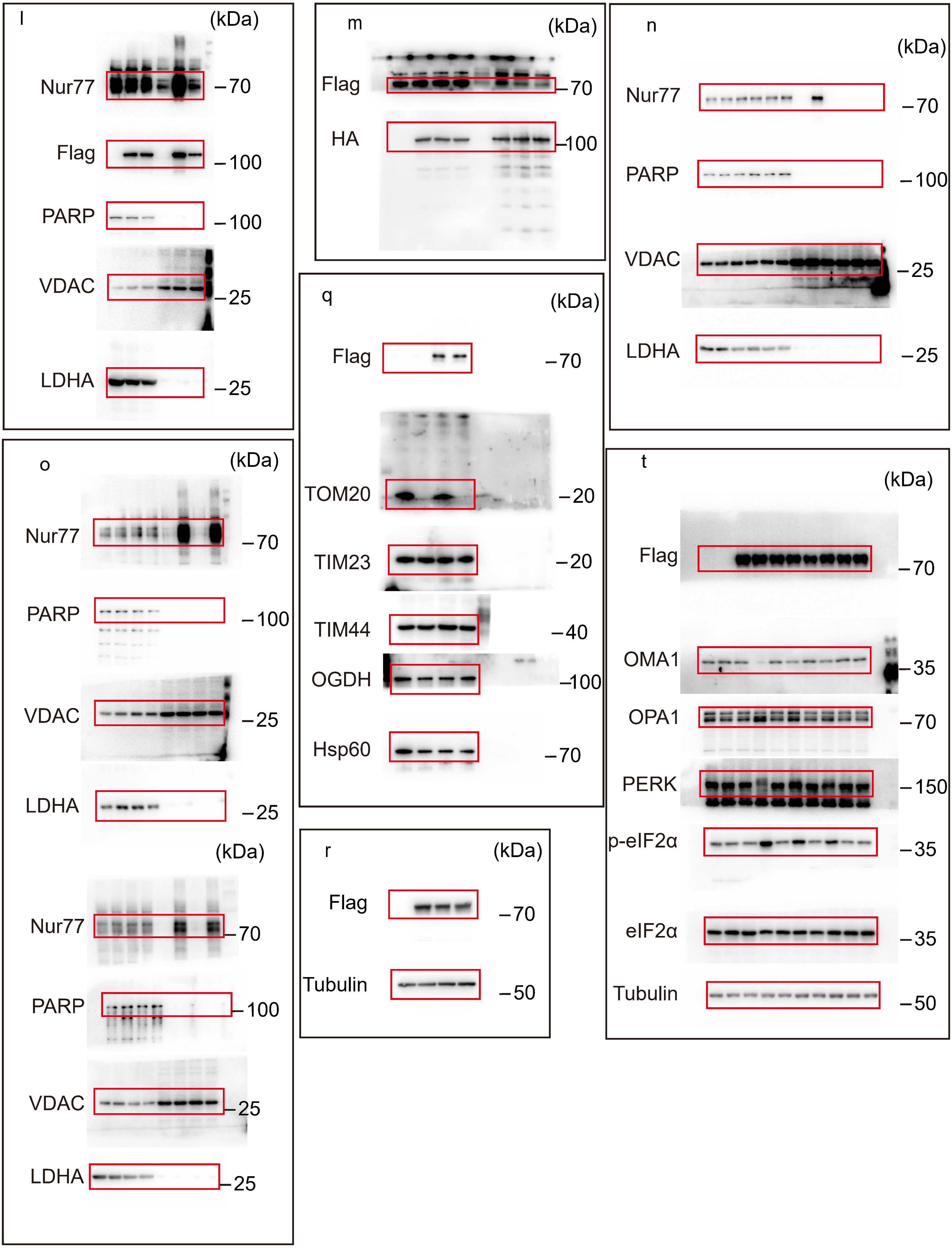

l
(kDa)
Nur77
70
Flag
100
PARP
100
VDAC
25
LDHA
25
m
(kDa)
Flag
70
HA
100
n
(kDa)
Nur77
70
PARP
100
VDAC
25
LDHA
25
o
(kDa)
Nur77
70
PARP
100
VDAC
25
LDHA
25
(kDa)
Nur77
70
PARP
100
VDAC
25
LDHA
25
q
(kDa)
Flag
70
TOM20
20
TIM23
20
TIM44
40
OGDH
100
Hsp60
70
r
(kDa)
Flag
70
Tubulin
50
t
(kDa)
Flag
70
OMA1
35
OPA1
70
PERK
150
p-eIF2α
35
eIF2α
35
Tubulin
50

Fig.S7-3

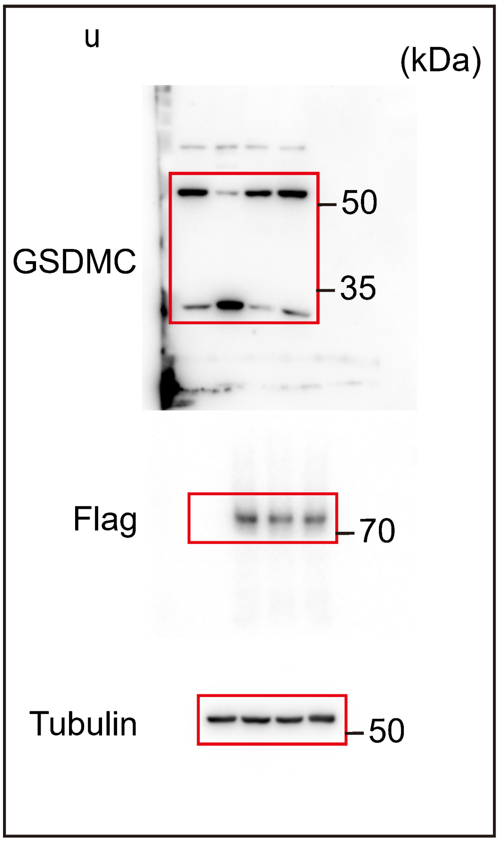
u
(kDa)
GSDMC
50
35
Flag
70
Tubulin
50

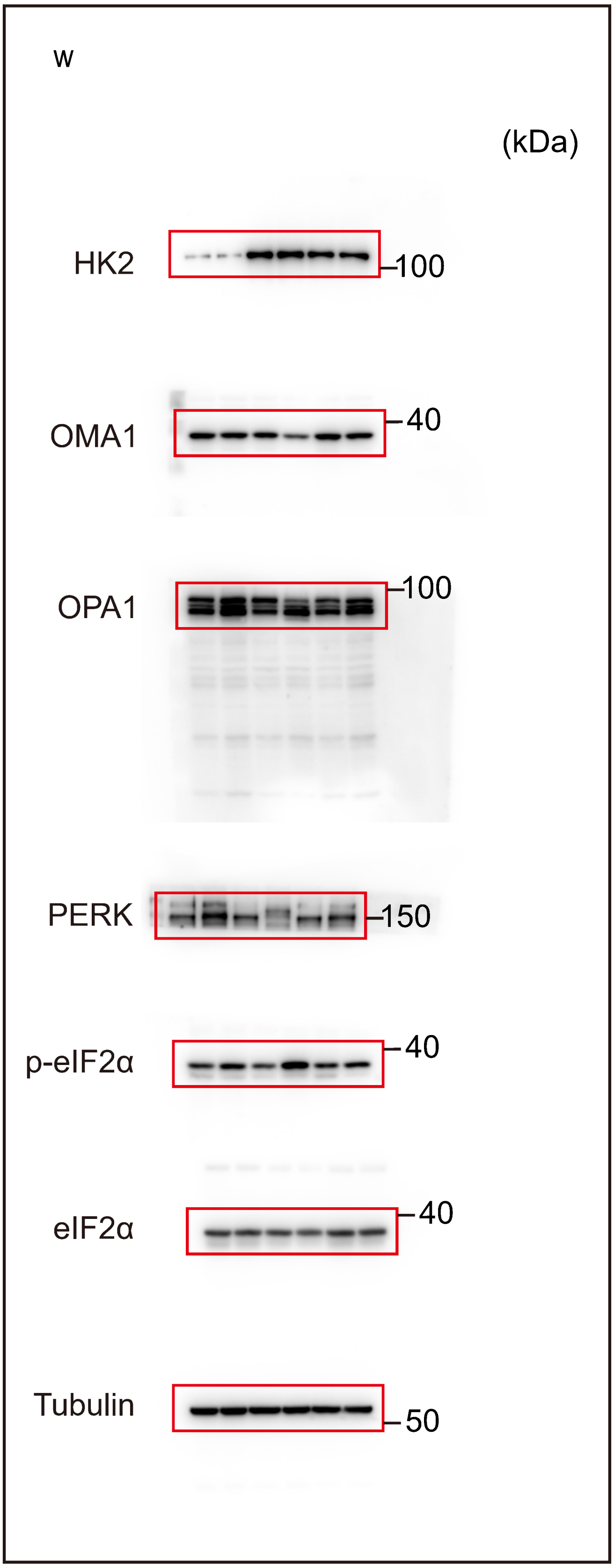
w
(kDa)
HK2
100
OMA1
40
OPA1
100
PERK
150
p-eIF2α
40
eIF2α
40
Tubulin
50

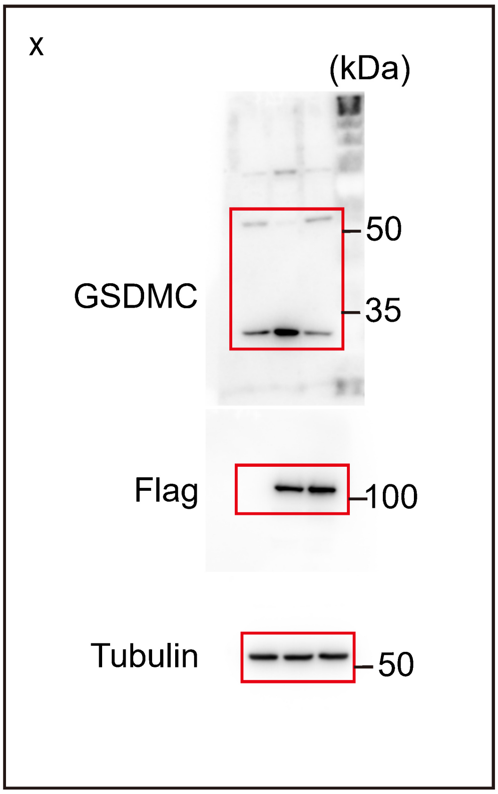
x
(kDa)
GSDMC
50
35
Flag
100
Tubulin
50

Fig.S8

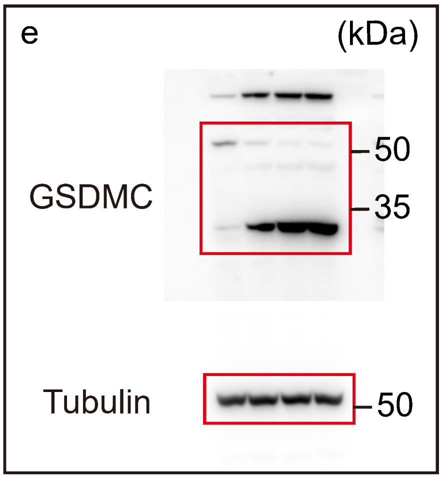

Supplement: Supplementary file 1 — Original uncropped images of western blot [file 41392_2025_2528_MOESM1_ESM.pdf]
