# Supplementary material for: Disruption of heme homeostasis by nuclear receptor Nur77 induces pyroptosis through granzyme B-dependent GSDMC cleavage
Source: Signal Transduct Target Ther. 2025 Dec 17;10:413. doi: 10.1038/s41392-025-02528-w (PMC12711971; doi:10.1038/s41392-025-02528-w)
Supplement: Supplementary file 2 — Supplementary data.pdf [file 41392_2025_2528_MOESM2_ESM.docx]

Supplementary Materials for

**Disruption of heme homeostasis by nuclear receptor Nur77 induces pyroptosis through granzyme B-dependent GSDMC cleavage**

Liu-zheng Wu^#,1^, Ya-ying Huang^#,1^, Hai-jing Hu^#,1^, Wen-bin Hong^#,2^, Han Yan^#,3^, Yuan-li Ai^1^, Xiang-yu Mi^1^, De-yi Feng^1^, Jian-yi Guo^1^, Yang Ding^3^, Zai-jun Liu^1^, Bo Zhou^1^, Li Xiao^1^, Tianwei Lin^2^, Fu-nan Li*^,3^, Xue-qin Chen*^2^, Hang-zi Chen*^,1^, Qiao Wu*^,1^

Correspondence to: qiaow@xmu.edu.cn; chenhz@xmu.edu.cn; xqchen@xmu.edu.cn

fnlee5@xmu.edu.cn

**This PDF file includes:**

Materials and Methods

Figures. S1 to S8

Tables S1

**Other Supplementary Materials for this manuscript include the following:**

Original images of Western blots

**Materials and Methods**

**Chemical reagents, various inhibitors and antibodies**

1. **Chemical reagents**

| Dimethyl α-ketoglutarate | Sigma Aldrich | Cat# 349631 |
| --- | --- | --- |
| Hemin | Sigma Aldrich | Cat# 51280 |
| Dimethyl succinate  DAPI | Sigma Aldrich  Sigma Aldrich | Cat# [V900547](https://www.sigmaaldrich.cn/CN/en/product/vetec/v900547)  Cat# D9542 |
| MitoSox | Thermo Fisher Scientific | Cat# M36008 |
| Pierce™ NeutrAvidin™ | Thermo Fisher Scientific | Cat# 29201 |
| Digitonin | Sangon Biotech | Cat# A60115 |
| Strep-Tactin Sepharose | Nuptec | Cat# NRPB42L-20 |
| ZnMP | Frontier Scientific | Cat# M40628 |
| CIAP | ACMEC | Cat# 9001-78-9 |
| Cocktail | MedChemExpress | Cat# HY-K0010 |
| Tunicamycin | MedChemExpress | Cat# HY-A0098 |
| Thapsigargin | MedChemExpress | Cat# HY-13433 |
| CCT020312 | MedChemExpress | Cat# HY-119240 |
| MK-28 | MedChemExpress | Cat# HY-137207 |
| MCA-AFRATDHG-Lys(DNP) | LifeTein LLC | Cat# LT119950 |

1. **Inhibitors**

| Ferrostatin-1 | Sigma Aldrich | Cat# SML0583 |
| --- | --- | --- |
| (Z-LL)_2_ Ketone | Sigma Aldrich | Cat# SML1442 |
| Rotenone  Antimycin A | MedChemExpress  MedChemExpress | Cat# HY-B1756  Cat# HY-105755 |
| Oligomycin | MedChemExpress | Cat# HY-16589 |
| Biotin-azide | MedChemExpress | Cat# HY-129832 |
| Dimethyl-malonate | MedChemExpress | Cat# HY-Y1787 |
| ISRIB | MedChemExpress | Cat# HY-12495 |
| Diethyl butylmalonate | MedChemExpress | Cat# HY-44178 |
| CQ | MedChemExpress | Cat# HY-17589A |
| 2-Thenoyltrifluoroacetone | MedChemExpress | Cat# HY-D0190 |
| Liproxstatin-1  Necrosulfonamide  Necrostatin-1  TTM  SA  Pepstatin A  MG-101  E64D  Mito-Q  α-VE  Mdivi-1  Liensinine  MFI8  Z-VAD  PF429242  3,4-Dichloroisocoumarin  llomastat  Batimastat  L-685,458  Trametinib | MedChemExpress  MedChemExpress  MedChemExpress  MedChemExpress  MedChemExpress  MedChemExpress  MedChemExpress  MedChemExpress  MedChemExpress  MedChemExpress  MedChemExpress  MedChemExpress  MedChemExpress  ApexBio  TargetMol  CSNpharm.  CSNpharm.  CSNpharm.  Aladdin  Aladdin | Cat# HY-12726  Cat# HY-100573  Cat# HY-15760  Cat# HY-W076067  Cat# HY-W010184  Cat# HY-18234  Cat# HY-18964  Cat# HY-100229  Cat# HY-100116A  Cat# HY-N0683  Cat# HY-15886  Cat# HY-N0484  Cat# HY-150031  Cat# A1902  Cat# T12437  Cat# CSN24042  Cat# CSN11187  Cat# CSN17343  Cat# L275059  Cat# T127461 |

1. **Antibodies**

| Goat anti-rabbit  secondary antibody  Goat anti-mouse secondary antibody | Thermo Fisher Scientific  Thermo Fisher Scientific | Cat# 31210  Cat# 31160 |
| --- | --- | --- |
| Anti-tubulin | Sigma Aldrich | Cat# T-4026 |
| Anti-HA | Sigma Aldrich | Cat# H-9658 |
| Anti-Flag | Sigma Aldrich | Cat# F-1804 |
| Anti-BiP | Cell Signaling Technology | Cat# 3177 |
| Anti-LDHA | Cell Signaling Technology | Cat# 3582T |
| Anti-PARP | Cell Signaling Technology | Cat# 9532S |
| Anti-Nur77 | Cell Signaling Technology | Cat# 3960S |
| Anti-Tom20 | Cell Signaling Technology | Cat# 42406S |
| Anti-Hsp60 | Cell Signaling Technology | Cat# 12165S |
| Anti-VDAC | Cell Signaling Technology | Cat# 4866 |
| Anti-GRP75  Anti-ALAS1 | Proteintech  Proteintech | Cat# 14887-1-AP  Cat#16200-1-AP |
| Anti-OGDH  Anti-OMA1  Anti-TIMM44  Anti-TIMM23  Anti-SDHA  Anti-SDHB  Anti-AFG3L2  Anti-SDHC  Anti-PERK  Anti-p-eIF2α  Anti-eIF2α  Anti-SUCLG1  Anti-GSDMC  Anti-GSDME  Anti-GSDMD  Anti-OPA1  Anti-HK2  Anti-GZMB  Anti-Nur77 | Proteintech  Proteintech  Proteintech  Proteintech  Proteintech  Proteintech  ABclonal  ABclonal  ABclonal  ABclonal  ABclonal  ABclonal  ABclonal  Abcam  Abcam  BD Biosciences  PTM BIO  Santa Cruz  Aviva Systems Biology | Cat# 15212-1-AP  Cat# 17116-1-AP  Cat# 13859-1-AP  Cat# 11123-1-AP  Cat#14865-1-AP  Cat#10620-1-AP  Cat# A15393  Cat# A22280  Cat# A18196  Cat# AP0692  Cat# A21221  Cat# A15345  Cat# A14550  Cat# ab215191  Cat# ab210070  Cat# 612607  Cat# PTM-5371  Cat# sc-8022  Cat#ARP31941 |

**Cell culture and transfection**

Except for the human kidney clear cell carcinoma cell line 786-O, which was maintained in RPMI 1640 medium. All the cell lines, including the melanoma cell lines (A375, G361, IgR3, M14, MM200, ME4405, and Mel-RM), and other cell lines, including the human non-small cell lung cancer cell line A549, the human cervical carcinoma cell line HeLa, the human breast cancer cell line MDA-MB-231, the human pancreatic cancer cell lines MIA-PaCa-2 and PANC-1, the human colon cancer cell line SW620, the human osteosarcoma cell line U-2 OS and the human embryonic kidney cell line HEK293T, were cultured in Dulbecco’s modified Eagle’s medium (Sigma) supplemented with 10% FBS, penicillin (100 IU) and streptomycin (100 μg/mL) (Bio Basic Inc., Shanghai, China) at 37 °C in a humidified incubator containing 5% CO_2_. During DdBIC treatment, the concentration of FBS was reduced to 0.5%. The cells were pretreated with the inhibitors for 2 h before DdBIC treatment. Lipofectamine 2000 (Invitrogen) or polyethyleneimine (PEI, Yeasen) transfection reagents were used for plasmid transfection. PEI was used for the HEK293T cells, and Lipofectamine 2000 was used for the other cell lines.

**Plasmid construction**

The cDNA sequences of Nur77, SDHA, SDHC, OMA1, OPA1, S-OPA1, EIF2S1, HK2, GSDMC and GZMB were separately cloned and inserted into a pLenti (Addgene 22255) vector via PCR/restriction digestion-based cloning. The OMA1 (12CS and E328Q), PERK (K618R and T982A), EIF2S1 (S51A), GZMB (S183A), GSDMC (D231/232/233/240/276A and Δ260-280), HK2 (Δ1-16), OPA1 (2CS and Δ194-195) and Nur77 (3mt, 4mt and 5mt) genes were generated via the QuikChange method. The cDNA sequences of GFP, GSDMC and GZMB were inserted into pLVX-EnCMV-V5-TurboID (Addgene) vectors. GSDMC^wt^ and GSDMC^1–276^ were subsequently cloned and inserted into a pBOB-HBD*-HA vector. All the plasmids were purified via a TIANprep Midi plasmid kit (Tiangen, Cat# DP106) and verified by sequencing.

**Generation of the lentiviral system**

The lentiviral-based vectors pLKO.1 (Addgene) and pLL3.7 (Addgene) were used to express shRNAs in cells. The oligonucleotides were annealed and subcloned and inserted into the pLKO.1 or pL3.7 vector. To generate lentiviruses, HEK293T cells were transfected with both shRNA and packaging plasmids (psPAX2 (Addgene 12260) and pMD2.G (Addgene 14887)). The lentiviral supernatant was collected after transfection for 48 h, centrifuged at 6500 × g for 5 min or filtered through a 0.45-μm filter (Millipore). The cells were infected with the lentiviruses for 24 h with polybrene and screened with puromycin. Western blotting or real-time PCR was used to detect the knockdown efficiency of the target genes. The following oligonucleotide sequences were used.

| Scramble shRNA: | 5'-GCGCGCTTTGTAGGATTCG-3' |
| --- | --- |
| NMC shRNA | 5'-GCGCGATAGCGCTAATAATTT-3' |
| GSDMA shRNA1 | 5'-CTTACCAAGGCCTCCTAATTT-3' |
| GSDMA shRNA2 | 5'-CCTCGCCTCTGTGCTCTTTAT-3' |
| GSDMB shRNA1 | 5'-GCTGTATGTTGTTGTCTCTAT-3' |
| GSDMB shRNA2 | 5'-GAAGCCTTGTTGATGCTGATA-3' |
| GSDMC shRNA1 | 5'-CTGAGTGACTTCCAACACGAT-3' |
| GSDMC shRNA2 | 5'-GGTGGAGTAGAGACAGTAATG-3' |
| GSDMD shRNA1 | 5'-GCAGGAGCTTCCACTTCTA-3' |
| GSDMD shRNA2 | 5'-GCCATCTGAGCCAGAAGAAGA-3' |
| GSDME shRNA1 | 5'-GCGGAGAATTCTTAGCATA-3' |
| GSDME shRNA2 | 5'-GCAGCAAGCAGCTGTTTATGA-3' |
| SUCLG1 shRNA1 | 5'-TGGAATGGATCACGTAGACAT-3' |
| SUCLG1 shRNA2 | 5'-GCAACATAATTCAGGTCCAAA-3' |
| SDHA shRNA1 | 5'-GCATCTGCTAAAGTTTCAG-3' |
| SDHA shRNA2 | 5'-CAAGCTCTATGGAGACCTAA-3' |
| SDHC shRNA1 | 5'-GAGCGGTTCTGGAATAAGA-3' |
| SDHC shRNA2 | 5'-GCAGTTATTCTCTCTCCAT-3' |
| NDUFS4 shRNA1 | 5'-CCCTTATCCAACATGGTTCTA-3' |
| NDUFS4 shRNA2 | 5'-GAGGACTTCCACATGGAGATT-3' |
| RISP shRNA1 | 5'-CGAAATCAAGTTATCCGATAT-3' |
| RISP shRNA2 | 5'-GCTATGCATTTCCTCCCTACT-3' |
| COX4I1 shRNA1 | 5'-GCAACTCCATGCCTATTTACT-3' |
| COX4I1 shRNA2 | 5'-AGTCGAGTTGTATCGCATTAA-3' |
| ATP5PD shRNA1 | 5'-CCATTGCTAGTTCCCTGAAAT-3' |
| ATP5PD shRNA2 | 5'-GAAGAAGTTTAATGCGCTGAA-3' |
| OMA1 shRNA1 | 5'-GCAGCAGTCCCTAGTCTGTCA-3' |
| OMA1 shRNA2 | 5'-GCGGTTCCTCTCTTGTTGATG-3' |
| OPA1 shRNA1 | 5'-GCTGAACGCAGTATTGTTACA-3' |
| OPA1 shRNA2 | 5'-GCACACCAAGTGACTACAAGA-3' |
| PERK shRNA1 | 5'-GGAACGACCTGAAGCTATAAA-3' |
| PERK shRNA2 | 5'-GCATGGAAACAGTTCCTTTCA-3' |
| EIF2S1 shRNA1 | 5'-GCAGATATTGAAGTGGCTTGT-3' |
| EIF2S1 shRNA2 | 5'-GCCAGAGAATAGATCAGTATT-3' |
| HK2 shRNA1 | 5'-CCAAAGACATCTCAGACATTG-3' |
| HK2 shRNA2 | 5'-CACGATGAAATTGAACCTGGT-3' |
| Nur77 shRNA1 | 5'-GGGCATGGTGAAGGAAGTTGT-3' |
| Nur77 shRNA2 | 5'-GCCCTGTATCCAAGCCCAATA-3' |
| GZMB shRNA1 | 5'-CGAATCTGACTTACGCCATTA-3' |
| GZMB shRNA2 | 5'-GGTGCCAGCAACTGAATAAAT-3' |
| GRP75 shRNA1 | 5'-GCTCATGGGAAATTGTATTCT-3' |
| GRP75 shRNA2 | 5'-GGAACGAGTTGAAGCAGTTAA-3' |
| BiP shRNA1 | 5'-GGAGCGCATTGATACTAGA-3' |
| BiP shRNA2 | 5'-GAAATCGAAAGGATGGTTAAT-3' |
| HSPA8 shRNA1 | 5'-TCAACTGGCTTGATAAGAATC-3' |
| HSPA8 shRNA2 | 5'-TCCATTACCCGTGCCCGATTT-3' |
| Nix shRNA | 5′-GCAGCAATGGCAATGATAA-3′ |
| DELE1 shRNA | 5′-GCTCCGTCCTCATCTCTAAAC-3' |
| GPAM shRNA | 5′-CCCAATCTTCAGTACCTTGAT-3' |
| SLC44A1 shRNA | 5′-GCATTGGGATGGGATTTATTT-3' |
| CHOP shRNA1 | 5′-TGAACGGCTCAAGCAGGAAAT-3' |
| CHOP shRNA2 | 5′-CTGCACCAAGCATGAACAATT-3' |
| PINK1 shRNA1 | 5′-GGACGCTGTTCCTCGTTATGA-3' |
| PINK1 shRNA2 | 5′-GAGTATGGAGCAGTCACTTAC-3' |

**The primer sequences for real-time PCR (5′-3′)**

| GSDMA RT S | GAGGAAGAGGAAGAGCACGC |
| --- | --- |
| GSDMA RT AS | GTTCCCTTCACCTTCACCGT |
| GSDMB RT S | CTGAAAAGCGACCGGCAATA |
| GSDMB RT AS | CTTCCCTTTGGCCCTTGTGT |
| NDUFS4 RT S | ATGGAGATTGGCACAGGACC |
| NDUFS4 RT AS | GCGAGCAGGAACAAAGATCC |
| RISP RT S | TGGTCCTGCTGGGTTTGTTT |
| RISP RT AS | CTCCCAGGGGAAAATGGACC |
| COX4I1 RT S | GGCAGAATGTTGGCTACCAG |
| COX4I1 RT AS | AGACAGGTGCTTGACATGGG |
| ATP5PD RT S | CCAAAATGGCTGGGCGAAAA |
| ATP5PD RT AS | TTGGCCACATTGGCCTTGTA |
| HSPA8 RT S | ACCTACTCTTGTGTGGGTGTT |
| HSPA8 RT AS | GACATAGCTTGGAGTGGTTCG |
| Nix RT S | ATGTCGTCCCACCTAGTCGAG |
| Nix RT AS | TGAGGATGGTACGTGTTCCAG |
| DELE1 RT S | GAGCTCTTCCCCGTACACTG |
| DELE1 RT AS | CATGGGGACCTGACCTGTCG |
| GPAM RT S | GATGTAAGCACACAAGTGAGGA |
| GPAM RT AS | TCCGACTCATTAGGCTTTCTTTC |
| SLC44A1 RT S | GGACCGTAGCTGCACAGAC |
| SLC44A1 RT AS | GCCACAAATAAATCCCATCCCA |
| CHOP RT S | GGAAACAGAGTGGTCATTCCC |
| CHOP RT AS | CTGCTTGAGCCGTTCATTCTC |
| PINK1 RT S | CCCAAGCAACTAGCCCCTC |
| PINK1 RT AS | GGCAGCACATCAGGGTAGTC |

**Synthesis of compound DdBIC**


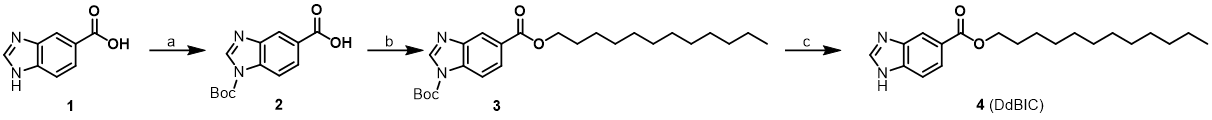


Reagents and conditions: (a) Boc_2_O (1.2eq), Et_3_N (1.2eq), DMF, 50°C, 12 h; (b) HOBT (1.1eq), EDCI (1.1eq), 1-Dodecanol (1eq), Et_3_N (1.1eq), DCM, 0°C-25°C, 12 h; (c) TFA (1.5eq), DCM, 0°C ~ 25°C, 6 h.

All reagents were purchased and used without further purification unless otherwise noted. Reactions were monitored by thin-layer chromatography (TLC) on thin-layer YanTai silica gel GF-254 plates. ^1^H NMR and ^13^C NMR spectra were obtained with a Bruker Avance III 600 MHz spectrometer (600 MHz for ^1^H and 150 MHz for ^13^C). Chemical shifts are expressed in *δ* values (ppm) using tetramethylsilane (TMS) as the internal standard; coupling constants (*J*) are given in Hz. The signal multiplicities are characterized as s (singlet), d (doublet), t (triplet), and m (multiplet). High-resolution mass spectra (HRMS) were acquired on a Q-Exactives series MS instrument with a UV detector at 254 nm in low-resonance electrospray mode (ESI). The purity of all the tested compounds was 97.84%, as determined by HPLC (Agilent 1260 Infinity III Prime, UV detection at λ = 254 nm) analysis on a YMC-Pack-ODS-AQ C18 column (4.6 × 250 mm, 5 μm), which was eluted at 1 mL/min with an ACN and water (80-100 : 20-0) solvent system.

Synthesizing compound (**2**): A solution of 1H-benzo[d]imidazole-5-carboxylic acid (2.5 g, 15.4 mmol) and Boc_2_O (4.0 g, 18.5 mmol) in anhydrous DMF (20 mL) was added to Et_3_N (2.6 mL, 18.5 mmol) dropwise, which was then heated to 50°C and stirred for 12 h. After cooling to 25°C, Sat NaHCO_3_ (30 mL) was added to the mixture to adjust the pH to 8, which was then extracted with ethyl acetate (30 mL), combined with the aqueous phase, adjusted to pH 3 with 1 N HCl, extracted with ethyl acetate (20 mL × 3) and then dried with Na_2_SO_4_ and concentrated in vacuo to yield a colorless oil (4 g, yield: 98%) without purification. ^1^H NMR (600 MHz, CDCl_3_) *δ* (ppm): 8.56 (s, 1H), 8.54 (s, 1H), 8.18 (d, *J* = 8.4 Hz, 1H), 8.08 (d, *J* = 8.5 Hz, 1H), 1.72 (s, 9H); LCMS *m/z*: 261.0 [M-H]^-^.

Synthesizing Dodecyl-1H-benzo[d]imidazole-5-carboxylate (**4**): A mixture of compound **2** (4 g, 15.4 mmol), HOBT (2.6 g, 17 mmol) and EDCI (3.2 g, 17 mmol) in DCM (40 mL) was stirred at 0°C for 1 h, and then, 1-dodecanol (2.9 g, 15.4 mmol) and Et_3_N (2.3 mL, 17 mmol) were added to the mixture, which was warmed to 25°C and stirred for 12 h. The reaction was quenched with Sat NaHCO_3_ and saltwater, dried over Na_2_SO_4_ and concentrated in vacuo. The crude product was purified by column chromatography with petroleum ether and ethyl acetate to yield compound **3** (5.0 g, yield: 75%) as a colorless oil. To a solution of **3** (5.0 g, 11.6 mmol) in DCM (25 mL), TFA (1.3 mL, 17.4 mmol) was added at 0°C under Ar, and the mixture was stirred at 25°C for 6 h. The mixture was poured into water and extracted with ethyl acetate. The diluted solution was washed with water (2 × 30 mL) and saltwater (3 × 30 mL), dried with Na_2_SO_4_ and concentrated in vacuo. The residue was purified via column chromatography with petroleum ether and ethyl acetate (PE/EA = 1:9) to yield compound **4** (1.8 g, 47.3%) as a white solid. Mp = 110-112°C. ^1^H NMR (600 MHz, CDCl_3_) *δ* (ppm): 9.61(s, 1H), 8.96(s, 1H), 8.47 (s, 1H), 8.14 (dd, *J* = 8.7, 1.2 Hz, 1H), 7.76 (d, *J* = 8.6 Hz, 1H), 4.32 (t, *J* = 6.7 Hz, 2H), 1.83-1.69 (m, 2H), 1.48-1.38 (m, 2H), 1.32 (dd, *J* = 14.5, 7.2 Hz, 2H), 1.29-1.19 (m, 14H), 0.85 (t, *J* = 7.0 Hz, 3H). ^13^C NMR (150 MHz, CDCl_3_) *δ* (ppm): 166.23, 144.50, 138.40, 135.57, 125.62, 124.85, 117.41, 115.38, 65.23, 31.75, 29.48, 29.47, 29.43, 29.40, 29.17, 29.12, 28.64, 25.96, 22.56, 14.41. HRMS (ESI): *m/z* calcd for C_20_H_30_N_2_O_2_ [M+H] ^+^ 331.2380, found 331.2375. HPLC purity: 97.84%, tr = 9.5 min.

**Synthesis of the DdBIC-P probe**

**Dodecyl 1-(but-3-yn-1-yl)-1H-benzo[d]imidazole-5-carboxylate**

Cs_2_CO_3_ (100 mg, 0.3 mM) was added to a suspension of dodecyl 1H-benzo[d]imidazole-5-carboxylate (30 mg, 0.09 mmol) in DMF (1 mL), and the mixture was stirred for 0.5 h. 4-Bromobut-1-yne (40 μL, 0.5 mmol) was then added, and the mixture was stirred at 100°C for 14 h. The mixture was diluted with water (5 mL) and extracted with ethyl acetate (5 mL × 3). The combined organic layers were washed with saturated saline (5 mL) and dried with anhydrous sodium sulfate to obtain the crude product, which was purified via silica gel column chromatography (elution with petroleum ether/ethyl acetate = 1/1), and dodecyl 1-(but-3-yn-1-yl)-1H-benzo[d]imidazole-5-carboxylate was obtained. White solid, 3.3 mg; yield, 10%. ^1^H NMR (600 MHz, CDCl_3_) *δ* (ppm): 8.55 (s, 1 H), 7.93 -8.22 (m, 2 H), 7.44 (d, *J* = 8.44 Hz, 1 H), 4.25 - 4.50 (m, 4 H), 3.66 (d, *J* = 3.30 Hz, 1 H), 2.77 (td, *J* = 6.60, 2.57 Hz, 2 H), 1.67 - 1.80 (m, 4 H), 1.26 - 1.35 (m, 16 H), 0.79 - 0.90 (m, 3 H). LCMS *m/z*: 383.6 [M + H]^+^.


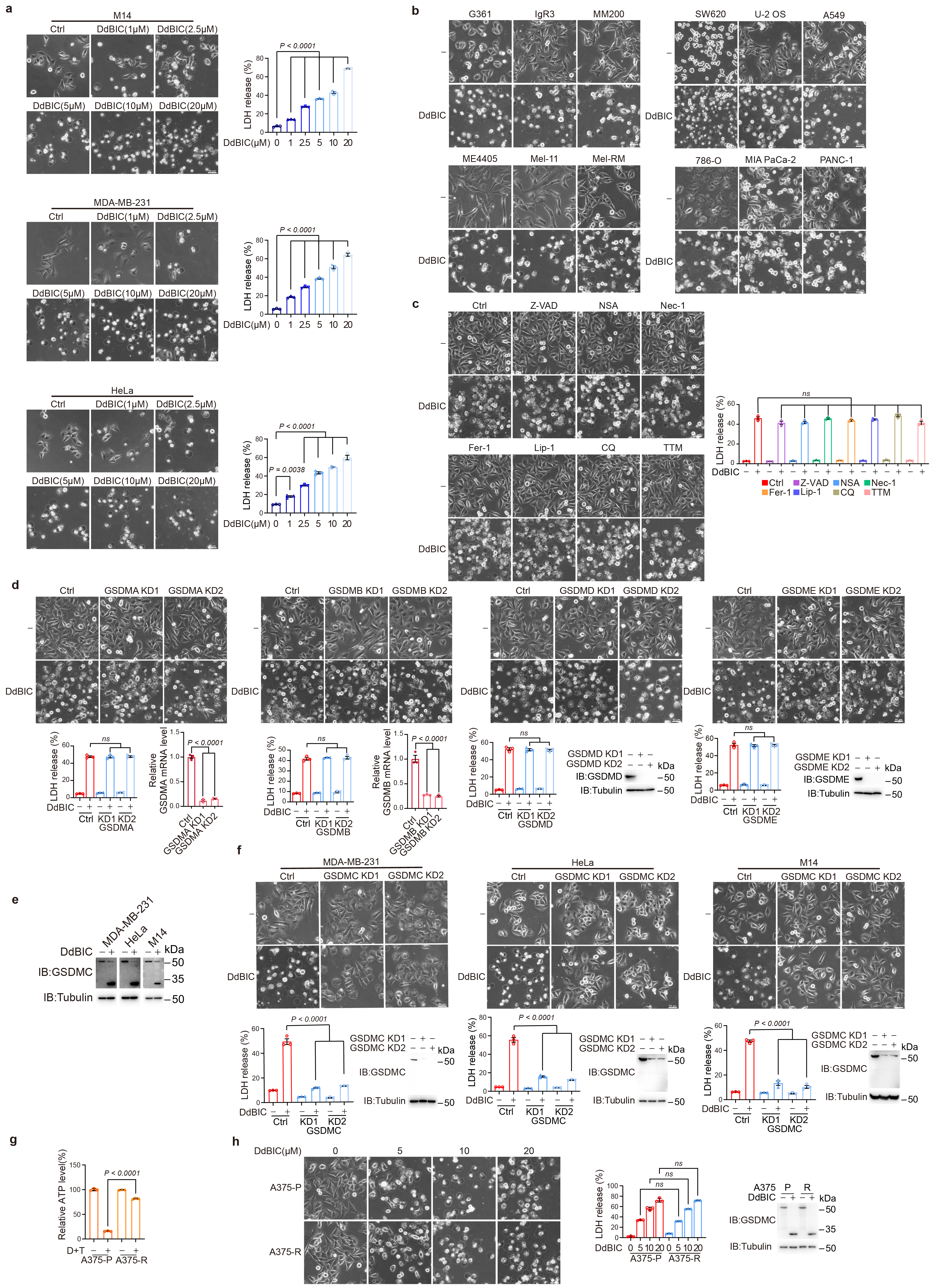
Supplementary Figure 1

**(a)** Effects of different DdBIC concentrations on pyroptotic induction in three cancer cell lines. Cells were treated with DdBIC (20 μM) for 8 h, pyroptotic morphologies and LDH levels were detected. **(b)** DdBIC is a broad-spectrum inducer of pyroptosis. Different cancer cell lines, including melanoma (G361, IgR3, MM200, ME4405, Mel-11 and Mel-RM), colon cancer cell line SW620, osteosarcoma cell line U-2OS, non-small cell lung cancer cell line A549, kidney clear cell carcinoma cell line 786-O, pancreatic cancer cell lines MIA-PaCa-2 and PANC-1, were treated with DdBIC (20 μM) for 8 h, pyroptotic morphologies were indicated. **(c)** Other inhibitors for cell deaths do not interfere with DdBIC-induced pyroptosis. Melanoma A375 cells were cotreated with various inhibitors and DdBIC (20 μM) for 8 h, pyroptotic morphologies and LDH levels were shown. **(d)** Knockdown of other gasdermin proteins does not interfere with DdBIC-induced pyroptosis. GSDMA, GSDMB, GSDMD and GSDME were separately knocked down in A375 cells, and then cells were treated with DdBIC (20 μM) for 8 h. **(e)** DdBIC induces GSDMC cleavage in three cell lines. Cells were treated with DdBIC (20 μM) for 8 h. **(f)** Effects of DdBIC in pyroptosis induction on GSDMC knockdown three cell lines. Cells were treated with DdBIC (20 μM) for 8 h. **(g)** Assessment of resistance to dabrafenib (D) and trametinib (T) treatment. Cell viabilities were measured by ATP levels in parental (A375-P) and resistant (A375-R) A375 cells following 24-hour stimulation with dabrafenib plus trametinib (D+T) treatment. **(h)** DdBIC induces comparable pyroptosis in A375-P and A375-R cells. Pyroptosis and GSDMC cleavage in A375-P and A375-R cells were treated with DdBIC(20 μM) for 8 h.

The knockdown efficiencies of different genes were indicated in necessary panels. Tubulin was used to determine protein loading. All western blots were repeated at least twice. Statistical data are presented as mean ± s.e.m. of three independent experiments. Statistical analyses were performed by two-way analysis of variance (ANOVA) with Tukey’s multiple comparisons test (c, d, f, g and h), one-way ANOVA with Tukey’s multiple comparisons test (a). *P* values are indicated.

**
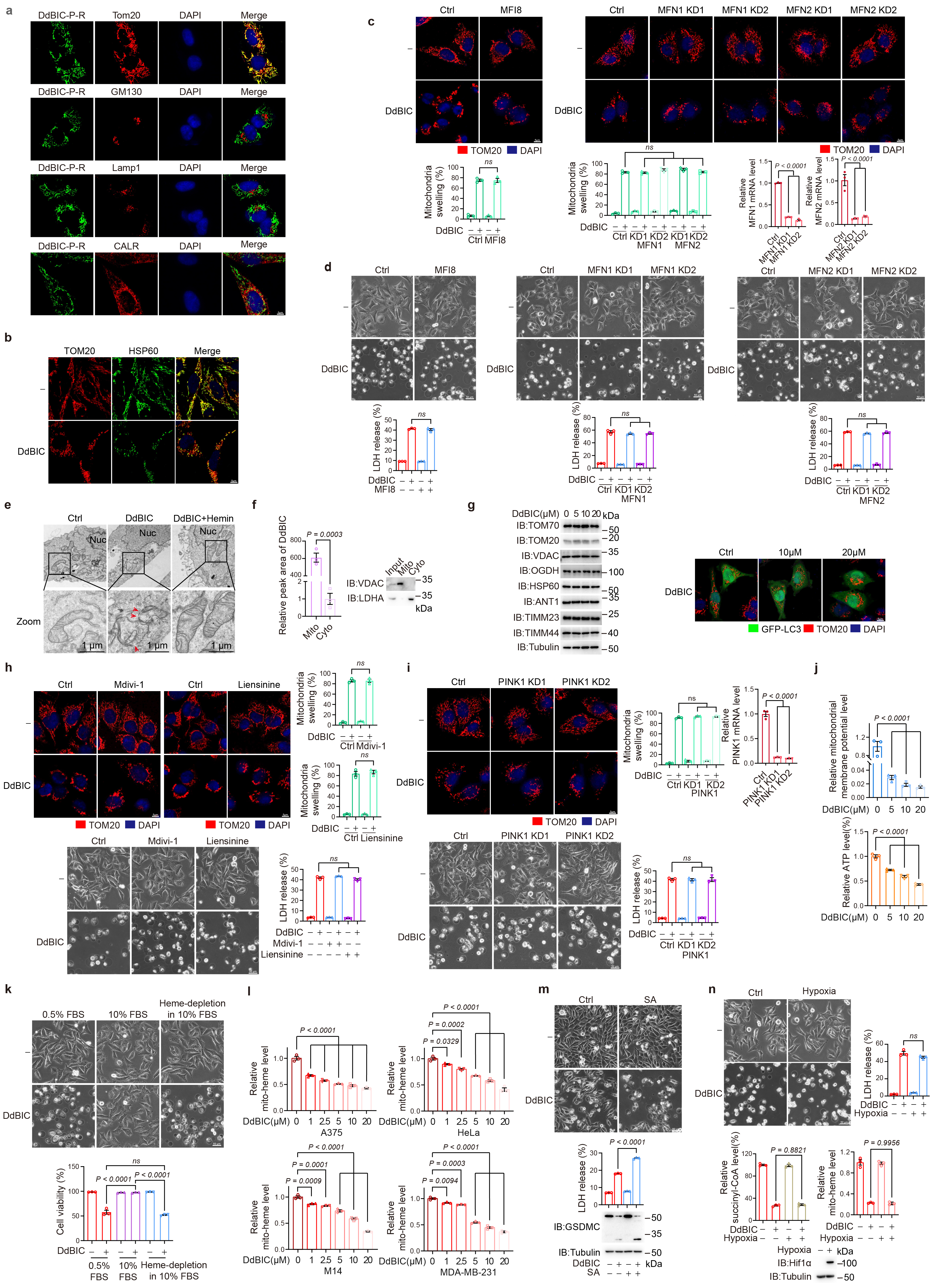
Supplementary Figure 2**

**(a)** DdBIC-P-R localized in the mitochondria (indicated by TOM20) but not in the golgi apparatus (indicated by GM130), lysosome (indicated by Lamp1) and endoplasmic reticulum (indicated by CALR). A375 cells were incubated with fluorescently-labeled DdBIC-P-R (100 μM) for 1 h. **(b)** DdBIC induces mitochondrial swelling. A375 cells were stained with Tom20 and Hsp60 after DdBIC (20 μM) treatment for 4 h, images were observed under a confocal microscope. **(c-d)** Treatment of MFI8 or knockdown of MFN1/2 does not affect mitochondrial swelling and pyroptosis. A375 cells were pretreated with MFI8 for 2 h, or subjected to MFN1/2 knockdown, followed by DdBIC (20 μM) treatment for 4 h. Mitochondrial morphology (c) and pyroptosis (d) were indicated. **(e)** DdBIC induces mitochondrial swelling and rupture. A375 cells were treated with DdBIC (20 μM) for 4 h, images of mitochondrial morphology were observed under a transmission electron microscope. **(f)** DdBIC is enriched in the mitochondrial fraction quantified by mass spectrometry. A375 cells were treated with DdBIC (20 μM) for 0.5 h. Mitochondrial and cytosolic fractions were prepared. **(g)** DdBIC does not induce mitophagy. Left, A375 cells were treated with DdBIC for 8 h to detect expression levels of mitochondrial-associated proteins as indicated. Right, confocal microscopic images of GFP-LC3 (green) and mitochondria (Tom20, red) were shown after A375 cells were treated with DdBIC (20 μM) for 4 h. **(h)** Mdivi-1 and liensinine do not inhibit mitochondrial swelling or pyroptosis. After pretreatment with mdivi-1 or liensinine for 2 h, followed by DdBIC for 4 h to observe morphology of mitochondria, and for 8 h to detect pyroptosis. **(i)** knockdown of PINK1 does not affect mitochondrial swelling and pyroptosis. PINK1 was knocked down in A375 cells and then cells were treated with DdBIC for 4 h to detect mitochondrial morphology and for 8 h to detect pyroptosis. **(j)** DdBIC reduces mitochondrial membrane potential and ATP levels in a dose-dependent manner. Mitochondrial membrane potential and intracellular ATP levels in A375 cells were measured after cells were treated with DdBIC (20 μM) for 4 h. **(k)** DdBIC-induced pyroptosis is associated with heme depletion. A375 cells were cultured in different concentrations of FBS with or without depletion of FBS-derived heme, and then treated with DdBIC (20 μM) for 8 h to analyze pyroptotic morphology and cell viability. **(l)** DdBIC inhibits mitochondrial heme levels in a dose-dependent manner in four cancer cell lines. Cells were treated with DdBIC for 4 h. **(m)** Succinylacetone (SA) cooperates with DdBIC to induce pyroptosis. A375 cells were pretreated with SA for 12 h and then with DdBIC (20 μM) for another 7 h to detect pyroptosis. **(n)** Hypoxia shows no effect on DdBIC-induced decrease of mito-heme and succinyl-CoA levels, as well as pyroptosis. A375 cells were cultured under hypoxia (0.1% O_2_) for 4 h to detect mito-heme level or 1 h to detect succinyl-CoA level in the presence of DdBIC (20 μM).

The knockdown efficiencies of different genes were indicated in necessary panels. Tubulin was used to determine protein loading. All western blots were repeated at least twice. Statistical data are presented as mean ± s.e.m. of three independent experiments. Statistical analyses were performed by two-way analysis of variance (ANOVA) with Tukey’s multiple comparisons test (c, d, h, i, k, m and n), one-way ANOVA with Tukey’s multiple comparisons test (c, i, j and l) and Unpaired two-tailed Student’s t-test (f). *P* values are indicated.


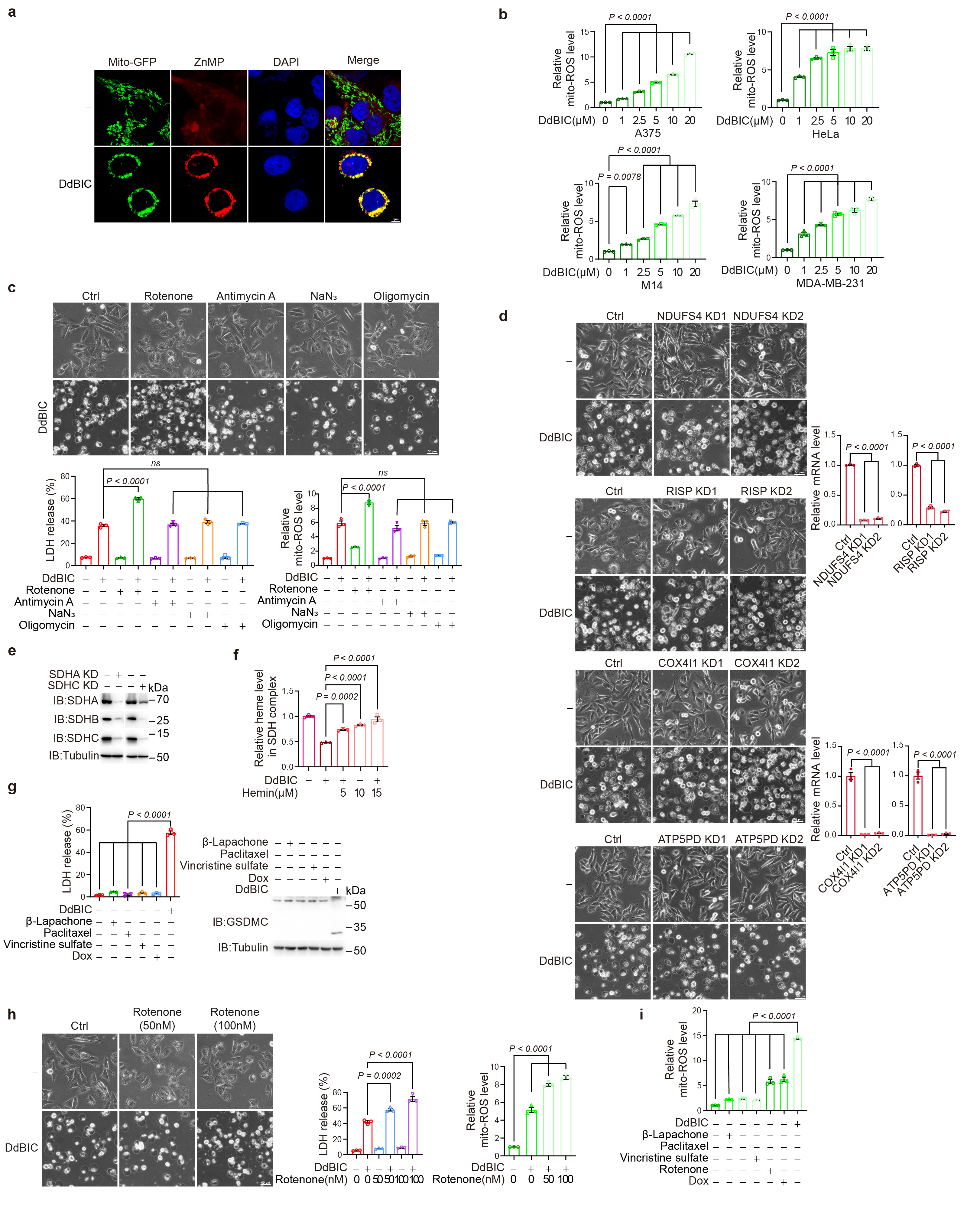


**Supplementary Figure 3**

**(a)** Heme location in the mitochondria is shown with fluorescent heme analog (ZnMP) staining. A375 cells were cotreated with ZnMP and DdBIC (20 μM) for 4 h detect the ZnMP localization in the mitochondria. **(b)** Effects of DdBIC on levels of mitochondrial ROS in a dose-dependent manner in four cancer cell lines. Cells were treated with DdBIC for 4 h. **(c)** The inhibitors to electron transport chain (ETC)-related complexes have no effects on DdBIC-induced pyroptosis and mito-ROS. A375 cells were cotreated with DdBIC (20 μM) and various inhibitors (including rotenone (100 nM), antimycin A (5 μM), NaN3 (15 mM) and oligomycin (1 μM)) for 4 h to detect mito-ROS level, and 8 h to show pyroptotic morphology. **(d)** Knockdown of corresponding components in various ETC-related complexes respectively shows no effects on DdBIC-induced pyroptosis. Various genes were knocked down in A375 cells, and cells were treated with DdBIC (20 μM) for 8 h to indicate pyroptotic morphology. **(e)** Knockdown of SDHA or SDHC impairs entire SDH complex stability. SDHA or SDHC was knocked down in A375 cells and expression levels of SDHA, SDHB and SDHC were analyzed. **(f)** Hemin increases heme level in SDH complex in the presence of DdBIC. A375 cells were cotreated with heme and DdBIC (20 μM) at indicated doses for 4 h. SDH complexes were prepared for heme level detection. **(g)** Mito-ROS activators do not induce GSDMC-mediated pyroptosis. A375 cells were treated with dox, β-lapachone, paclitaxel, vincristine sulfate, or DdBIC as indicated for 8 h. LDH release and GSDMC cleavage were indicated. **(h)** Rotenone promotes DdBIC-induced mito-ROS production and pyroptosis. A375 cells were treated with DdBIC alone or in combination with rotenone for 4 h, mito-ROS and pyroptosis were detected. **(i)** DdBIC exhibits a stronger ability to elevate mito-ROS levels compared to other mito-ROS inducers. Mito-ROS levels were measured in A375 cells after treatment with different reagents for 5 h.

The knockdown efficiencies of different genes were indicated in necessary panels. Tubulin was used to determine protein loading. All western blots were repeated at least twice. Statistical data are presented as mean ± s.e.m. of three independent experiments. Statistical analyses were performed by two-way analysis of variance (ANOVA) with Tukey’s multiple comparisons test (c, f and h), one-way ANOVA with Tukey’s multiple comparisons test (b, d, g, h and i). *P* values are indicated.


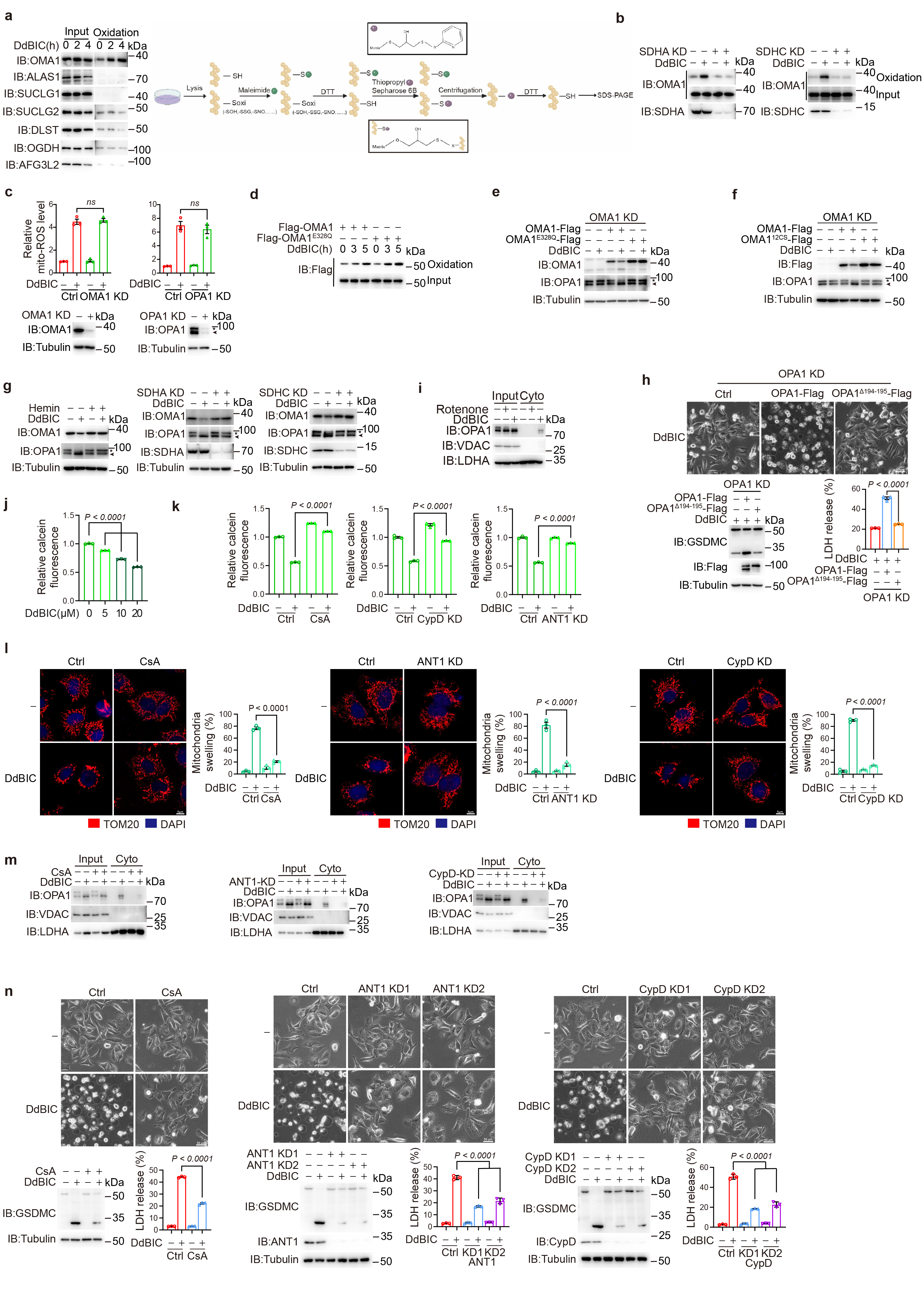


**Supplementary Figure 4**

**(a)** DdBIC induces OMA1 oxidation. Left, A375 cells were treated with DdBIC (20 μM) for 4 h. Different proteins as indicated were prepared, oxidations were detected. Right, the procedure for oxidation detection. The assay for OMA1 oxidation was based on resin-assisted capture of S-oxidated proteins. **(b)** Knockdown of SDHA or SDHC impairs DdBIC-induced OMA1 oxidation. SDHA and SDHC were separately knocked down in A375 cells, cells were treated with DdBIC (20 μM) for 4 h to analyze OMA1 oxidation. **(c)** Knockdown of OMA1 or OPA1 does not affect DdBIC-induced mito-ROS. OMA1 or OPA1 was separately knocked down in A375 cells, after which cells were treated with DdBIC (20 μM) for 4 h to detecte levels of mito-ROS. **(d)** Oxidation level of OMA1^E328Q^ is comparable to that of OMA1. OMA1 or OMA1^E328Q^ was separately transfected into A375 cells. OMA1 oxidations were analyzed after treatment with DdBIC (20 μM) for 4 h. **(e-f)** Enzymatic activity and oxidation of OMA1 on DdBIC-induced OPA1 cleavage. OMA1 and mutant OMA1^E328Q^ (e) or OMA1^12CS^ (f) were reintroduced into OMA1 knockdown A375 cells, cells were then treated with DdBIC (20 μM) for 4 h to detect OPA1 cleavage. **(g)** Effects of hemin or knockdown of SDH on OMA1 activity and OPA1 cleavage. A375 cells were treated with DdBIC (20 μM) with hemin (5 μM) (left), or SDHA and SDHC were separately knocked down first (right). After treatment with DdBIC (20 μM) for 4 h, OMA1 activity and OPA1 cleavage were analyzed. **(h)** OPA1 involved in pyroptotic induction depends on its cleavage. OPA1 or mutant OPA1^Δ194-195^ was reintroduced into OPA1 knockdown A375 cells, cells were then treated with DdBIC (20 μM) for 8 h to detecte pyroptosis. **(i)** Rotenone treatment alone does not induce OPA1 release to the cytosol. Immunoblot of OPA1 in cytosolic fraction was isolated from A375 cells that were treated with rotenone or DdBIC for 5 h. **(j)** DdBIC induces mPTP opening. mPTP opening was assessed by calcein-AM/CoCl₂ quenching assay in A375 cells that were treated with DdBIC for 4 h. **(k-n)** CsA, knockdown of CypD or ANT1 inhibits mPTP opening, mitochondrial swelling, cytosolic localization of S-OPA1 and pyroptosis. mPTP opening (k), mitochondrial swelling (l), cytosolic localization of S-OPA1 (m) and pyroptosis (n) were assessed in A375 cells that were pretreated with CsA or subjected to CypD or ANT1 knockdown prior to DdBIC treatment.

The efficiencies of knockdown and overexpression different genes were indicated in necessary panels. Tubulin was used to determine protein loading. All western blots were repeated at least twice. Statistical data are presented as mean ± s.e.m. of three independent experiments. Statistical analyses were performed by two-way analysis of variance (ANOVA) with Tukey’s multiple comparisons test (c, k, l and n) and one-way ANOVA with Tukey’s multiple comparisons test (h and j). *P* values are indicated.


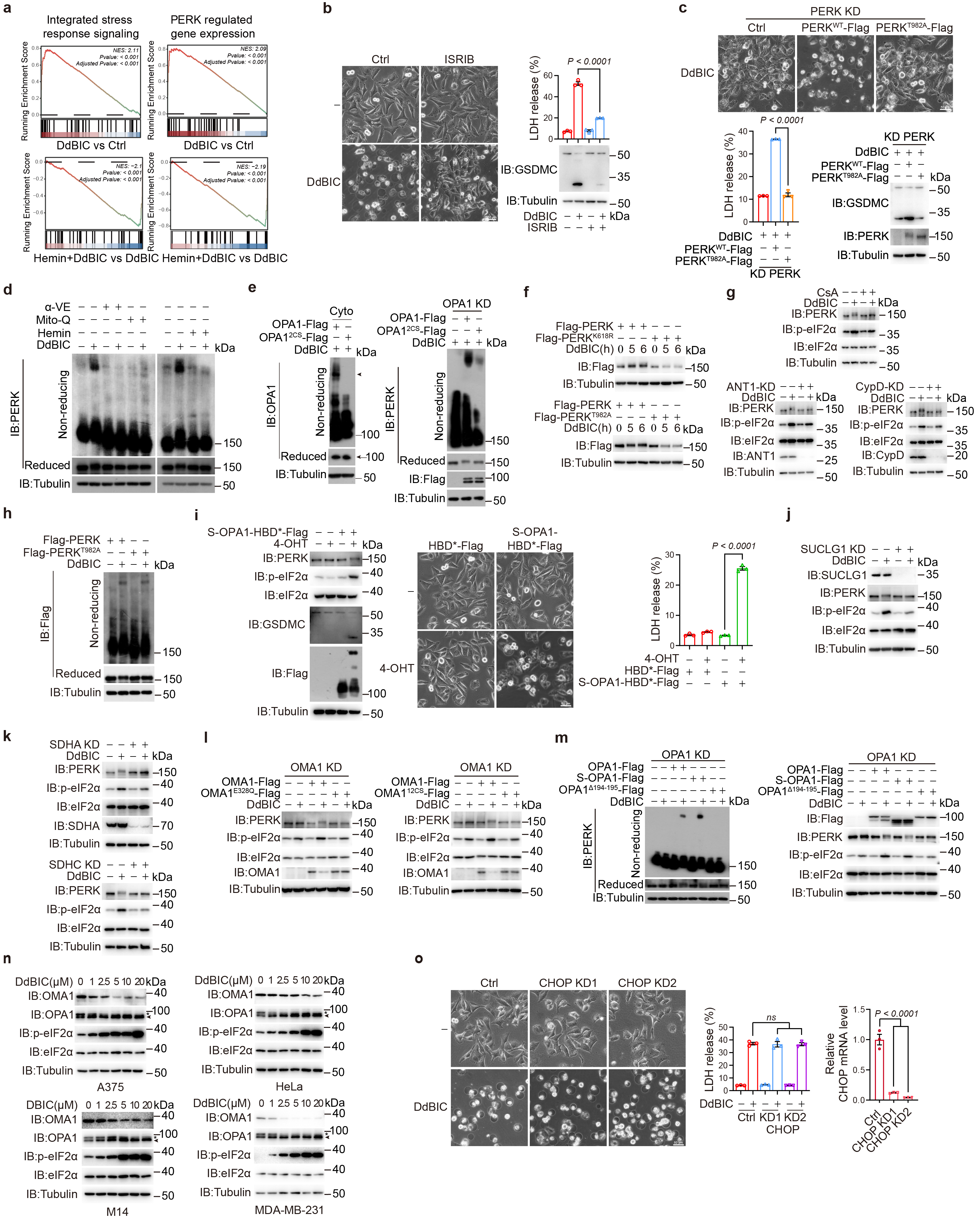


**Supplementary Figure 5**

**(a)** Analysis of gene set enrichment (GSEA). DdBIC-regulated stress response (ISR) signaling and PERK-regulated gene expression (top) and effects of hemin on these two processes (bottom) were shown. **(b)** Effect of ISRIB on DdBIC-induced pyroptosis. Cells were cotreated with ISRIB (5 μM) and DdBIC (20 μM) for 8 h, pyroptosis was detected. **(c)** PERK^T982A^ mutant inhibits DdBIC-induced pyroptosis. After DdBIC treatment for 8 h in PERK-knockdown A375 cells constituted with either PERK^WT^ or PERK^T982A^, pyroptosis and GSDMC cleavage were detected. **(d)** Effect of different reagents on PERK dimerization. Cells were cotreated with DdBIC (20 μM) and either α-VE (25 μM), Mito-Q (0.5 μM), or hemin (5 μM) as indicated for 5 h to detect PERK dimerization. **(e)** Dimerization mutation of OPA1 fails to promote the dimerization of PERK. Left, OPA1 or its mutant OPA1^2CS^ was overexpressed in A375 cells, cells were then treated with DdBIC (20 μM) for 5 h to detect the dimers of OPA1. Right, OPA1 and OPA1^2CS^ was reintroduced into OPA1-knockdown A375 cells, PERK dimers were detected. **(f)** DdBIC triggers self-phosphorylation of PERK. PERK^WT^, PERK^K618R^ or PERK^T982A^ were transfected into A375 cells, and cells were then treated with DdBIC (20 μM) for 5 h to detect the phosphorylation of PERK (indicated by up-shift band). **(g)** CsA treatment or knockdown of either CypD or ANT1 inhibits activation of ISR. Phosphorylation levels of PERK and eIF2α were assessed in A375 cells those were pretreated with CsA (5 μM, 2 h) or subjected to CypD or ANT1 knockdown prior to DdBIC treatment for 5 h. **(h)** Effects of PERK phosphorylation on DdBIC-induced dimerization of PERK. PERK or PERK^T982A^ was transfected into A375 cells. After cells were treated with DdBIC (20 μM) for 5 h, the dimers of PERK were detected. **(i)** Effect of S-OPA1 on pyroptotic induction. S-OPA1 fused with HBD* was transfected into A375 cells, and then treated with 4-OHT. Dimerization of S-OPA1, phosphorylation of PERK and eIF2α (treat with 4-OHT for 1 h) and pyroptosis (treat with 4-OHT for 4 h) were detected. **(j-k)** Knockdown of different genes abolishes DdBIC-induced phosphorylations of PERK and eIF2α. SUCLG1 (j) or SDHA and SDHC (k) were separately knocked down in A375 cells that were then treated with DdBIC (20 μM) for 5 h, phosphorylation levels of PERK and eIF2α were determined. **(l)** Effects of OMA1 mutants on DdBIC-induced phosphorylations of PERK and eIF2α. OMA1 or its mutants OMA1^E328Q^ (enzyme-inactive) and OMA1^12CS^ (non-oxidation) were separately reintroduced in the OMA1 knockdown A375 cells that were treated with DdBIC (20 μM) for 5 h, phosphorylation levels of PERK and eIF2α were detected. **(m)** Effects of OPA1 mutants on DdBIC-induced dimerization and phosphorylation of PERK. OPA1 or its deletion mutants S-OPA1 and OPA1^Δ194-195^ were separately reintroduced in OPA1 knockdown A375 cells that were then treated with DdBIC (20 μM) for 5 h, dimers of PERK (left) and phosphorylation levels of PERK and eIF2α (right) were detected. **(n)** Effects of DdBIC on OMA1 activity, OPA1 cleavage, and eIF2α phosphorylation. Different doses of DdBIC were used to treat cells as indicated for 5 h, OMA1 activity, OPA1 cleavage, and eIF2α phosphorylation were detected. **(o)** Knockdown of CHOP does not inhibit pyroptosis. CHOP was knocked down in A375 cells, and cells were treated with DdBIC for 8 h to detect the pyroptosis.

The efficiencies of knockdown and overexpression different genes were indicated in necessary panels. Tubulin was used to determine protein loading. All western blots were repeated at least twice. Statistical data are presented as mean ± s.e.m. of three independent experiments. *P* values are indicated. Statistical analyses were performed by two-way analysis of variance (ANOVA) with Tukey’s multiple comparisons test (b, i and o) and one-way ANOVA with Tukey’s multiple comparisons test (c and o). *P* values are indicated.

**
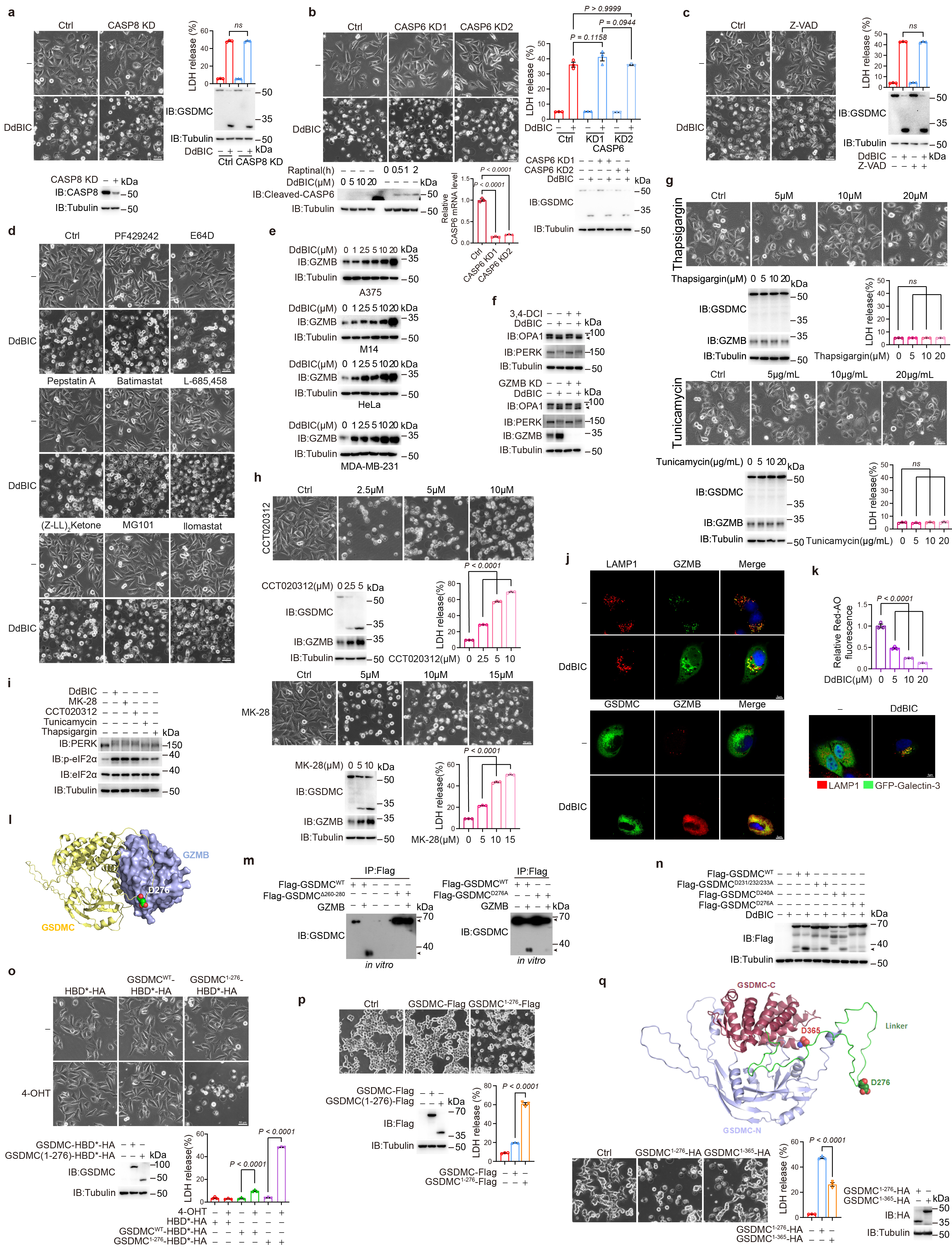
**

**Supplementary Figure 6**

**(a-b)** Effect of caspase-8 or caspase-6 on DdBIC-induced pyroptosis. Caspase-8 or caspase-6 was separately knocked down in A375 cells, and cells were treated with DdBIC (20 μM) for 8 h to detect pyroptosis (a). DdBIC was used to treat A375 cells with indicated concentrations for 6 h, and cleaved-caspase-6 could not be detected. Raptinal was used as a positive control (b). **(c)** Effect of Z-VAD on DdBIC-induced pyroptosis. A375 cells were cotreated with Z-VAD (40 μM) and DdBIC (20 μM) for 8 h to detect pyroptosis. **(d)** Effects of different protease inhibitors on DdBIC-induced pyroptosis. A375 cells were cotreated with DdBIC (20 μM) and various protease inhibitors, including E64D (100 μM), MG101 (2 μM), pepstatin A (200 μM), batimastat (60 μM), L-685,458 (10 μM), llomastat (20 μM), PF429242 (60 μM) and (Z-LL)_2_Ketone (20 μM) for 8 h to detect pyroptotic morphologies. **(e)** DdBIC elevates GZMB expression levels in a dose-dependent manner across four cancer cell lines. Different concentrations of DdBIC were used to treat cells for 6 h, GZMB expression levels were determined. **(f)** 3,4-DCI treatment or knockdown of GZMB fails to reverse DdBIC-induced cleavage of OPA1 and activation of PERK. A375 cells were cotreated with inhibitor 3,4-DCI (10 μM) and DdBIC (20 μM) or GZMB was knocked down in cells. After DdBIC treatment for 5 h, OPA1 cleavage and PERK phosphorylation were shown. **(g)** Thapsigargin or tunicamycin treatment does not induce GZMB and GSDMC-mediated pyroptosis. Cells were incubated with thapsigargin or tunicamycin for 24 h, GSDMC cleavage, GZMB expression and pyroptosis were shown. **(h)** Treatment of CCT020312 or MK-28 activates GZMB-GSDMC pyroptotic pathway. A375 cells were treated with CCT020312 or MK-28 for 8 h, GSDMC cleavage, GZMB expression and pyroptosis were indicated. **(i)** Comparative activation of PERK-eIF2α axis. A375 cells were treated with DdBIC, CCT020312, MK-28, thapsigargin and tunicamycin for 5 h, and phosphorylation levels of PERK and eIF2α were detected. **(j)** DdBIC promotes GZMB release from lysosome-related organelles (LROs) to the cytosol and colocalized with GSDMC. Top, confocal microscopy showed GZMB and LAMP1 colocalization. Bottom, image of GZMB and GSDMC colocalization were shown in A375 cells that were treated with DdBIC for 7 h. **(k)** DdBIC induces LMP. Top, quantification of AO-red fluorescence intensity. Bottom, colocalization of GFP-Galectin-3 and LAMP1 in A375 cells that were treated with DdBIC for 6 h. **(l)** Molecular modeling for GSDMC binding to GZMB. GSDMC-GZMB complex with the best score in the docking experiments was shown. The GSDMC (accession number AF-Q9BYG8-F1) is colored in yellow ribbon and GZMB (accession number 1IAU) is in light blue surface. **(m)** Cleavage of GSDMC^WT^, GSDMC^Δ260-280^ and GSDMC^D276A^ by recombinant GZMB protein *in vitro*. GSDMC^WT^, GSDMC^Δ260-280^ and GSDMC^D276A^ were transfected into HEK293T cells. Flag-tagged immunoprecipitates were incubated with recombinant GZMB proteins and cleavages of GSDMC were determined. **(n)** Effects of several GSDMC mutants on the cleavage of GSDMC. Flag- GSDMC^WT^ or its mutans GSDMC^D231/232/233A^, GSDMC^D240A^ or GSDMC^D276A^ was separately transfected into A375 cells. After cells were treated with DdBIC (20 μM) for 8 h, cleavage of GSDMC was determined. **(o)** N-terminus of GSDMC (GSDMC-1–276-HBD*-HA) induces pyroptosis. A375 cells were transfected with either GSDMC-WT-HBD*-HA or GSDMC-1–276-HBD*-HA first, and then treated with 4-OHT (3 μM) for 2 h, pyroptosis and LDH release were analyzed. **(p)** Effect of GSDMC^1-276^ on pyroptosis in 293T cells. GSDMC or N-terminus of GSDMC (GSDMC^1-276^) was transfected into cells and pyroptosis was detected. **(q)** Top, D276 is located in the hinge region adjacent to the N-terminal domain, whereas D365 resides within the C-terminal domain of GSDMC. Bottom, GSDMC^1-365^ or GSDMC^1-276^ was transfected into 293T cells and pyroptosis was detected.

The efficiencies of knockdown and overexpression different genes were indicated in necessary panels. Tubulin was used to determine protein loading. All western blots were repeated at least twice. Statistical data are presented as mean ± s.e.m. of three independent experiments. Statistical analyses were performed by two-way analysis of variance (ANOVA) with Tukey’s multiple comparisons test (a-c and o) and one-way ANOVA with Tukey’s multiple comparisons test (g, h, k, p and q). *P* values are indicated.

**
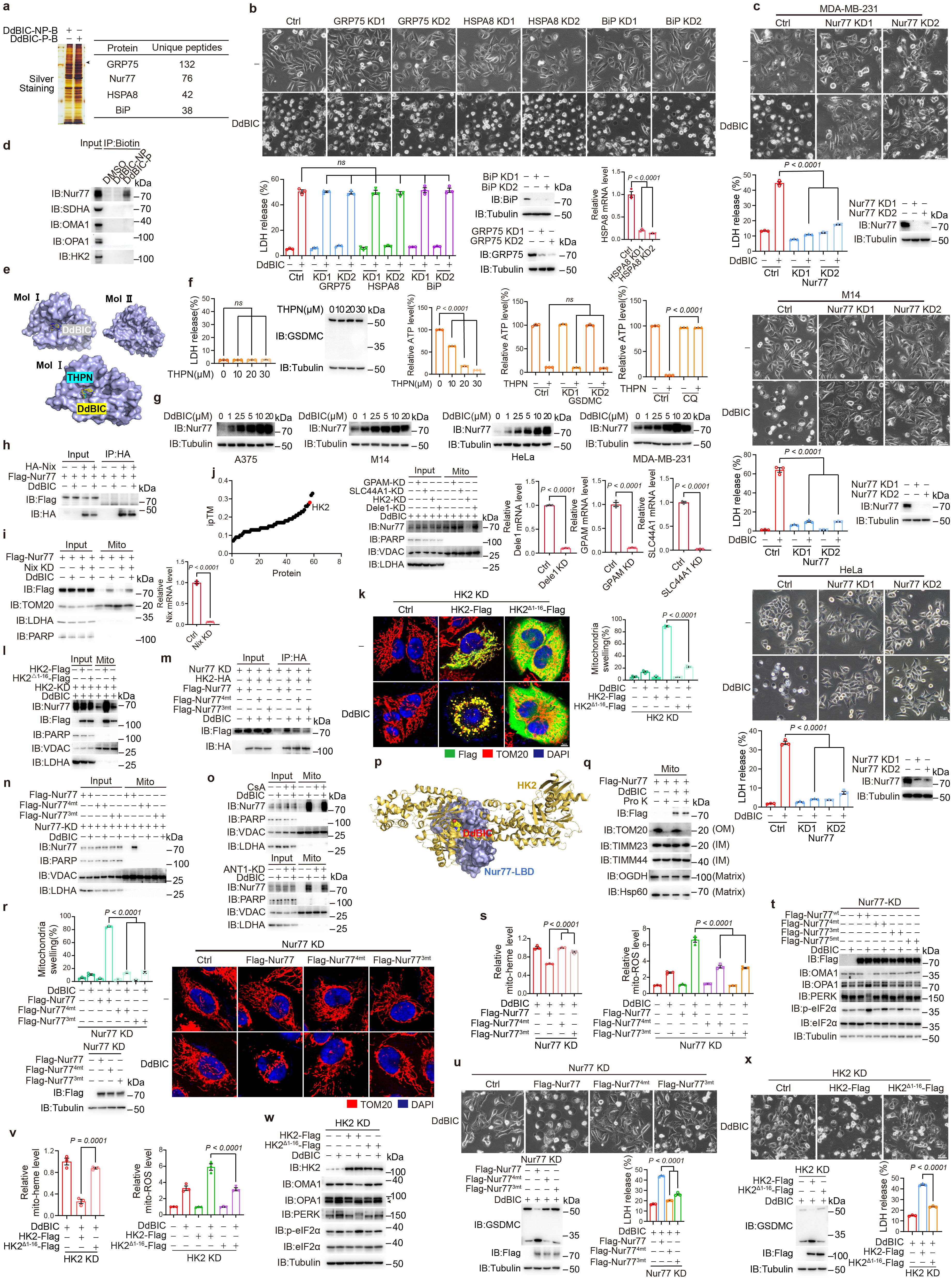
**

**Supplementary Figure 7**

**(a)** Identification of the DdBIC target. Left, cell lysates were incubated with DdBIC-P (100 μM) for 2 h, and then the biotin was attached with a click chemistry. Proteins were immunoprecipitated via NeutrAvidin beads and then subjected to SDS-PAGE. The silver staining showed a unique band (indicated by arrow). Right, potential proteins of DdBIC binding were identified by mass spectrometry. **(b)** Proteins, including GRP75, HSPA8, and BiP, are not involved in pyroptotic induction. Different proteins were separately knocked down in A375 cells. After cells were treated with DdBIC (20 μM) for 8 h, pyroptosis was detected. **(c)** Knockdown of Nur77 attenuates DdBIC-induced pyroptosis across three cancer cell lines. Nur77 was knocked down in cells as indicated, and pyroptosis was detected. **(d)** DdBIC binds to Nur77. Cells were treated with DdBIC-P (100 μM) or DdBIC-NP (100 μM) for 0.5 h. Biotin was attached by a click chemistry in cell lysates. Proteins were immunoprecipitated via NeutrAvidin beads to detect participants in DdBIC-induced signaling pathway for pyroptosis. **(e)** DdBIC binds to Nur77 LBD. Molecules are shown as light blue surface. LBD was of two conformations determined by X-ray crystallography. In Molecule I (top left) with an open conformation, DdBIC bound in a specific pocket. Molecule II (top right) with no pocket in the same location and does not bind DdBIC in the crystal. Comparison of DdBIC binding with THPN, there is 90 degrees difference in their head orientation. DdBIC is in yellow stick, and electron density is in blue chicken wire. The 2Fo-Fc electron density map is contoured at 1.0 δ. Bottom, the difference between DdBIC and THPN binding to Nur77. **(f)** THPN induces autophagy not pyroptosis. ATP level, LDH release, and GSDMC cleavage were indicated in A375 cells that were treated with THPN for 24 h. ATP levels were detected in GSDMC-knockdown A375 cells, or cells were cotreated with chloroquine (CQ) followed by THPN treatment for 24 h. **(g)** DdBIC induces Nur77 expression in a dose-dependent manner. Different doses of DdBIC were used to treat cells for 5 h, Nur77 expression levels were detected. **(h-i)** Nix does not interact with Nur77 for translocation into the mitochondria. Nur77 and Nix were cotransfected into A375 cells that were then treated with DdBIC (20 μM) for 0.5 h. Interaction between Nur77 and Nix was detected (h). In Nix knockdown A375 cells Nur77 was reintroduced, cells were treated with DdBIC (20 μM) for 0.5 h, Nur77 translocation to the mitochondria was determined (i). **(j)** Identification of proteins mediating Nur77 mitochondrial translocation. Left, the binding affinity between conformational-changed Nur77 LBD and mitochondrial outer membrane proteins (with available crystal structures covering >50% of the sequence) is predicted via AlphaFold 3. Middle, mitochondrial translocation of Nur77 induced by DdBIC was assessed in cellular fraction prepared from A375 cells after knockdown of four candidate proteins as indicated. Right, efficiencies of knockdown different genes were indicated. **(k)** HK2 mitochondrial localization is crucial for mitochondrial swelling induced by DdBIC. In HK2 knockdown A375 cells, HK2 or its mutant HK2^Δ1-16^ was reintroduced into cells that were then treated with DdBIC (20 μM) for 4 h, mitochondrial locations of HK2 and mitochondrial morphologies were observed under a confocal microscope. The numbers of mitochondrial swelling were quantified. **(l)** Deletion of HK2 sequence for targeting mitochondria HK2^Δ1-16^ blocks Nur77 translocation to the mitochondria. HK2 or HK2^Δ1-16^ were reintroduced into HK2 knockdown A375 cells. After treatment with DdBIC (20 μM) for 0.5 h, mitochondrial fractions were prepared and endogenous Nur77 was detected. **(m-n)** Effects of Nur77 mutants on its interaction with HK2 and mitochondrial location. In Nur77 knockdown A375 cells, HK2, Nur77 or its mutants Nur77^4mt^ and Nur77^3mt^ were transfected into A375 cells. After cells were treated with (20 μM) for 0.5 h, interaction between Nur77 and HK2 was detected (m), mitochondrial fractions were prepared to detect Nur77 localization (n). **(o)** CsA treatment or knockdown of ANT1 does not inhibit mitochondrial location of Nur77. A375 cells were pretreated with CsA (5 μM) for 2 h or subjected to ANT1 knockdown prior to DdBIC treatment for 0.5 h. **(p)** A docking model of HK2/Nur77-LBD complex. DdBIC is shown as a yellow sphere. **(q)** DdBIC triggers Nur77 translocation into the mitochondria. A375 cells were transfected with Flag-Nur77 and then treated with DdBIC (20 μM) for 0.5 h. The mitochondrial fractions were prepared and digested by Proteinase K for western blot analysis. **(r-u)** Effects of Nur77 mutants on mitochondrial morphology, mito-heme levels and mito-ROS levels, activations of OMA1 and ISR and pyroptosis. Nur77, Nur77^4mt^, Nur77^3mt^, or Nur77^5mt^ were reintroduced into Nur77 knockdown A375 cells, and cells were treated with (20 μM) for 4 h to determine mitochondrial morphology, mitochondrial heme and mito-ROS levels (s), the numbers of mitochondrial swelling were also quantified (r), for 5 h to detect the activation of the OMA1-OPA1-PERK axis (t), and for 8 h to detect pyroptosis (u). **(v-x)** Effects of HK2 mutants on mitochondrial heme and mito-ROS levels, activation of OMA1, ISR and pyroptosis. HK2 or HK2^Δ1-16^ were reintroduced into HK2 knockdown A375 cells, and cells were then treated with DdBIC (20 μM) for 4 h to detect mito-heme and mito-ROS levels (v), for 5 h to detect activation of OMA1-OPA1-PERK axis (w), and for 8 h to indicate occurrence of pyroptosis (x).

The efficiencies of knockdown and overexpression different genes were indicated in necessary panels. Tubulin was used to determine protein loading. All western blots were repeated at least twice. Statistical data are presented as mean ± s.e.m. of three independent experiments. Statistical analyses were performed by two-way analysis of variance (ANOVA) with Tukey’s multiple comparisons test (b, c, f, k, r, s and v), one-way ANOVA with Tukey’s multiple comparisons test (b, f, s, u, v and x) and Unpaired two-tailed Student’s t-test (i and j). *P* values are indicated.


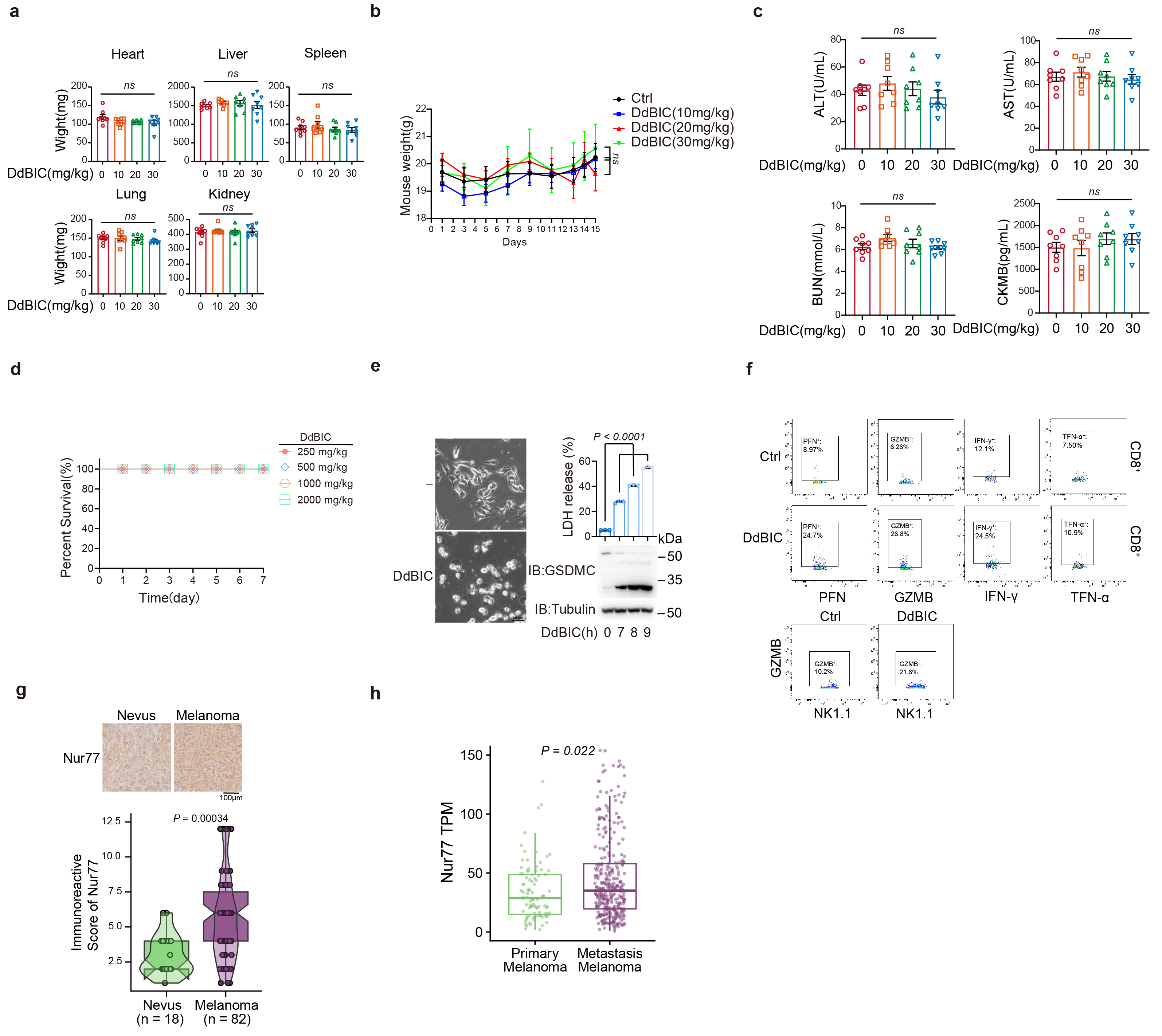


**Supplementary Figure 8**

**(a-b)** Effect of DdBIC on body and organ weights. After the xenograft tumors developed to the diameter about 3 ~ 4 mm, mice were intraperitoneal injection with DdBIC every other day for 2 weeks. Mice were sacrificed to record weights of different organs (a) and body weights (b). **(c)** DdBIC does not induce organ toxicities. Blood was collected via retro-orbital bleeding and serum biomarkers for organ injury (ALT, AST, BUN, CK-MB) were measured in mice (n = 8) after i.p. injection of DdBIC (20 mg/kg every other day for 2 weeks). **(d)** Acute toxicity of DdBIC in mice. Survival curves of mice following a single intraperitoneal (i.p.) injection with indicated doses of DdBIC were indicated. Mice were monitored for 7 days (n = 7). **(e)** DdBIC induces pyroptosis in B16 cells. Cells were treated with DdBIC (20 μM) for indicated times and pyroptosis was detected. **(f)** Flow cytometry plots illustrate perforin (PFN), granzyme B (GZMB), tumor necrosis factor alpha (TNF-α) and interferon-gamma (IFN-γ) expressions in CD8^+^ T cells. GZMB expression in NK cells within tumor microenvironment was also shown. **(g)** Nur77 expression in melanoma is higher than that in non-melanoma nevus. Top, representative immunohistochemistry (IHC) images of Nur77 expression in human melanoma and non-melanoma nevus specimens. Bottom, quantification of Nur77 IHC immunoreactive score (IRS) in melanoma (n = 82) and non-melanoma nevus tissues (n = 18). **(h)** Nur77 expressed higher in metastatic melanoma than that in primary melanoma. Analysis of Nur77 mRNA expression levels in primary melanoma samples versus metastatic melanoma samples from the TCGA database were demonstrated.

Tubulin was used to determine protein loading. Western blots were repeated at least twice. Statistical data are presented as mean ± s.e.m. of three independent experiments. Statistical analyses were performed by one-way ANOVA with Tukey’s multiple comparisons test (a, b, c and e) and Unpaired two-tailed Student’s t-test (g and h). *P* values are indicated.

**Supplementary Table 1.**

**Crystallographic data collection and refinement statistics.**

|  | DdBIC-bounded Nur77 LBD |
| --- | --- |
| **Data Collection** |  |
| wavelength(Å) | 0.979 |
| space group | P2_1_2_1_2_1_ |
| cell dimensions |  |
| *a, b, c*(Å) | 74.52, 76.09, 128.27 |
| α, β, γ(°) | 90.0, 90.0, 90.0 |
| resolution(Å) | 50-2.57 |
| *R*sym or *R*merge | 0.095 |
| *I/*σ*I* | 2.14 (at 2.57Å) |
| Completeness (%) | 95.8（96.5） |
| Redundancy | 3.1（3.1） |
| **Refinement** |  |
| Resolution (Å) | 33.36-2.57 |
| No. reflections | 20948 |
| Rwork/Rfree | 0.206/ 0.265 |
| No. atoms |  |
| Protein | 3582 |
| Ligand/ion | 24 |
| Water | 95 |
| *B*-factors |  |
| Protein | 45.3 |
| Ligand/ion | 77.6 |
| Water | 38.8 |
| R.m.s. deviations |  |
| Bond lengths (Å) | 0.0075 |
| Bond angles (°) | 1.4781 |

Data in parentheses are highest resolution values.
